# Light-Driven Regioselective Deoxygenation of Carbohydrate Lactones for 2-Deoxy Sugar Precursor Synthesis

**DOI:** 10.1021/acs.orglett.4c04763

**Published:** 2025-01-28

**Authors:** Justyna J. Najczuk, Wojciech Chaładaj, Bartłomiej Furman

**Affiliations:** Institute of Organic Chemistry, Polish Academy of Sciences, Kasprzaka 44/52, Warsaw 01-224, Poland

## Abstract

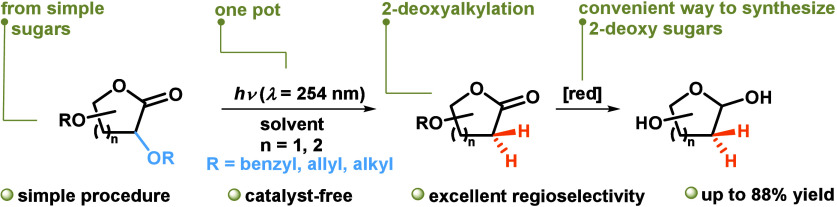

A sustainable method for the synthesis of 2-deoxy lactones as direct precursors to 2-deoxy sugars via regioselective UV-light-driven dealkyloxylation of carbohydrate-derived lactones is detailed. This catalyst- and additive-free protocol utilizes light irradiation, providing high step economy and functional group compatibility. This environmentally friendly and straightforward approach enhances the synthetic toolbox for 2-deoxy sugars, which is vital in numerous biologically active molecules and drug candidates.

Deoxy sugars are crucial components of numerous biologically active molecules, including nucleotides, antibiotics, and secondary metabolites.^1^ In 2-deoxy sugars, the hydroxyl group on the C-2 position is replaced by a hydrogen atom.^2^ The most prominent example of a 2-deoxy sugar is 2-deoxyribose, which is a key structural unit in the backbone of DNA.^3^ The absence of the C-2 hydroxyl group, compared to RNA, confers increased chemical stability to DNA, making it a more reliable carrier of genetic information.^1d^ Beyond nucleic acids, 2-deoxy sugars are integral to several natural products with significant biological activity. For instance, antibiotics such as erythromycin contain 2-deoxy sugar moieties that enhance their binding interactions with bacterial ribosomes, contributing to their therapeutic efficacy.^1d^ From a synthetic perspective, incorporating 2-deoxy sugars into drug molecules can substantially improve their pharmacokinetic properties.^4^ The removal of the hydroxyl group reduces the molecule’s polarity and hydrogen-bonding capacity, often leading to increased membrane permeability and enhanced bioavailability.^1c,5^ Additionally, 2-deoxy sugars serve as valuable glycosyl donors in glycosylation reactions, facilitating the synthesis of glycosylated natural products and their analogs.^1a^

Their widespread natural occurrence and critical role in drug design have made the efficient and selective synthesis of 2-deoxy sugars a central goal in organic chemistry.^2a^ Various methodologies have been explored for the synthesis of 2-deoxy sugars, each with distinct advantages and limitations.^1b,2^ One of the most widely used methods involves the deoxygenation of glycosyl hydroxyl groups.^1a,1b^ Traditional strategies in this area include radical-based deoxygenation reactions, such as the Barton-McCombie deoxygenation.^6^ Although these methods are effective, they typically require harsh reaction conditions, including the use of strong reducing agents, high temperatures, and prolonged reaction times. These conditions can limit the applicability of these methods, particularly in the synthesis of complex molecules. An alternative approach to the synthesis of 2-deoxy sugars involves the inversion of configuration at the C-2 position, usually achieved through the formation of an intermediate sulfonate ester followed by reduction.^7^ While this method offers greater selectivity, it often requires multiple steps and suffers from poor atom economy and the use of hazardous reagents. An alternative approach to synthesizing 2-deoxy sugars involves the addition of water or alcohol to glycals under acidic conditions.^1a^ However, this method also has limitations, including unwanted side reactions and low atomic efficiency.^8^

In recent years, photoinduced methods for the synthesis of 2-deoxy sugars have also received considerable attention.^1a,9^ One such method involves the photodeoxygenation of electron-deficient benzoates, such as *m*-(trifluoromethyl)benzoate, under UV irradiation in the presence of 9-methylcarbazole (MCZ) as a photosensitizer (Scheme 1A and 1B).^1a,10^ Despite their promise, these approaches face a significant limitation: the reliance on specially modified carbohydrate derivatives and the often required extensive photochemical process optimization, which limits the wider application of these methods. Visible-light photocatalysis is favored for its ability to drive organic transformations under mild conditions with a simple setup.^11^ While UV light is often seen as outdated and complex, modern advancements have made UV photochemical reactions as straightforward as those using visible light. As part of our long-term research into innovative photochemical transformations,^12^ we report herein a novel UV-light-driven method for the synthesis of 2-deoxy sugars via photochemical dealkyloxylation of carbohydrate-derived lactones (Scheme 1C). This approach addresses many limitations associated with traditional synthesis methods by offering a more sustainable and operationally simple alternative. By utilization of light irradiation, regioselective deoxygenation was achieved under mild conditions, without the need for catalysts or additives. This process not only improves step economy but also demonstrates good functional group compatibility, making it a versatile tool for the synthesis of 2-deoxy sugars from readily available starting materials.

**Scheme 1:**
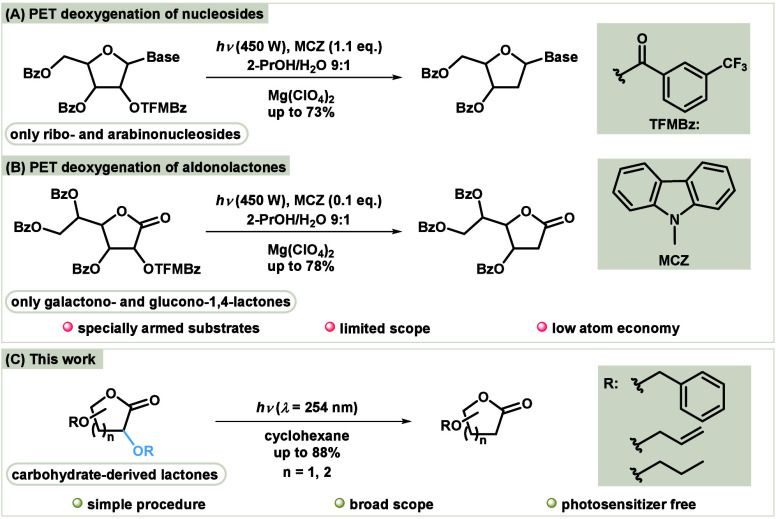
(A,B) Example of Photoinduced Electron-Transfer Deoxygenation of Benzoyl-Protected Nucleosides and Aldonolactones and (C) This Work

We began our investigation of the photodealkyloxylation of carbohydrate-based lactones by optimization of the reaction conditions through selection of the optimal source of light spanning from UV-C to UV-A (254–365 nm), solvent, and concentration (Table 1). A cheap self-made photoreactor was used in the tests, for which the construction has been detailed previously.^12a^ In optimization studies, 2,3,5-tri-*O*-benzyl-d-ribono-1,4-lactone (**1a**) was used as a model substrate. The progress of the reaction was monitored by TLC, and the reaction was stopped after complete conversion of substrate. The reaction failed to occur under irradiation with UV-A and UV-B light sources (Table 1, entries 1 and 2). Employing UV-C frequencies resulted in the formation of the major product **2a** in 56% yield in cyclohexane (Table 1, entry 3). Replacing the solvent with acetonitrile resulted in a decreased yield (Table 1, entry 4). Irradiation of a more concentrated mixture in cyclohexane led to an increase in the process efficiency to 64% for product **2a**, which represented the best outcome (Table 1, entry 5). However, increasing the concentration further decreased the reaction yield (Table 1, entry 6). At the optimal concentration, changing the solvent to acetonitrile, THF, or *t*-BuOH did not increase the yield of benzyloxylated product **2a** (Table 1, entries 7–9).

**Table 1:**
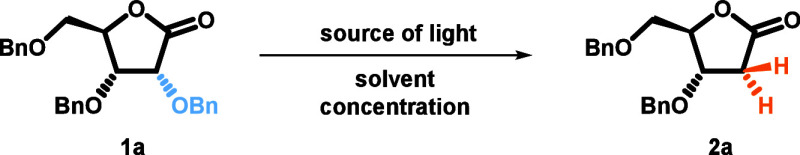
Optimization of the Reaction Conditions^a^

Entry	Solvent	*c* (M)	Light	Time (h)^b^	Yield (%)^c^
1	cyclohexane	0.006	UV-A	24	0
2	cyclohexane	0.006	UV-B	24	0
3	cyclohexane	0.006	UV-C	1	56
4	CH_3_CN	0.006	UV-C	1	46
**5**	**cyclohexane**	**0.009**	**UV-C**	**1.25**	**64**
6	cyclohexane	0.012	UV-C	1.25	55
7	CH_3_CN	0.009	UV-C	1.25	50
8	THF	0.009	UV-C	1.25	44
9	*t*-BuOH	0.009	UV-C	2	49

a Reaction conditions: **1a** (0.072 mmol) and solvent at RT. Reactions were performed in degassed solvents, in a self-made photoreactor with eight UV lamps (9 W) in quartz glass. Source of light: UV-A (320–400 nm), UV-B (280–320 nm), and UV-C (200–280 nm, λ_max_ 254 nm).

b Reaction time determined by TLC.

c Isolated yields. THF = tetrahydrofuran.

Having established the optimal reaction conditions, we next investigated the generality of the process (Table 2). Under these conditions, aldono-1,4-lactones with d-ribo-**1a**, d-arabino-**1b**, d-xylo-**1c**, d-erythro-**1d**, and d-gluco-**1e** configurations were efficiently converted to their corresponding 2-deoxy-aldono-1,4-lactones **2a**–**2e**. The deoxygenation of compounds **1a**–**1e** was highly selective with no racemization at the other stereogenic centers. Based on these promising results, we explored the scope of the reaction with respect to the alkoxy segment. We began by investigating glucuronolactones with different substitution patterns, including **1f**-**1h**. First, irradiation of benzylated derivative **1f** yielded 2-deoxybenzylated product **2f** with excellent efficiency. Therefore, the deoxygenation of OHs with other protecting groups was investigated. The allyloxy group **1g** and alkyloxy group **1h** were also efficiently removed, resulting in the same 2-deoxy product **2f** in all cases. Given the importance of 2-deoxy sugars, we extended this method to six-membered carbohydrate-based lactones. Irradiation of xylono-**1i**- and galactono-**1j**-based lactones gave the corresponding 2-benzyloxylated products **2i**–**2j** in good yields. Benzylated glucono-**1k** and rhamnono-**1l** lactone gave the expected products **2k**–**2l** with moderate efficiency, whereas 2-dealkyloxylation of *n*-propyl-gluconolactone **1m** was less efficient. The six-membered lactones (**1i**–**1m**) generally gave worse yields than the five-membered equivalents, possibly due to the smaller angle between the carbonyl and benzyl group.^13^ As shown in Scheme 2, 2-deoxy-1,4-lactone **2a**, generated through photochemical dealkyloxylation, can be directly reduced to yield the expected 2-deoxy sugar **5** in a one-pot procedure.

**Table 2:**
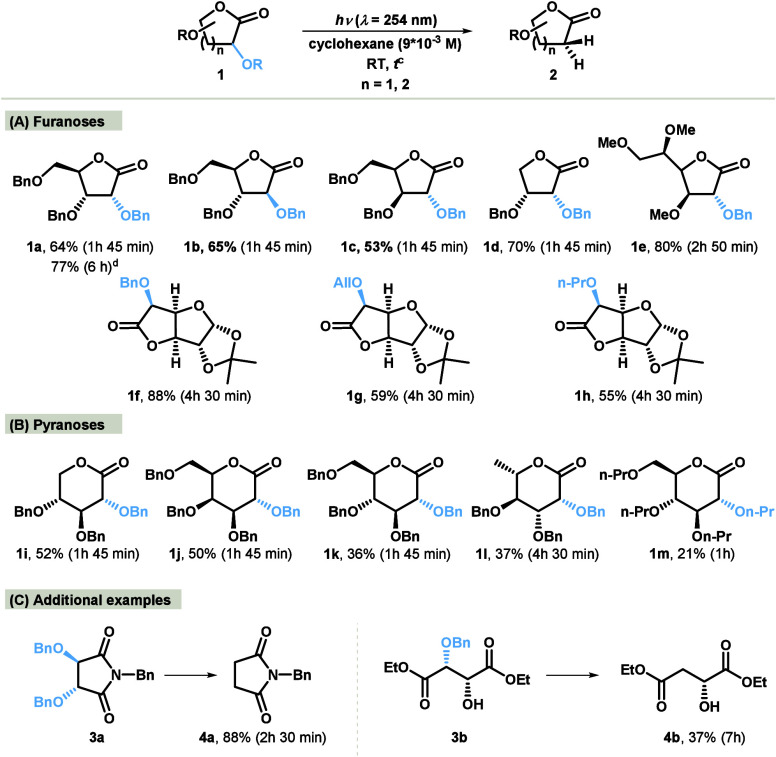
Substrate Scope^a^,^b^

a Reaction conditions: **1** or **3** (0.144 mmol) and cyclohexane (16.0 mL) at RT for 1h 45 min – 7 h under the irradiation of light (λ_max_ = 254 nm). Reactions were performed in a self-made photoreactor with eight UV-C lamps (9 W) in a quartz glass (see SI).

b Isolated yields.

c Reaction time determined by TLC.

d Reaction on 1.0 mmol scale in continuous flow system (see SI).

**Scheme 2:**
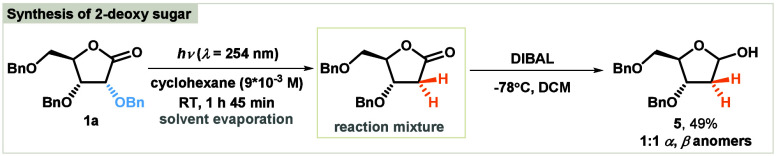
Direct Synthesis of 2-Deoxy Sugar

To further broaden the scope, we applied our deoxygenation method to structurally different compounds, cyclic imides and linear diesters (Table 2). Among the cyclic imides, dibenzyloxysuccinimide **3a** efficiently afforded *N*-benzylated succinimide **4a** under photochemical conditions. In addition, irradiation of monobenzylated tartaric acid ester **3b** resulted in successful deoxygenation, producing the debenzyloxylated product diethylmalate **4b**. Both transformations were highly efficient, demonstrating the versatility of this method for deoxygenation of different functional groups.

Several control experiments were carried out to investigate the reaction mechanism in detail. The reaction proceeded without loss of yield under nondegassed conditions (Scheme 3A), indicating that molecular oxygen is not involved. This insensitivity to oxygen supports a singlet mechanism, as oxygen is known to quench triplet states.^14^ Moreover, the addition of isoprene to the reaction mixture did not affect the reaction outcome or yield (Scheme 3B), further ruling out the involvement of a triplet intermediate. The lack of sensitivity to both isoprene and oxygen suggests that the intermediates involved are singlet species. When substrate **6** was used as the starting material, no reaction was observed (Scheme 3C), highlighting the need for protons in the gamma position relative to the carbonyl group for the reaction to occur. In addition, when the reaction was performed in acetonitrile-D_3_, no deuterium incorporation was detected in the final product **2a** (Scheme 3D). We also synthesized glucuronolactone with a deuterium-labeled benzyl group D_2_-**1f** and subjected it to the debenzyloxylation reaction. The results showed that only the unlabeled product **2f** was obtained (Scheme 3E). However, when 20 equiv of D_2_O was added and the reaction was irradiated for 1 h and 45 min, the deuterated product D-**2a** was formed (Scheme 3F). Interestingly, the treatment of **2a** with D_2_O under the same conditions did not yield D-**2a**. This suggests that hydrogen–deuterium exchange occurs, leading to the formation of −OD enol **8**, which subsequently undergoes tautomerisation to produce compound D-**2a**.

**Scheme 3:**
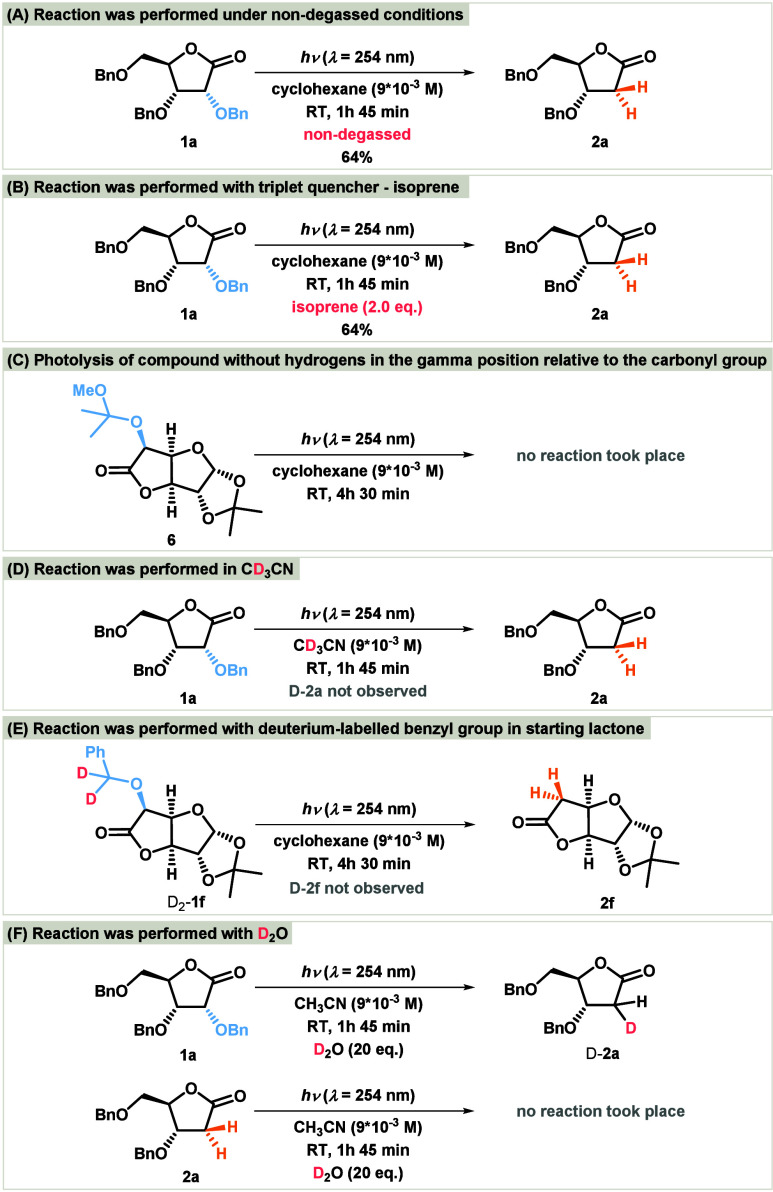
Mechanistic Studies

Based on the experimental results and previous studies,^15^ we propose a plausible reaction pathway for the UV-light-driven dealkyloxylation of carbohydrate-derived lactones (Scheme 4A). This reductive dealkyloxylation is likely to proceed via a Norrish type II reaction mechanism,^16^ which involves either a triplet or less common singlet excited state. The latter manifold, involving no spin-forbidden transitions, was postulated for systems featuring low intrinsic reaction barriers for hydrogen transfer in the S1 state.^17^ Our experimental evidence suggests that in this case the reaction may proceed via the singlet state, and we propose this as the operative pathway for the observed transformation. Upon UV irradiation, aldono-1,4-lactone **1a** is promoted to its excited state 1a*. This excitation triggers the intramolecular abstraction of the γ-hydrogen by the excited carbonyl group, resulting in the formation of the 1,4-biradical species **7**. Following the formation of the biradical, β-scission occurs, leading to the cleavage of the carbon–oxygen bond and the formation of benzaldehyde (**9**) as one product and an enol **8** as a second intermediate. The enol **8** undergoes rapid tautomerisation to give the corresponding carbonyl compound **2a**, thus completing the dealkyloxylation process. This pathway effectively explains the formation of benzaldehyde, detected as **11**, the product of a photochemical reaction with a solvent (Scheme 4B). It also supports the observed selectivity and efficiency of the reaction under photochemical conditions, further justifying the choice of cyclohexane as the optimal solvent for this transformation.

**Scheme 4:**
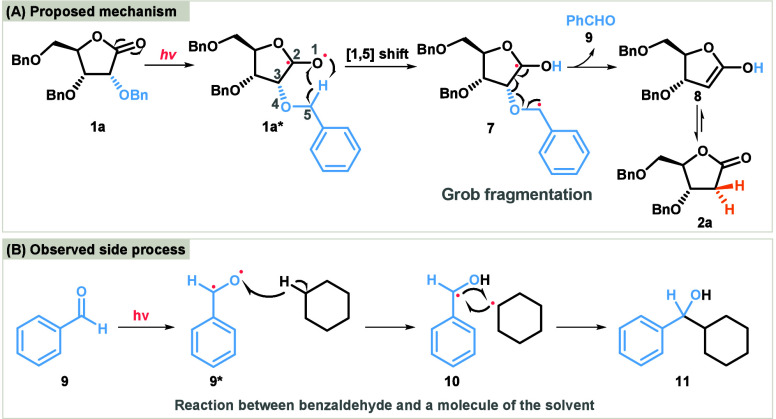
(A) Proposed Mechanism and (B) Photochemical Reaction of Benzaldehyde with Solvent Cyclohexane

Computational studies were further conducted to elucidate the mechanistic details of the photochemical cleavage of the O–C bond in model lactone **I** (Figure 1). Both singlet and triplet manifolds were considered. In the S1 state (blue paths), the 1,5-hydrogen shift leading to singlet diradical **IV** is nearly barrierless (**TS1**, Δ*G*^‡^= 9.1 kJ/mol), which is distinct from difficult Norrish type I C–C cleavage. It is also consistent with above-discussed experimental findings pointing toward operation of a singlet pathway, rather than less favorable intersystem crossing to the **T1** state. In the triplet state, both pathways are comparable, with barriers of 69.0 and 64.7 kJ/mol for [1,5]-H-shift (**TS2**) and C–C cleavage (**TS3**), respectively. The former leads to the triplet diradical **IV**, practically degenerate from its singlet counterpart. Cyclization of **IV** to cyclobutane is associated with a high activation barrier (**TS4**, Δ*G*^‡^= 148.0 kJ/mol), thus unlikely even at elevated temperature (red path). In contrast, transition state **TS5** for fragmentation toward benzaldehyde and enol **VI** lies only 38.3 kJ/mol above **VI**, rendering this pathway highly probable.

**Figure 1:**
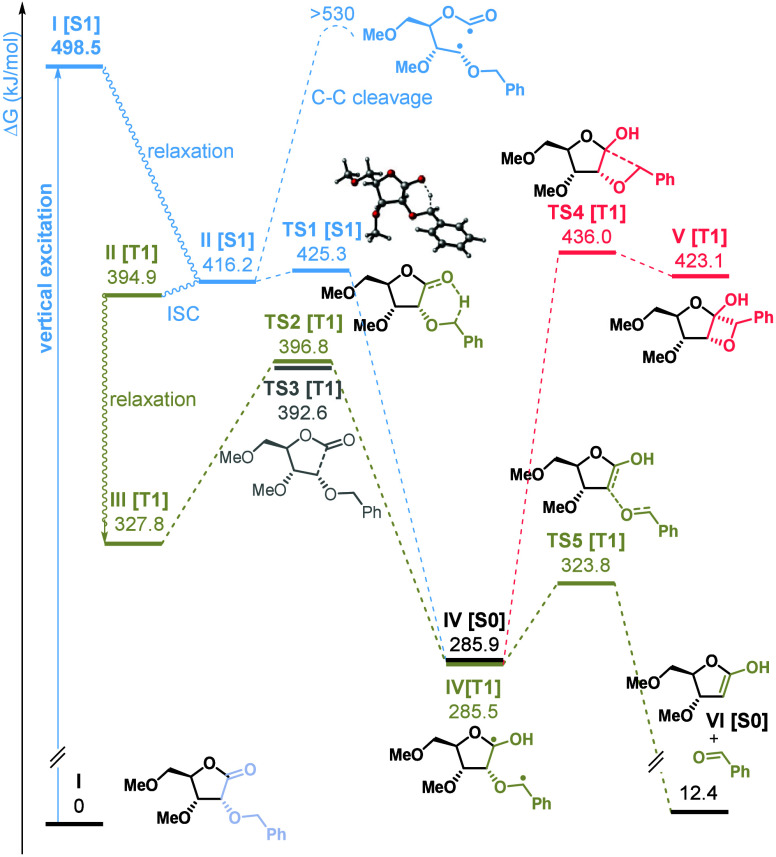
Gibbs free energy profile of the model reaction. Calculated at the M06/6-311+g(d,p)/SMD(cycloxexane)//ωB97XD/6-31g(d) level of theory (TD DFT for S1 states).

In conclusion, a versatile and sustainable method for the synthesis of 2-deoxy lactones via UV-light-driven photochemical processes has been developed. By expanding the synthetic utility of carbohydrate-derived lactones, this method introduces a valuable novel approach to 2-deoxy sugar synthesis, which aligns with green chemistry principles and underscores the significance of UV light efficiency in contemporary synthetic organic chemistry. The results also suggest that this light-induced chemical reaction represents an unusual variation of the Norrish Type II process, which can occur in a singlet state.

## Data Availability

The data underlying this study are available in the published article and its Supporting Information.

## Supporting Information Available

General information, detailed experimental procedures, characterization data for all new compounds and NMR spectra, as well as DFT calculation details (PDF)
