# Supplementary material for: Light-Driven Regioselective Deoxygenation of Carbohydrate Lactones for 2-Deoxy Sugar Precursor Synthesis
Source: Org Lett. 2025 Jan 28;27(5):1221–5. doi: 10.1021/acs.orglett.4c04763 (PMC11812017; doi:10.1021/acs.orglett.4c04763)
Supplement: Supplementary file 1 — ol4c04763_si_001.pdf [file ol4c04763_si_001.pdf]

Electronic Supplementary Information

for

Light-Driven Regioselective Deoxygenation of Carbohydrate Lactones for 2-Deoxy Sugar Precursor Synthesis

Justyna J. Najczuk, Wojciech Chaładaj and Bartłomiej Furman*

Institute of Organic Chemistry, Polish Academy of Sciences,
Kasprzaka 44/52, 01-224 Warsaw, Poland, email: bartlomiej.furman@icho.edu.pl

Table of contents

1. General Remarks 2
2. Synthesis of starting substrates 3
3. Photochemical synthesis of carbohydrate-derived 2-deoxylactones 9
   3.1 General procedure for 2-deoxylactones synthesis 9
   3.2 Cyclohexyl(phenyl)methanol (11) 15
   3.3 Procedure for 1.0 mmol scale reaction 15
4. Direct synthesis of 2-deoxy sugar 16
5. Mechanistic studies and control experiments 17
   5.1 UV-VIS absorption spectrum 17
   5.2 Control experiments 17
      5.2.1 Reaction was performed in non-degassed conditions 17
      5.2.2 Reaction was performed with triplet quencher – isoprene 17
      5.2.3 Reaction was performed with compound without hydrogens in the gamma position relative to the carbonyl group 18
      5.2.4 ^{1}H NMR spectra of the reaction mixtures 18
      5.2.5 Irradiation of 2a with UVC light 20
   5.3 Deuterium-labelling experiments 21
      5.3.1 Reaction was performed in CD_3CN 21
      5.3.2 Reaction was performed using deuterium-labelled benzyl group in starting lactone 21
      5.3.3 Reaction was performed with D_2O 25
6. DFT 28
   6.1 General 28
   6.2 Optimized geometries, energies and corrections to thermodynamic functions 28
7. Copies of ^{1}H and ^{13}C NMR spectra of substrates and products 48
8. References 68

1. General Remarks

The reagents used in this study were purchased from Sigma-Aldrich, Carbosynth, Alfa Aesar, or TCI Chemicals and were employed without further purification. Reactions involving air- and moisture-sensitive materials were carried out under an argon atmosphere in oven-dried glassware with magnetic stirring. The reactions requiring higher temperatures were heated using a heating mantle. Reagents and solvents for photochemical reactions were weighed in quartz test tubes within an MBraun UniLab Pro glove box under a nitrogen atmosphere. The test tubes were then removed from the glove box and transferred to the photoreactor. Dry solvents, including tetrahydrofuran (THF), acetonitrile (CH_3CN), and dichloromethane (CH_2Cl_2), were obtained from a Solvent Purification System (MBraun SPS 5/7), while dimethylformamide (DMF) was purchased from Sigma-Aldrich.

Column chromatography was performed using Kieselgel (230–400 mesh), and analytical thin-layer chromatography (TLC) was carried out on silica gel 60 F254 aluminum plates (Merck). Visualization was achieved using UV light or charring with either Pancaldi reagent ($(NH_4)_6MoO_4$, $Ce(SO_4)_2$, H_2SO_4, H_2O) or potassium permanganate solution ($KMnO_4$, K_2CO_3, NaOH, H_2O). Full characterization of all new compounds, including ^{1}H NMR, ^{13}C NMR, IR, MS, and optical rotation (OR), was provided.

NMR analyses were performed with Varian Mercury 400 MHz, Varian VNMRS 500 MHz and 600 MHz spectrometers. Chemical shifts are reported in parts per million (ppm) and were calibrated using residual solvent signals ($CDCl_3$: δ (H) = 7.26, δ (C) = 77.0; CD_3CN: δ (H) = 1.94) or tetramethylsilane (TMS). Infrared spectra (IR) were recorded on a JASCO FT/IR-6200 spectrophotometer and are reported in wavenumbers (cm^{-1}). High-resolution mass spectra (HRMS) were recorded using Synapt G2-S HDMS (Waters) mass spectrometer equipped with an electrospray ionization (ESI) or atmospheric-pressure chemical ionization (APCI) with time-of-flight (TOF) mass analyzer or AutoSpec Premier (Waters) mass spectrometer equipped with an electron impact (EI) ion source with the EBE double focusing geometry mass analyzer and are reported in m/z. A UV-VIS absorption spectrum was recorded on a Cary 100E spectrophotometer. Optical rotations were measured using a polarimeter. Melting points were determined with an MPMH2 apparatus and are uncorrected.

Photochemical reactions were conducted in a custom-built photoreactor equipped with eight lamp sockets, temperature control, a mixing system, and a cooling system. The electronic design allowed individual or simultaneous activation of each of the four lamp sets, enabling modulation of light intensity. The photoreactor can operate at three distinct wavelengths: UV-A (320–400 nm), UV-B (290–320 nm), and UV-C (100–290 nm). Details of the reactor's construction are provided in the supplementary information of one of our group's publications.[1]

2. Synthesis of starting substrates

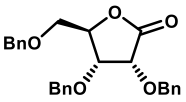

1a

2,3,5-Tri-*O*-benzyl-D-ribono-1,4-lactone (**1a**)

Compound **1a** commercial available from Carbosynth, CAS 55094-52-5.

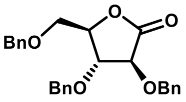

1b

2,3,5-Tri-*O*-benzyl-D-arabino-1,4-lactone (**1b**)

The synthesis of compound **1b** has been previously reported. It was synthesized according to an established protocol.[2] The product was obtained as a white solid (1.16 g, 69%).

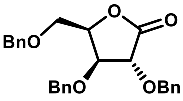

1c

2,3,5-Tri-*O*-benzyl-D-xylono-1,4-lactone (**1c**)

The synthesis of compound **1c** has been previously reported. It was synthesized according to an established protocol.[2] The product was obtained as a yellowish oil (4.0 g, 78%).

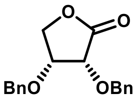

1d

2,3-Di-*O*-benzyl-D-erythronolactone (**1d**)

The synthesis of compound **1d** has been previously reported. It was synthesized according to an established protocol.[3] The product was obtained as a white solid (1.44 g, 46%).

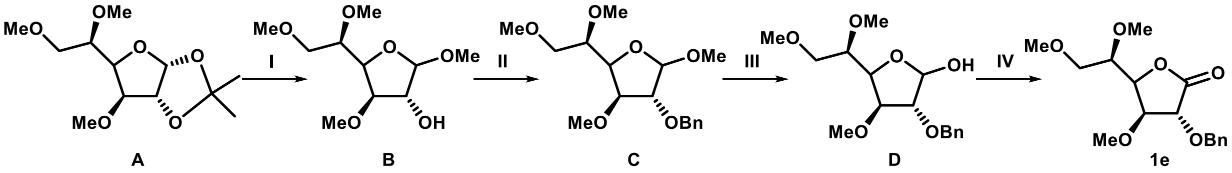

2-*O*-Benzyl-3,5,6-tri-*O*-methyl-D-glucono-1,4-lactone (**1e**)

I. To compound **A**[4] (782.0 mg, 3.13 mmol) 2.5% methanolic H_2SO_4 (7.5 mL) was added and heated under reflux for 1 h. The mixture was cooled to room temperature, neutralised with saturated Na_2CO_3 (15 mL), extracted with AcOEt (3 x 10 mL), dried (Na_2SO_4), filtered and concentrated under reduced pressure. The residue crude mixture was used without further purification in the next step.
II. To a stirring solution of the **B** from the previous step in anhydrous DMF (30 mL) was added sodium hydride (187.8 mg, 4.7 mmol) at 0 °C under argon atmosphere. After 1 h, BnBr (0.56 mL, 4.7 mmol) was added at 0 °C, and the mixture was warmed to ambient temperature and stirred for 16 h.

Subsequently, the reaction was quenched by addition of methanol (5 mL), the solvents were removed under reduced pressure and the residue was taken up in H_2O/CH_2Cl_2 (1:1). The product in the aqueous layer was extracted with CH_2Cl_2 (3 x 5 mL). The organic phase was washed with brine, dried (Na_2SO_4), filtered and concentrated under reduced pressure. The residue crude mixture was used without further purification in the next step.

III. The residue from the previous step was heated under reflux for 16 h with 1M H_2SO_4 (4.4 mL), AcOH (4.4 mL) and 1.4-dioxane (4.4 mL). The mixture was cooled to room temperature and water was added, extracted with CH_2Cl_2 (3 x 10 mL), washed with saturated $NaHCO_3$ and brine, dried (Na_2SO_4), filtered and concentrated under reduced pressure. The residue crude mixture was used without further purification in the next step.

IV. A mixture of acetic anhydride (3.0 mL) and dimethyl sulfoxide (6.3 mL) was added to the residue from the previous step and stirred at room temperature for 16 h. Water (20 mL) was added and the product extracted with AcOEt (3 x 10 mL), washed with saturated $NaHCO_3$ and brine, dried (Na_2SO_4) and concentrated in vacuo. The crude product was purified by column chromatography using 15-30% MTBE/hexanes solvent system to give the title compound **1e** as a yellow oil (211.6 mg, 22% after the all reaction steps).

$[\alpha]^{20}_D$: +75.3 (*c* = 1.0, $CHCl_3$).

^{1}H NMR (500 MHz, $CDCl_3$) δ 7.39-7.29 (m, 5H), 4.95 (d, *J* = 11.6 Hz, 1H), 4.73-4.66 (m, 2H), 4.17 (d, *J* = 3.5 Hz, 1H), 4.02 (dd, *J* = 5.3, 3.5 Hz, 1H), 3.72-3.66 (m, 2H), 3.55 (m, 1H), 3.45 (s, 3H), 3.39 (s, 6H).

^{13}C NMR (126 MHz, $CDCl_3$) δ 172.8, 136.7, 128.5, 128.1 (2C), 81.2, 78.6, 77.7, 76.7, 72.4, 71.1, 59.3, 58.7, 58.2.

IR (film) $\tilde{\nu}$: 3032, 2984, 2934, 2893, 2832, 1791, 1455, 1359, 1197, 1113, 1028, 1007, 915 cm^{-1}.

HRMS (ESI-TOF) m/z calcd for $C_{16}H_{22}O_6Na$ $[M+Na]^+$ 333.1314; found 333.1320.

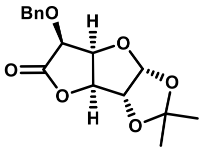

1f

1,2-*O*-Isopropylidene-5-*O*-benzyl-*α*-D-glucofuranosidurono-6,3-lactone (**1f**)

The synthesis of compound **1f** has been previously reported. It was synthesized according to an established protocol.[5] The product was obtained as a yellowish oil (1.36 g, 64%).

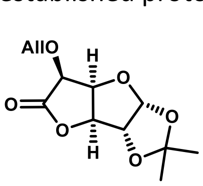

1g

1,2-*O*-Isopropylidene-5-*O*-allyl-*α*-D-glucofuranosidurono-6,3-lactone (**1g**)

The synthesis of compound **1g** has been previously reported. It was synthesized according to an established protocol.[6] The product was obtained as a yellowish amorphous solid (0.55 g, 93%).

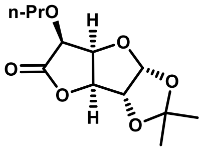

1h

1,2-*O*-Isopropylidene-5-*O*-propyl-*α*-D-glucofuranosidurono-6,3-lactone (**1h**)

A solution of **1g** (128.12 mg, 0.5 mmol) in EtOH (3 mL) was hydrogenolyzed in the presence of 10% Pd-C (30 mg) for 3 h. The reaction mixture was filtered through a pad of Celite and washed with AcOEt (20 mL). The filtrate was concentrated under reduced pressure afforded **1h** (128.5 mg, quant.), without purification as an amorphous white solid.

$[\alpha]^{20}_{D}$: +67.8 (*c* = 1.0, $CHCl_3$).

^{1}H NMR (500 MHz, $CDCl_3$) δ 6.03 (d, *J* = 3.7 Hz, 1H), 4.98 (dd, *J* = 4.2, 2.9 Hz, 1H), 4.79 (d, *J* = 3.7 Hz, 1H), 4.76 (d, *J* = 2.9 Hz, 1H), 4.25 (d, *J* = 4.2 Hz, 1H), 3.82 (dt, *J* = 8.9, 6.8 Hz, 1H), 3.66 (dt, *J* = 8.9, 6.9 Hz, 1H), 1.72 (h, *J* = 7.2 Hz, 2H), 1.52 (s, 3H), 1.34 (s, 3H), 0.96 (t, *J* = 7.4 Hz, 3H).

^{13}C NMR (126 MHz, $CDCl_3$) δ 171.7, 113.1, 107.0, 82.5, 81.7, 77.3, 76.7, 73.5, 26.9, 26.5, 22.7, 10.2.

IR (film) $\tilde{\nu}$: 2966, 2939, 2878, 1801, 1377, 1219, 1139, 1097, 1033, 969 cm^{-1}.

HRMS (ESI-TOF) m/z calcd for $C_{12}H_{18}O_6Na$ $[M+Na]^+$ 281.1001; found 281.1005.

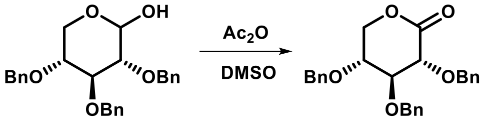

2,3,4-Tri-*O*-benzyl-D-xylono-1,5-lactone (**1i**)

A mixture of acetic anhydride (1.02 mL) and dimethyl sulfoxide (2.2 mL) was added to 2,3,4-tri-*O*-benzyl-D-xylopyranose[7] (450.0 mg, 1.07 mmol) and stirred at room temperature for 16 h under argon atmosphere. Water (5 mL) was added and the product extracted with AcOEt (3 x 10 mL), washed with saturated $NaHCO_3$ and brine, dried (Na_2SO_4), filtered and concentrated in vacuo. Recrystallization from methanol gave the product **1i** as a white solid (217.8 mg, 49%).

Mp. 123-125°C (from methanol)

$[\alpha]^{20}_{D}$: +4.5 (c = 1.0, $CHCl_3$)

^{1}H NMR (500 MHz, $CDCl_3$) δ δ 7.44-7.39 (m, 2H), 7.37-7.25 (m, 13H), 5.03 (d, *J* = 11.6 Hz, 1H), 4.66 (d, *J* = 11.6 Hz, 2H), 4.60-4.54 (m, 2H), 4.52 (d, *J* = 12.0 Hz, 1H), 4.40 (ddd, *J* = 12.3, 3.4, 1.5 Hz, 1H), 4.29 (dd, *J* = 12.3, 2.1 Hz, 1H), 4.14 (d, *J* = 6.6 Hz, 1H), 3.90 (dt, *J* = 6.6, 1.8 Hz, 1H), 3.8 (m, 1H).

^{13}C NMR (126 MHz, $CDCl_3$) δ 169.7, 137.3, 137.1, 137.0, 128.5 (3C), 128.3, 128.1, 128.0 (2C), 127.9, 127.8, 81.3, 78.1, 75.2, 73.3, 72.7, 70.6, 65.7.

IR (film) $\tilde{\nu}$: 3087, 3029, 2913, 2870, 1750, 1496, 1453, 1351, 1256, 1141, 1105, 1074, 1023, 874 cm^{-1}.

HRMS (ESI-TOF) m/z: $[M+Na]^+$ calcd for $C_{26}H_{26}O_5Na$ 441.1678; found 441.1686.

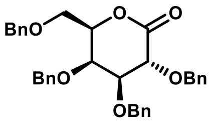

1j

2,3,4,6-Tetra-*O*-benzyl-D-galactono-1,5-lactone (**1j**)

The synthesis of compound **1j** has been previously reported. It was synthesized according to an established protocol.[8] The product was obtained as a yellowish oil (186.7 mg, 69%).

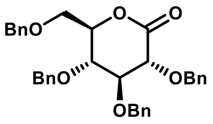

1k

2,3,4,6-Tetra-*O*-benzyl-D-glucono-1,5-lactone (**1k**)

The synthesis of compound **1k** has been previously reported. It was synthesized according to an established protocol.[8] The product was obtained as a yellowish oil (465.5 mg, 86%).

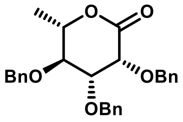

1l

2,3,4-Tri-*O*-benzyl-L-rhamnono-1,5-lactone (**1l**)

The synthesis of compound **1l** has been previously reported. It was synthesized according to an established protocol.[8] The product was obtained as a white solid (502.7 mg, 58%).

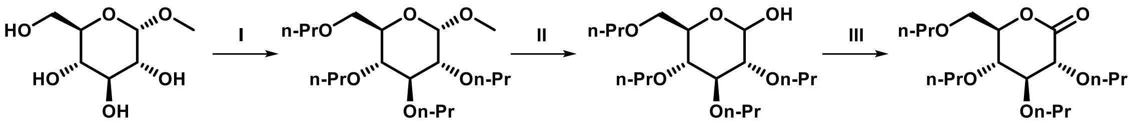

A B C 1m

2,3,4,6-Tetra-*O*-propyl-D-glucono-1,5-lactone (**1m**)

I. To a stirring solution of the compound **A** (1.94 g, 10.0 mmol) in anhydrous DMF (50 mL) was added 60% NaH suspension in mineral oil (2.0 g, 49.7 mmol) under argon atmosphere at 0 °C. After 1 h, 1-Iodopropane (5.4 mL, 55.0 mmol) was added at 0 °C, and the mixture was warmed to ambient temperature and stirred for 16 h. Subsequently, the reaction was quenched by addition of methanol (10 mL), the solvents were removed under reduced pressure and the residue was taken up in H_2O/CH_2Cl_2 (1:1). The product in the aqueous layer was extracted (3 x CH_2Cl_2). The organic phase was washed with brine, dried (Na_2SO_4) and concentrated under reduced pressure. The residue crude mixture was used without further purification in the next step.

II. The residue from the previous step was heated under reflux for 16 h with 1M H_2SO_4 (12.8 mL), AcOH (12.8 mL) and 1.4-dioxane (12.8 mL). The mixture was cooled to room temperature and water was added and extracted 3 x CH_2Cl_2, dried (Na_2SO_4), filtered and concentrated under reduced pressure. The residue crude mixture was used without further purification in the next step.

III. A mixture of acetic anhydride (0.72 mL) and dimethyl sulfoxide (1.5 mL) was added to the residue from the previous step and stirred at room temperature for 16 h. Water (20 mL) was added and the product extracted with AcOEt (3 x 10 mL), washed with saturated $NaHCO_3$ and brine, dried (Na_2SO_4),

filtered and concentrated under reduced pressure. The crude product was purified by column chromatography using 15-30% MTBE/hexanes solvent system to give the title compound **1m** as a yellow oil (198.3 mg, 6% after all reaction steps).

$[\alpha]^{20}_{D}$: +75.9 (c = 1.1, $CHCl_3$)

^{1}H NMR (500 MHz, $CDCl_3$) δ 4.34 (ddd, *J* = 8.8, 3.3, 2.5 Hz, 1H), 3.87 (d, *J* = 6.2 Hz, 1H), 3.82 (dt, *J* = 9.1, 6.6 Hz, 1H), 3.74-3.37 (m, 11H), 1.68-1.55 (m, 8H), 0.96-0.89 (m, 12H).

^{13}C NMR (126 MHz, $CDCl_3$) δ 169.8, 81.8, 78.6, 78.1, 76.5, 73.8, 73.6, 73.4 (2C), 68.9, 23.2 (2C), 22.9, 22.8, 10.6 (3C), 10.4.

IR (film) $\tilde{\nu}$: 2963, 2936, 2876, 1760, 1463, 1377, 1224, 1097, 994, 962 cm^{-1}.

HRMS (ESI-TOF) m/z: $[M+Na]^+$ calcd for $C_{18}H_{34}O_6Na$ 369.2253; found 369.2263.

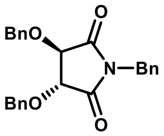

3a

(3*R*,4*R*)-1-Benzyl-3,4-di(benzyloxy)pyrrolidine-2,5-dione (**3a**)

The synthesis of compound **1l** has been previously reported. It was synthesized according to an established protocol.[9] The product was obtained as a yellow oil (474.6 mg, 47%).

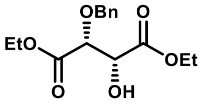

3b

Diethyl 2-*O*-benzyl-L-tartrate (**3b**)

To a solution of diethyl L-(+)-tartrate (1.0 g, 4.85 mmol) in anhydrous dichloromethane (20 mL), silver oxide (1.7 g, 7.27 mmol) and benzyl bromide (634 µl, 5.34 mmol) were added and stirred at room temperature for 8 h under argon atmosphere in the dark. After that the reaction mixture was filtered off through a pad of Celite. The solvent was removed under reduced pressure. The crude product was purified by column chromatography using 15-30% MTBE/hexanes solvent system to give the title compound **3b** as a yellowish oil (564.3 mg, 40%).

$[\alpha]^{25}_{D}$: +80.7 (*c* = 1.0, $CHCl_3$).

^{1}H NMR (400 MHz, $CDCl_3$) δ 7.36-7.24 (m, 5H), 4.86 (d, *J* = 11.8 Hz, 1H), 4.58 (dd, *J* = 8.9, 2.3 Hz, 1H), 4.42 (d, *J* = 11.8 Hz, 1H), 4.34-4.17 (m, 4H), 4.05 (m, 1H), 3.09 (d, *J* = 8.9, 1H, OH), 1.32 (t, *J* = 7.1 Hz, 3H), 1.17 (t, *J* = 7.1 Hz, 3H).

^{13}C NMR (126 MHz, $CDCl_3$) δ 171.1, 169.3, 136.7, 128.4, 128.2, 128.1, 78.1, 72.9, 72.3, 62.0, 61.6, 14.2, 14.0.

IR (film) $\tilde{\nu}$: 3493, 2982, 2937, 2906, 1752, 1455, 1263, 1199, 1139, 1097, 1023 cm^{-1}.

HRMS (ESI-TOF) m/z calcd for $C_{15}H_{20}O_6Na$ $[M+Na]^+$ 319.1158; found 319.1160.

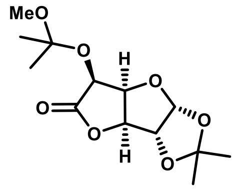

6

1,2-*O*-Isopropylidene-5-*O*-(2-methoxypropan)-*α*-D-glucofuranosidurono-6,3-lactone (**6**)

A mixture of 1,2-*O*-Isopropylidene-*α*-D-glucurono-3,6-lactone[5] (432.38 mg, 2.0 mmol) and 2-metoxypropene (383.0 µL, 4.0 mmol) in dichloromethane (2.0 mL) under argon atmosphere was cooled to 0°C, then pyridinium p-toluenesulfonate (1.0 mg, 4.0 µmol) was added. The cooling bath was removed, and the reaction mixture was stirred at room temperature for 16 h. The reaction mixture was washed with water (2 x 10 mL), saturated aqueous $NaHCO_3$ (2 x 10 mL), and then brine (1 x 10 mL). The organic layer was dried over $MgSO_4$, filtered, and concentrated under reduced pressure. The crude product was purified by column chromatography using 40-50% AcOEt/hexanes solvent system to give the title compound **6** as a white waxy solid (426.2 mg, 74%).

$[\alpha]^{22}_D$: +71.5 (*c* = 1.0, $CHCl_3$).

^{1}H NMR (400 MHz, $CDCl_3$) δ 6.02 (d, *J* = 3.7 Hz, 1H), 4.84 (dd, *J* = 4.3, 2.9 Hz, 1H), 4.79 (d, *J* = 3.7 Hz, 1H), 4.76 (d, *J* = 2.9 Hz, 1H), 4.63 (d, *J* = 4.3 Hz, 1H), 3.35 (s, 3H), 1.51 (s, 3H), 1.48 (s, 3H), 1.47 (s, 3H), 1.34 (s, 3H).

^{13}C NMR (100 MHz, $CDCl_3$) δ 172.5, 113.0, 106.9, 102.4, 82.7, 81.5, 78.6, 68.7, 49.6, 26.9, 26.4, 25.2, 24.4.

IR (film) $\tilde{\nu}$: 2991, 2945, 1801, 1460, 1382, 1216, 1162, 1129, 1100, 1034 cm^{-1}.

HRMS (ESI-TOF) m/z calcd for $C_{13}H_{20}O_7Na$ $[M+Na]^+$ 311.1107; found 311.1108.

3. Photochemical synthesis of carbohydrate-derived 2-deoxylactones

3.1 General procedure for 2-deoxylactones synthesis

The reactions were prepared in glove box. Substrates **1a-m** or **3a-3b** (0.144 mmol) were added to a quartz vial equipped with a magnetic stir bar and dissolved in degassed cyclohexane (16.0 mL). If a substrate showed limited solubility, the mixture was subjected to sonication for a few minutes. Then the vial was sealed with a septum, transferred into a photoreactor and irradiated with eight UVC lamps (9W, 254 nm) at internal temperature 25-30 °C (RT) until complete conversion of substrate (TLC). Upon completion of the reaction, the reaction mixture was concentrated under reduced pressure. The products **2a-m** and **4a-4b** were purified by column chromatography using appropriate solving system.

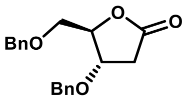

2a

3,5-Di-*O*-benzyl-2-deoxy-D-*erythro*-pentafurano-1,4-lactone (**2a**)

The compound was synthesized according to the general procedure starting with **1a** or **1b** (60.26 mg, 0.144 mmol) in cyclohexane (16.0 mL) for 1 h 45 min. The crude product was purified by column chromatography using 15-30% MTBE/hexanes solvent system to give the title compound **2a** as a yellowish oil (28.6 mg, 64% for 2,3,5-Tri-*O*-benzyl-D-ribono-1,4-lactone (**1a**)) and (29.1 mg, 65% for 2,3,5-Tri-*O*-benzyl-D-arabino-1,4-lactone (**1b**).

$[\alpha]^{20}_D$: +27.0 (c = 1.0, $CHCl_3$).

^{1}H NMR (500 MHz, $CDCl_3$) δ 7.39-7.21 (m, 10H), 4.62 (m, 1H), 4.58-4.52 (m, 2H), 4.51-4.46 (m, 2H), 4.28 (ddd, J = 6.9, 2.1, 2.0 Hz, 1H), 3.67 (dd, J = 10.8, 3.3 Hz, 1H), 3.62 (dd, J = 10.8, 2.9 Hz, 1H), 2.86 (dd, J = 18.0, 6.9 Hz, 1H), 2.57 (dd, J = 18.1, 2.3 Hz, 1H).

^{13}C NMR (126 MHz, $CDCl_3$) δ 175.5, 137.3, 137.0, 128.6, 128.5, 128.0, 127.9, 127.7, 127.6, 84.0, 76.0, 73.7, 71.2, 69.6, 35.7.

IR (film) $\tilde{\nu}$: 3031, 2924, 2861, 1781, 1453, 1361, 1170, 1097, 1028 cm^{-1}.

HRMS (ESI-TOF) m/z calcd for $C_{19}H_{20}O_4Na$ $[M+Na]^+$ 335.1259; found 335.1263.

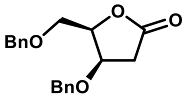

2c

3,5-Di-*O*-benzyl-2-deoxy-D-*threo*-pentafurano-1,4-lactone (**2c**)

The compound was synthesized according to the general procedure starting with **1c** (60.26 mg, 0.144 mmol) in cyclohexane (16.0 mL) for 1 h 45 min. The crude product was purified by column chromatography using 15-50% MTBE/hexanes solvent system to give the title compound **2c** as a yellowish oil (24.0 mg, 53%).

$[\alpha]^{20}_D$: -6.8 (c = 1.0, $CHCl_3$).

^{1}H NMR (500 MHz, $CDCl_3$) δ 7.37-7.23 (m, 10H), 4.66-4.54 (m, 4H), 4.46 (d, J = 11.9 Hz, 1H), 4.34 (m, 1H), 3.91-3.82 (m, 2H), 2.72 (dd, J = 17.5, 3.0 Hz, 1H), 2.63 (dd, J = 17.5, 6.0 Hz, 1H).

^{13}**C NMR** (126 MHz, $CDCl_3$) δ 174.6, 137.7, 137.1, 128.5, 128.4, 128.1, 127.8, 127.7, 127.6, 81.9, 74.4, 73.7, 71.7, 67.6, 35.5.

IR (film) $\tilde{\nu}$: 3031, 2925, 2866, 1784, 1453, 1160, 1130, 1097, 1063, 1028 cm^{-1}.

HRMS (ESI-TOF) m/z calcd for $C_{19}H_{20}O_4Na$ $[M+Na]^+$ 335.1259; found 335.1258.

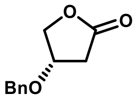

2d

3-*O*-Benzyl-D-*glycero*-pentafurano-1,4-lactone (**2d**)

The compound was synthesized according to the general procedure starting with **1d** (43.0 mg, 0.144 mmol) in cyclohexane (16.0 mL) for 1 h 45 min. The crude product was purified by column chromatography using 15-50% AcOEt/hexanes solvent system to give the title compound **2d** as a yellowish oil (19.4 mg, 70%).

$[\alpha]^{25}_D$: -26.6 (*c* = 1.0, $CHCl_3$).

^{1}**H NMR** (500 MHz, $CDCl_3$) δ 7.40-7.27 (m, 5H), 4.56 (d, *J* = 11.8 Hz, 1H), 4.52 (d, *J* = 11.8 Hz, 1H), 4.42-4.32 (m, 3H), 2.72-2.60 (m, 2H).

^{13}**C NMR** (126 MHz, $CDCl_3$) δ 175.3, 136.9, 128.6, 128.2, 127.7, 73.8, 73.0, 71.2, 34.9.

IR (film) $\tilde{\nu}$: 3031, 2925, 2859, 1780, 1454, 1375, 1168, 1093, 1041, 1000 cm^{-1}.

HRMS (EI) m/z calc for $C_{11}H_{12}O_3$ $[M]^+$ 192.0786, found: 192.0788.

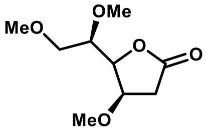

2e

2-Deoxy-3,5,6-tri-*O*-methyl-D-*xylo*-hexafurano-1,4-lactone (**2e**)

The compound was synthesized according to the general procedure starting with **1e** (44.7 mg, 0.144 mmol) in cyclohexane (16.0 mL) for 2 h 55 min. The crude product was purified by column chromatography using 15-50% AcOEt/hexanes solvent system to give the title compound **2e** as an amorphous white solid (23.5 mg, 80%).

$[\alpha]^{20}_D$: +0.3 (*c* = 1.0, $CHCl_3$).

^{1}**H NMR** (500 MHz, $CDCl_3$) δ 4.44 (dd, *J* = 9.3, 3.8 Hz, 1H), 4.10 (ddd, *J* = 4.8, 3.8, 0.9 Hz, 1H), 3.77 (dd, *J* = 10.7, 2.2 Hz, 1H), 3.71 (ddd, *J* = 9.3, 3.6, 2.2 Hz, 1H), 3.51 (dd, *J* = 10.7, 3.6 Hz, 1H), 3.46 (s, 3H), 3.40 (s, 3H), 3.36 (s, 3H), 2.70 (dd, *J* = 17.5, 0.9 Hz, 1H), 2.59 (dd, *J* = 17.5, 4.8 Hz, 1H).

^{13}**C NMR** (126 MHz, $CDCl_3$) δ 174.8, 80.6, 76.5, 75.9, 70.6, 59.4, 57.9, 56.8, 35.3.

IR (film) $\tilde{\nu}$: 2982, 2930, 2832, 1787, 1453, 1360, 1215, 1125, 1104, 1065, 1030, 1012, 906 cm^{-1}.

HRMS (ESI-TOF) m/z calcd for $C_9H_{16}O_5Na$ $[M+Na]^+$ 227.0895; found 227.0898.

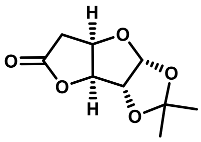

2f

1,2-*O*-Isopropylidene-5-deoxy-*α*-D-glucofuranosidurono-6,3-lactone (**2f**)

The compound was synthesized according to the general procedure starting with **1f** (44.1 mg, 0.144 mmol) or **1g** (36.9 mg, 0.144 mmol) or **1h** (37.2 mg, 0.144 mmol) in cyclohexane (16.0 mL) for 4 h 30 min. The crude product was purified by column chromatography using 15-50% AcOEt/hexanes solvent system to give the title compound **2f** as an amorphous white solid (25.3 mg, 88% for **1f**, 16.9 mg, 59% for **1g**, and 15.7 mg, 55% for **1h**).

$[\alpha]^{25}_{D}$: +74.8 (*c* = 1.0, $CHCl_3$).

^{1}H NMR (400 MHz, $CDCl_3$) δ 5.96 (d, *J* = 3.8 Hz, 1H), 4.99 (m, 1H), 4.84 (d, *J* = 3.8 Hz, 1H), 4.81 (d, *J* = 3.4 Hz, 1H), 2.75 (d, *J* = 18.1 Hz, 1H), 2.68 (dd, *J* = 18.2, 4.1 Hz, 1H), 1.51 (s, 3H), 1.34 (s, 3H).

^{13}C NMR (150 MHz, $CDCl_3$) δ 174.1, 112.7, 106.2, 85.5, 82.5, 78.0, 35.9, 26.9, 26.5.

IR (film) $\tilde{\nu}$: 2999, 2980, 2937, 2853, 1788, 1378, 1265, 1189, 1164, 1144, 1056, 1007, 897 cm^{-1}.

HRMS (EI) m/z calc for $C_9H_{12}O_5$ $[M]^+$ 200.0685, found: 200.0684.

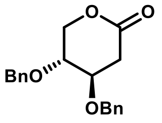

2i

3,4-Di-*O*-benzyl-2,5-dideoxy-L-*threo*-hexapyrano-1,5-lactone (**2i**)

The compound was synthesized according to the general procedure starting with **1i** (60.26 mg, 0.144 mmol) in cyclohexane (16.0 mL) for 1 h 45 min. The crude product was purified by column chromatography using 15-30% MTBE/hexanes solvent system to give the title compound **2i** as a yellowish oil (23.6 mg, 52%).

$[\alpha]^{20}_{D}$: -43.0 (*c* = 0.6, $CHCl_3$).

^{1}H NMR (500 MHz, $CDCl_3$) δ 7.31-7.17 (m, 10H), 4.58 (d, *J* = 11.8 Hz, 1H), 4.54 (d, *J* = 11.8 Hz, 1H), 4.50-4.41 (m, 3H), 4.31 (ddd, *J* = 12.3, 3.0, 1.2 Hz, 1H), 3.88 (m, 1H), 3.66 (m, 1H), 2.85 (dd, *J* = 17.5, 4.8 Hz, 1H), 2.65 (dd, *J* = 17.4, 3.4 Hz, 1H).

^{13}C NMR (126 MHz, $CDCl_3$) δ 169.1, 137.2, 137.1, 128.6 (2C), 128.1 (2C), 127.7, 127.6, 72.9, 72.0, 71.2 (2C), 67.2, 33.1.

IR (film) $\tilde{\nu}$: 3062, 3031, 2900, 2871, 1737, 1496, 1453, 1359, 1226, 1171, 1095, 1071, 930 cm^{-1}.

HRMS (ESI-TOF) m/z calcd for $C_{19}H_{20}O_4Na$ $[M+Na]^+$ 335.1259; found 335.1267.

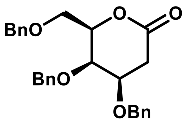

2j

3,4,6-Tri-*O*-benzyl-2-deoxy-D-lyxo-hexono-1,5-lactone (**2j**)

The compound was synthesized according to the general procedure starting with **1j** (77.56 mg, 0.144 mmol) in cyclohexane (16.0 mL) for 1 h 45 min. The crude product was purified by column chromatography using 15-50% MTBE/hexanes solvent system to give the title compound **2j** as a yellowish oil (31.4 mg, 50%).

$[\alpha]^{25}_D$: +8.3 (c = 1.0, DCM).

^{1}H NMR (500 MHz, $CDCl_3$) δ 7.40-7.26 (m, 15H), 4.96 (d, *J* = 11.4 Hz, 1H), 4.68-4.45 (m, 5H), 4.34 (ddd, *J* = 7.7, 5.5, 1.7 Hz, 1H), 4.19 (m, 1H), 3.88 (ddd, *J* = 11.2, 6.6, 1.9 Hz, 1H), 3.77 (m, 1H), 3.67 (m, 1H), 2.98 (dd, *J* = 17.6, 11.2 Hz, 1H), 2.90 (dd, *J* = 17.6, 6.5 Hz, 1H).

^{13}C NMR (126 MHz, $CDCl_3$) δ 168.9, 138.0, 137.5, 137.4, 128.6, 128.5, 128.3, 128.0, 127.9 (2C), 127.8 (2C), 127.5, 78.2, 74.4, 74.3, 73.7, 70.7, 70.4, 67.9, 33.0.

IR (film) $\tilde{\nu}$: 3061, 3030, 2924, 2869, 1739, 1453, 1362, 1229, 1162, 1100, 1062, 1026 cm^{-1}.

HRMS (ESI-TOF) m/z calcd for $C_{27}H_{28}O_5Na$ $[M+Na]^+$ 455.1834; found 455.1837.

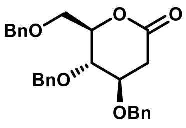

2k

3,4,6-Tri-*O*-benzyl-2-deoxy-D-arabino-hexono-1,5-lactone (**2k**)

The compound was synthesized according to the general procedure starting with **1k** (77.56 mg, 0.144 mmol) in cyclohexane (16.0 mL) for 1 h 45 min. The crude product was purified by column chromatography using 15-30% MTBE/hexanes solvent system to give the title compound **2k** as a yellowish oil (22.41 mg, 36%).

$[\alpha]^{25}_D$: +35.6 (c = 0.4, DCM)

^{1}H NMR (600 MHz, $CDCl_3$) δ 7.36-28 (m, 13H), 7.23-7.20 (m, 2H), 6.65-4.49 (m, 6H), 4.30 (ddd, *J* = 7.6, 3.9, 3.9 Hz, 1H), 3.95 (m, 1H), 3.89 (m, 1H), 3.75-3.69 (m, 2H), 2.85 (dd, *J* = 16.5, 4.5 Hz, 1H), 2.76 (dd, J = 16.5, 5.3 Hz, 1H).

^{13}C NMR (150 MHz, $CDCl_3$) δ 169.4, 137.7, 137.3 (2C), 128.5 (2C), 128.4, 128.2, 128.1, 128.0 (2C), 127.8 (2C), 79.3, 75.1, 74.7, 73.5, 72.9, 71.1, 68.8, 33.8.

IR (film) $\tilde{\nu}$: 3087, 3031, 2925, 2856, 1754, 1496, 1453, 1234, 1094, 1074, 1028 cm^{-1}.

HRMS (ESI-TOF) m/z calcd for $C_{27}H_{28}O_5Na$ $[M+Na]^+$ 455.1834; found 455.1836.

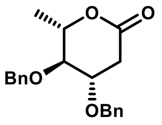

2l

3,4-Di-*O*-benzyl-2,6-dideoxy-L-arabino-hexono-1,5-lactone (**2l**)

The compound was synthesized according to the general procedure starting with **1l** (62.20 mg, 0.144 mmol) in cyclohexane (16.0 mL) for 4 h 30 min. The crude product was purified by column chromatography using 15-50% MTBE/hexanes solvent system to give the title compound **2l** as a colourless oil (17.4 mg, 37%).

$[\alpha]^{20}_{D}$: -23.1 (c = 0.9, $CHCl_3$).

^{1}H NMR (500 MHz, $CDCl_3$) δ 7.39-7.29 (m, 10H), 6.67-4.62 (m, 2H), 4.53 (d, *J* = 11.4 Hz, 1H), 4.49 (d, *J* = 11.9 Hz, 1H), 4.21 (dq, *J* = 8.2, 6.3 Hz, 1H), 3.96 (q, *J* = 4.3 Hz, 1H), 3.48 (dd, *J* = 8.2, 3.5 Hz, 1H), 2.83-2.79 (m, 2H), 1.44 (d, *J* = 6.3 Hz, 3H).

^{13}C NMR (126 MHz, $CDCl_3$) δ 170.0, 137.2 (2C), 128.6, 128.5, 128.2, 128.1, 128.0, 127.9, 81.2, 75.7, 75.1, 72.8, 70.9, 33.6, 18.8.

IR (film) $\tilde{\nu}$: 3030, 2925, 2852, 1758, 1496, 1454, 1382, 1243, 1094, 1069, 1028, 1000 cm^{-1}.

HRMS (ESI-TOF) m/z calcd for $C_{20}H_{22}O_4Na$ $[M+Na]^+$ 349.1416; found 349.1424.

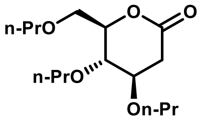

2m

2-Deoxy-3,4,6-tri-*O*-n-propyl-D-arabino-hexono-1,5-lactone (**2m**)

The compound was synthesized according to the general procedure starting with **1m** (50.0 mg, 0.144 mmol) in cyclohexane (16.0 mL) for 1 h. The crude product was purified by column chromatography using 15-50% MTBE/hexanes solvent system to give the title compound **2m** as a colourless oil (8.9 mg, 21%).

$[\alpha]^{20}_{D}$: +59.6 (c = 0.6, $CHCl_3$).

^{1}H NMR (500 MHz, $CDCl_3$) δ 4.21 (ddd, *J* = 7.8, 4.1, 4.1 Hz, 1H), 3.76 (m, 1H), 3.71-3.67 (m, 2H), 3.66-3.3.59 (m, 2H), 3.53-3.37 (m, 5H), 2.80 (dd, *J* = 16.4, 4.6 Hz, 1H), 2.68 (dd, *J* = 16.5, 5.1 Hz, 1H), 1.65-1.53 (m, 6H), 0.99-0.86 (m, 9H).

^{13}C NMR (126 MHz, $CDCl_3$) δ 169.8, 79.6, 75.7, 75.6, 73.4, 72.6, 71.0, 69.7, 33.8, 23.1, 23.0, 22.8, 10.6, 10.6, 10.5.

IR (film) $\tilde{\nu}$: 2963, 2934, 2876, 1758, 1462, 1341, 1232, 1101, 997 cm^{-1}.

HRMS (ESI-TOF) m/z calcd for $C_{15}H_{28}O_5Na$ $[M+Na]^+$ 311.1834; found 311.1840.

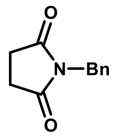

4a

1-Benzyl-pyrrolidine-2,5-dione (**4a**)

The compound was synthesized according to the general procedure starting with **3a** (57.81 mg, 0.144 mmol) in cyclohexane (16.0 mL) for 2 h 30 min. The crude product was purified by column chromatography using 15-50% MTBE/hexanes solvent system to give the title compound **4a** as a white waxy solid (23.9 mg, 88%).

^{1}H NMR (600 MHz, $CDCl_3$) δ 7.41-7.36 (m, 2H), 7.32-7.24 (m, 3H), 4.65 (s, 2H), 2.69 (s, 4H).

^{13}C NMR (150 MHz, $CDCl_3$) δ 176.8, 135.8, 128.9, 128.6, 128.0, 42.4, 28.2.

IR (film) $\tilde{\nu}$: 3034, 2938, 1699, 1400, 1344, 1166, 1082 cm^{-1}.

HRMS (APCI-TOF) m/z calcd for $C_{11}H_{12}NO_2$ $[M+H]^+$ 190.0868; found 190.0869.

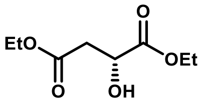

4b

Diethyl (*R*)-(+)-malate (**4b**)

The compound was synthesized according to the general procedure starting with **3b** (42.67 mg, 0.144 mmol) in cyclohexane (16.0 mL) for 7 h. The crude product was purified by column chromatography using 15-50% MTBE/hexanes solvent system to give the title compound **4b** as a colourless oil (10.0 mg, 37%).

$[\alpha]^{20}_{D}$: +13.1 (c = 0.4, $CHCl_3$).

^{1}H NMR (500 MHz, $CDCl_3$) δ 4.48 (m, 1H), 4.31-4.24 (m, 2H), 4.18 (q, *J* = 7.1 Hz, 2H), 3.19 (bs, 1H, OH), 2.85 (dd, *J* = 16.3, 4.4 Hz, 1H), 2.78 (dd, *J* = 16.3, 6.0 Hz, 1H), 1.30 (t, *J* = 7.1 Hz, 3H), 1.27 (t, *J* = 7.1 Hz, 3H).

^{13}C NMR (126 MHz, $CDCl_3$) δ 173.4, 170.5, 67.3, 62.0, 61.0, 38.7, 14.1 (2C).

IR (film) $\tilde{\nu}$: 3476, 2983, 2932, 2854, 1738, 1447, 1373, 1269, 1216, 1183, 1105, 1026 cm^{-1}.

HRMS (ESI-TOF) m/z calcd for $C_8H_{14}O_5Na$ $[M+Na]^+$ 213.0739; found 213.0744.

3.2 Cyclohexyl(phenyl)methanol (11)

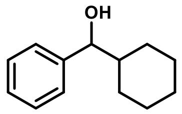

11

Cyclohexyl(phenyl)methanol (**11**)

Under standard conditions of the debenzyloxylation reaction of carbohydrate-derived lactones, cyclohexyl(phenyl)methanol (**11**) was observed as a side product (photochemical reaction between benzaldehyde and cyclohexane). The side product was purified by column chromatography using 15-20% MTBE/hexanes solvent system to give the title compound **11** as a colourless oil.

^{1}H NMR (600 MHz, $CDCl_3$) δ 7.36-7.26 (m, 5H), 4.37 (d, J = 7.2 Hz, 1H), 1.99 (m, 1H), 1.82-1.74 (m, 2H), 1.70-1.58 (m, 3H), 1.38 (m, 1H), 1.27-1.01 (m, 5H), 0.94 (m, 1H).

^{13}C NMR (150 MHz, $CDCl_3$) δ 143.6, 128.2, 127.4, 126.6, 79.4, 45.0, 29.3, 28.8, 26.4, 26.1, 26.0.

The spectroscopic data are in agreement with those reported.[10]

3.3 Procedure for 1.0 mmol scale reaction

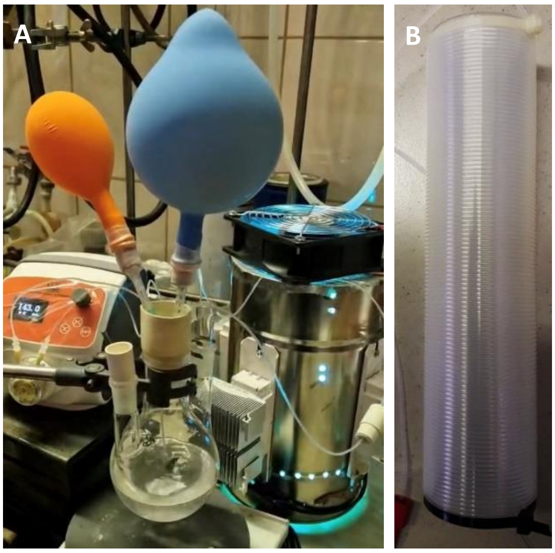

Figure 1. A. Reaction in continuous flow system; B. UV-transparent FEP tube.

Continuous flow system: Reaction on 1.0 mmol scale was performed in continues flow closed system under an argon atmosphere in self-made flow set consisting of: Rayonet-type, self-made photoreactor (with four UV-C lamps Osram, Puritec, HNS, S 9W, λ_{max} = 254 nm), UV-transparent FEP tube of dimensions 0.7 mm i.d. × 1.1 mm o.d, wound on a quartz tube of dimensions 18 cm in length and 4 cm in diameter (loop length 7.0 m, capacity 3.5 mL, number of coils 55), peristaltic pump (model LLG-uniPERIPUMP 1), magnetic stirrer.

Reaction procedure: Substrate **1a** (418.49 mg, 1.0 mmol) was dissolved in degassed cyclohexane (111.0 mL) in the 250 mL flask equipped with a magnetic stir bar. The flask was connected to the flow set and pumping of the solution was starting (flow rate: 6 mL/1 min). The progress of the reaction was monitored by TLC. After the complete conversion the reaction was stopped by turning off the lamps. The reaction mixture was collected in a flask, the flow set was rinsed with 15.0 mL of acetonitrile and 15.0 mL of ethyl acetate, both solutions were combined and concentrated. The crude product was

purified by column chromatography using 30-50% MTBE/hexanes solvent system to give the main product **2a** as a yellowish oil (241.8 mg, 77%).

4. Direct synthesis of 2-deoxy sugar

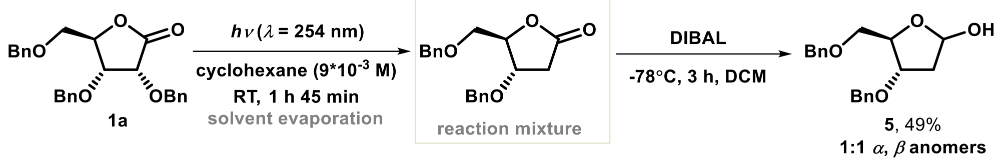

3,5-di-*O*-benzyl-2-deoxy-D-ribofuranose (**5**)

The compound **1a** was dealkyloxylated according to the general procedure, starting with **1a** (60.26 mg, 0.144 mmol) in degassed cyclohexane (16.0 mL) and irradiated for 1 h 45 min. The solvent was then removed under reduced pressure, and the reaction mixture was used directly in the next step without further purification. The reaction mixture was dissolved in dry dichloromethane (1.5 mL) under argon atmosphere, cooled to -78°C and DIBAL (1.0 M in hexane, 0.166 mL, 0.166 mmol) was dropped. After 3 hours at -78°C, the reaction was quenched with a saturated solution of Na_2SO_4, warmed to room temperature, and stirred for 30 minutes. Then the reaction mixture was filtered through the Celite and extracted (3 x CH_2Cl_2), dried over Na_2SO_4, filtered and evaporated under reduced pressure. The crude product was purified by column chromatography using 30-50% AcOEt/hexanes solvent system to give the title compound **5** as a yellowish oil (22.2 mg, 49%, *α*+*β* anomers).

^{1}H NMR (600 MHz, $CDCl_3$) δ 7.38-7.27 (m, 19.4H), 5.57-5.52 (m, 0.76H), 5.50-5.45 (m, 1H), 4.62-4.43 (m, 8.85H), 4.29-4.25 (m, 1.51H), 4.13-4.10 (m, 1H), 3.73 (d, *J* = 8.5 Hz, 0.76H), 3.66-3.61 (m, 1.88H), 3.56-3.50 (m, 1.88H), 3.37 (dd, *J* = 10.1, 5.6 Hz, 1H), 2.23-2.20 (m, 1H), 2.14-2.10 (m, 1.88H).

^{13}C NMR (150 MHz, $CDCl_3$) δ 137.9, 137.8, 137.4, 137.1, 128.5 (2C), 128.4 (2C), 128.0, 127.9, 127.8 (2C), 127.7 (2C), 127.6, 99.4, 83.2, 82.5, 80.1, 79.7, 73.7, 73.4, 71.4, 71.3 (2C), 70.4, 41.8, 39.1.

IR (film) $\tilde{\nu}$: 3418, 3062, 3030, 2924, 2861, 1496, 1453, 1364, 1205, 1093, 1027, 967 cm^{-1}.

HRMS (ESI-TOF) m/z calcd for $C_{19}H_{22}O_4Na$ $[M+Na]^+$ 337.1416; found 337.1418.

5. Mechanistic studies and control experiments

5.1 UV-VIS absorption spectrum

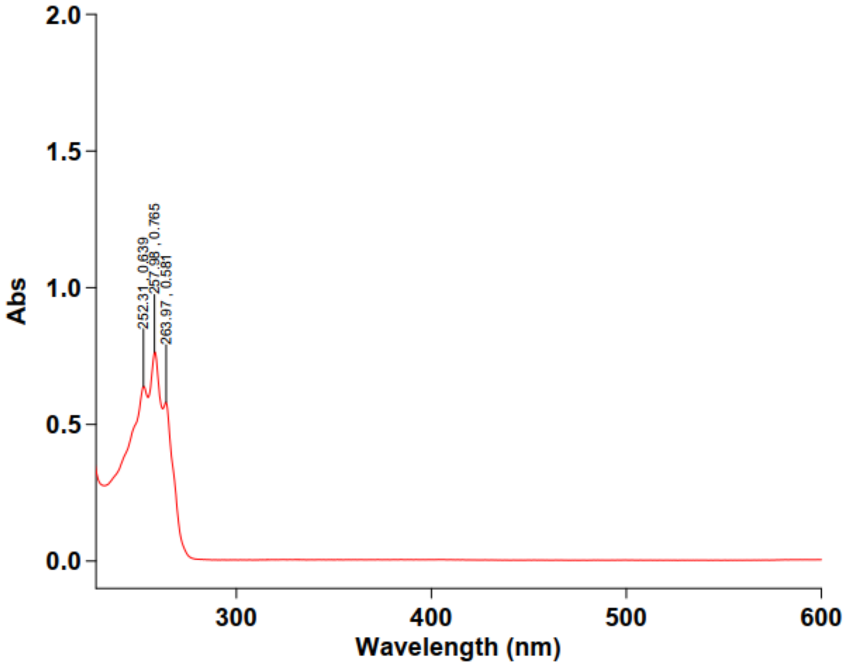

Figure 2. UV-VIS absorption spectrum of **1a** in cyclohexane.

5.2 Control experiments

5.2.1 Reaction was performed in non-degassed conditions

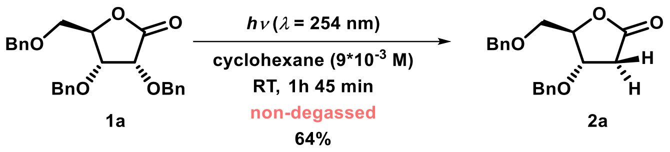

The reaction was prepared under air atmosphere. Substrate **1a** (0.144 mmol) was added to a quartz vial equipped with a magnetic stir bar and dissolved in non-degassed cyclohexane (16.0 mL). The vial was then sealed with a septum, transferred to a photoreactor, and irradiated at room temperature for 1 h 45 min using eight UVC lamps (9W, 254 nm). Upon completion of the reaction, the reaction mixture was concentrated under reduced pressure. The crude product was purified by column chromatography using 30-50% MTBE/hexanes solvent system to give the pure product **2a** (28.6 mg, 64%). The absence of any yield loss suggested that molecular oxygen is not involved in this reaction.

5.2.2 Reaction was performed with triplet quencher – isoprene

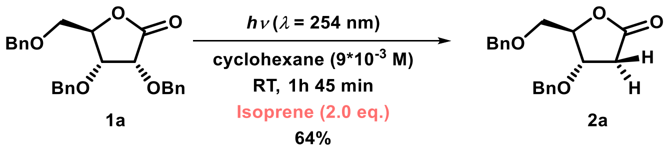

The reaction was conducted under standard conditions with the addition of isoprene. Substrate **1a** (0.144 mmol) was added to a quartz vial equipped with a magnetic stir bar and dissolved in degassed cyclohexane (16.0 mL), followed by the addition of isoprene (28.8 μL, 0.288 mmol). The vial was then sealed with a septum, transferred to a photoreactor, and irradiated at room temperature for 1 hour

and 45 minutes using eight UVC lamps (9 W, 254 nm). Upon completion, the reaction mixture was concentrated under reduced pressure. The crude product was purified by column chromatography using a 30–50% MTBE/hexanes solvent system, yielding the pure product **2a** (28.7 mg, 64%). The observed lack of sensitivity to isoprene suggests that the intermediates involved in the reaction may be singlet species.

5.2.3 Reaction was performed with compound without hydrogens in the gamma position relative to the carbonyl group

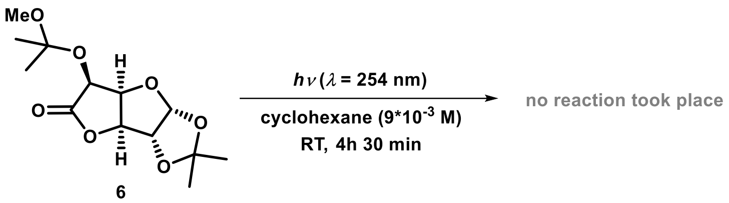

6

The reaction was carried out under standard conditions using compound **6** as the starting substrate. No reaction occurred, and the 2-debenzyloxylated product **2f** was not observed. This suggests that the protons at the gamma positions relative to the carbonyl group are crucial for the reaction to proceed.

5.2.4 ^{1}H NMR spectra of the reaction mixtures

I. The reaction was performed according to the general procedure starting with **1a**. Upon completion of the reaction, the solvent was evaporated and a ^{1}H NMR spectrum of the crude reaction mixture was acquired. The NMR spectra of the reaction mixture showed the presence of 2-deoxy lactone **2a** with side product **11** along with unidentified degradation products.

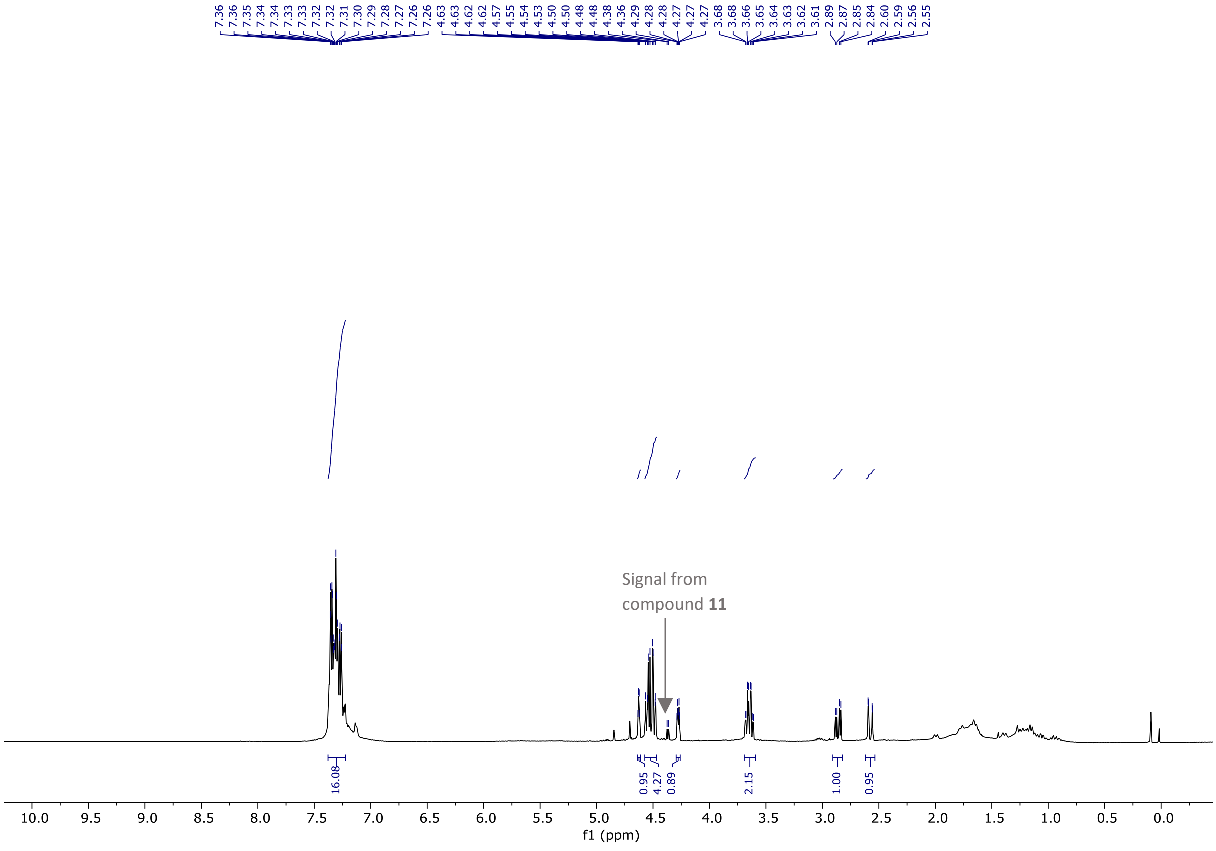

Figure 3. ^{1}H NMR spectrum of the crude product from the model reaction, $CDCl_3$, 500 MHz.

II. The reaction was performed under standard conditions in an NMR quartz tube, with cyclohexane replaced by acetonitrile-D_3 starting with **1a**. To exclude the formation of volatile byproducts, a ^{1}H NMR spectrum was acquired immediately upon completion of the reaction, without solvent evaporation. The NMR spectrum of the reaction mixture showed the presence of 2-deoxy lactone **2a** along with unidentified degradation products.

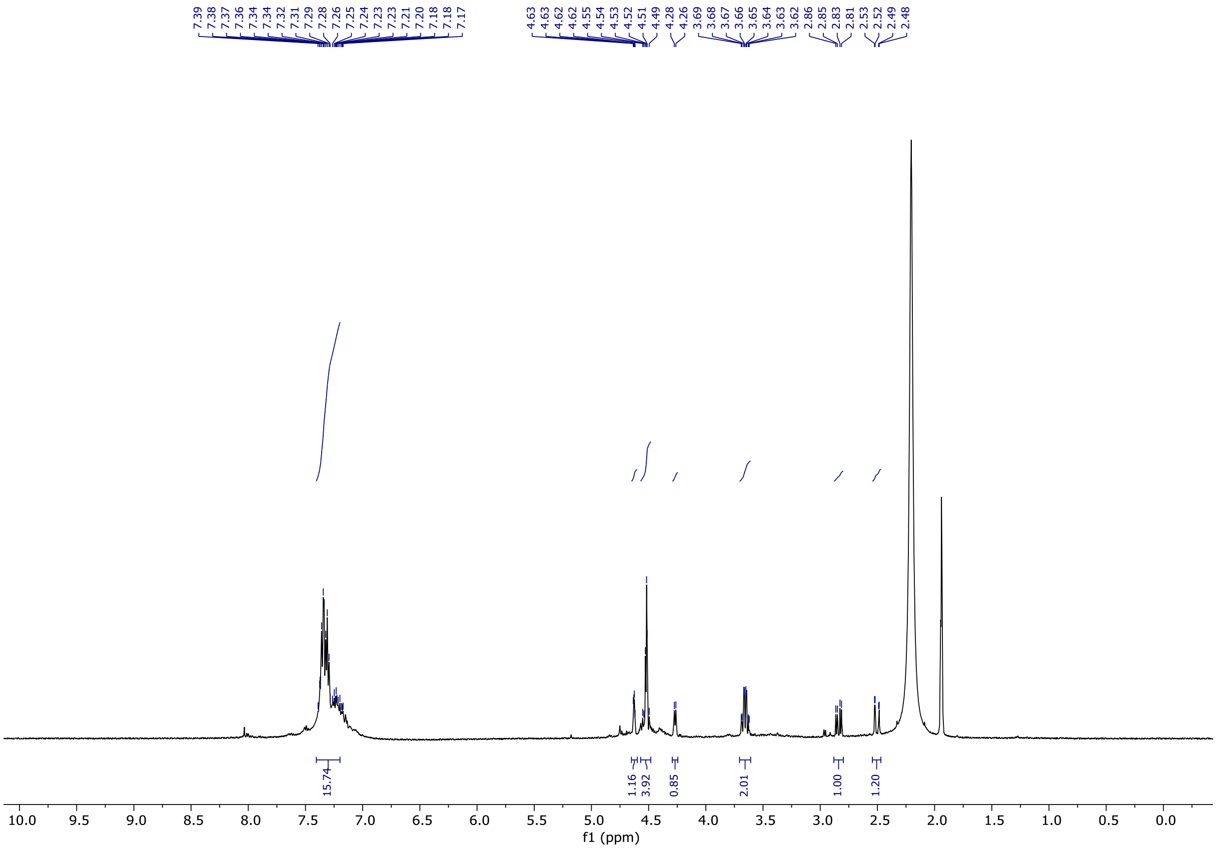

Figure 4. ^{1}H NMR spectrum of the crude product from the model reaction, CD_3CN, 500 MHz.

III. The reaction was performed according to the general procedure starting with **1k**. Upon completion of the reaction, the solvent was evaporated and a ^{1}H NMR spectrum of the crude reaction mixture was acquired. The NMR spectrum of the reaction mixture showed the presence of 2-deoxy lactone **2k** with side product **11** along with unidentified degradation products.

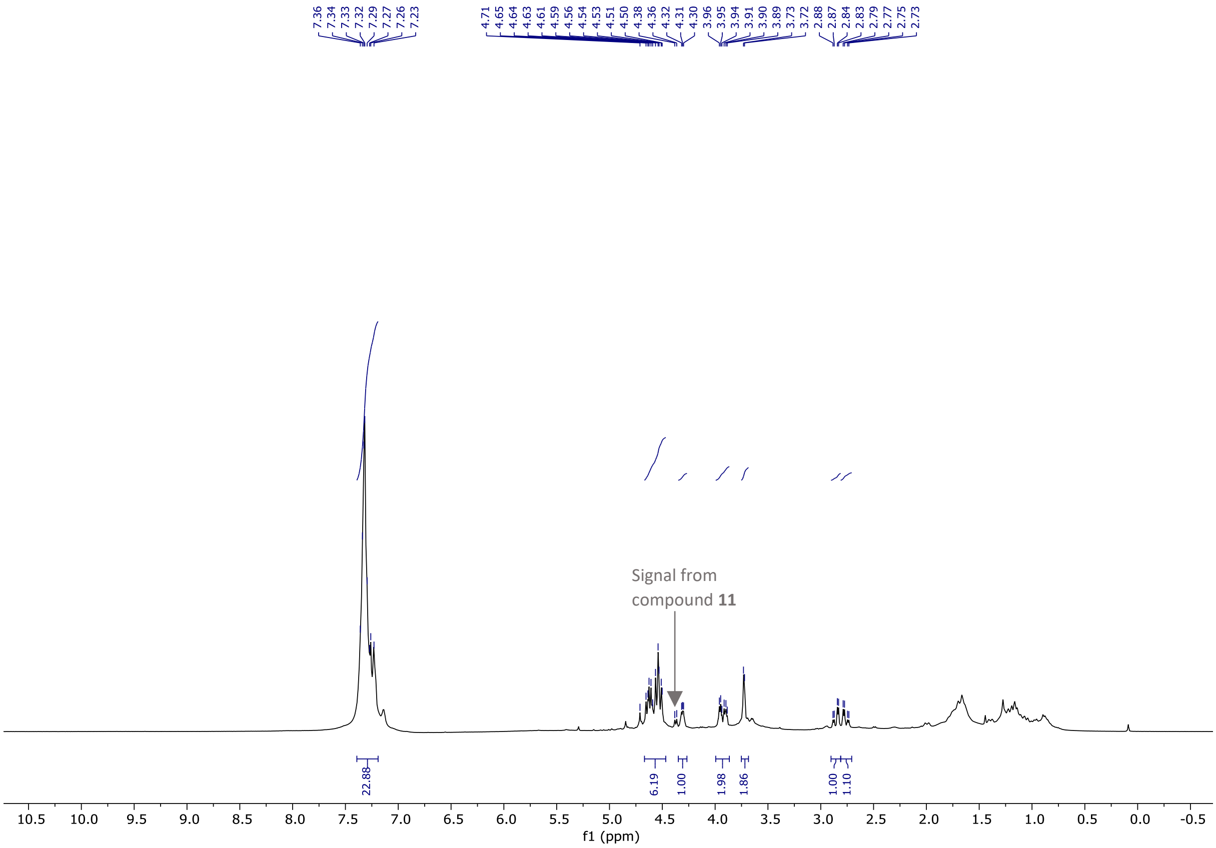

Figure 5. ^{1}H NMR spectrum of the crude product from the reaction with **1k** as a starting material, $CDCl_3$, 400 MHz.

5.2.5 Irradiation of 2a with UVC light

The stability of compound **2a** under UVC irradiation was evaluated by exposing it to UVC light for 2 hours. Subsequent ^{1}H NMR analysis showed no signs of decomposition, confirming its stability under the conditions tested.

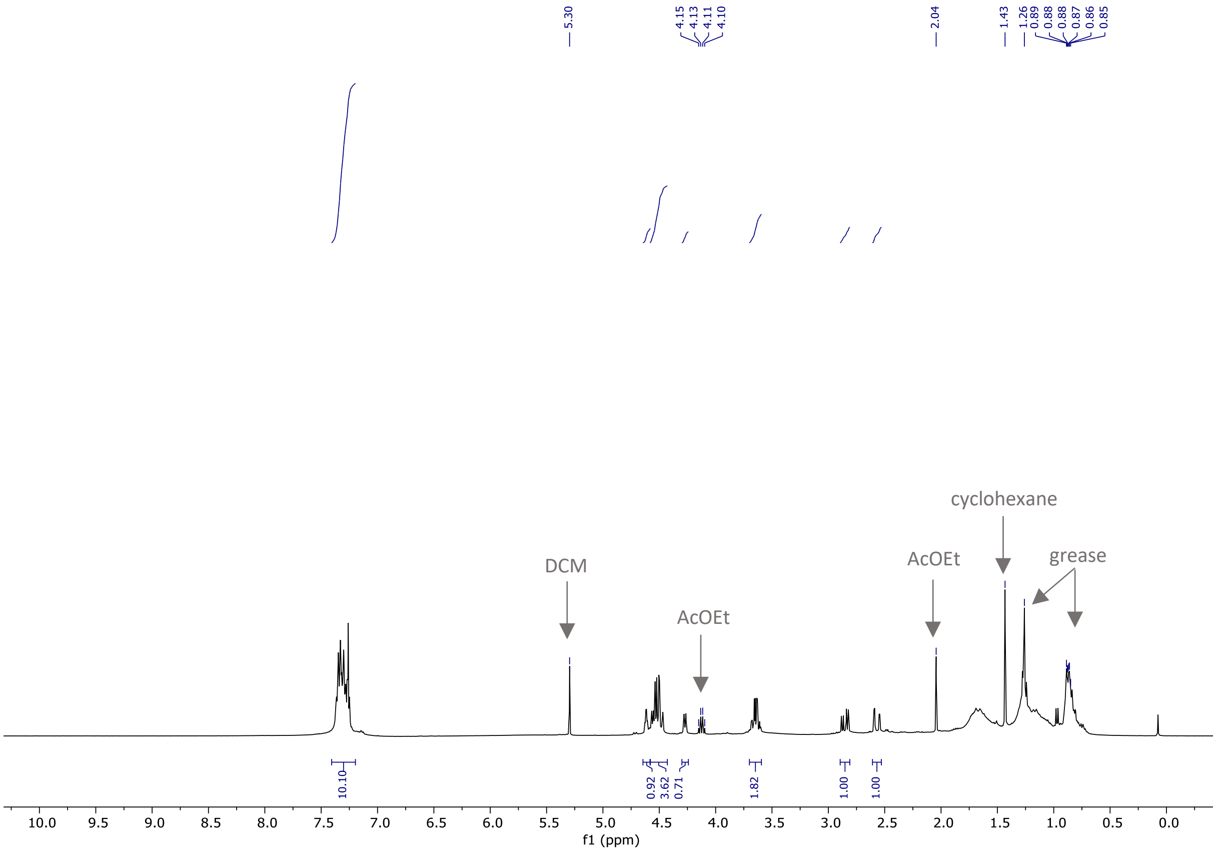

Figure 6. ^{1}H NMR spectrum of the compound **2a** after irradiation in UVC light, $CDCl_3$, 400 MHz.

5.3 Deuterium-labelling experiments

5.3.1 Reaction was performed in CD_3CN

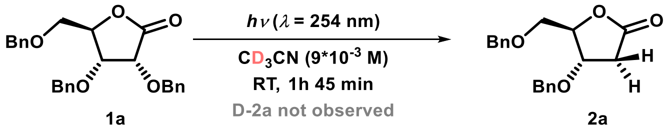

The reaction was performed under standard conditions, cyclohexane was replaced with acetonitrile-D3. The absence of deuterium incorporation into the terminal product **2a** suggests that the *alpha*-hydrogen atom of the 2-deoxylactone product **2a** did not originate from the solvent.

5.3.2 Reaction was performed using deuterium-labelled benzyl group in starting lactone

Procedure for synthesis of D_2-1f

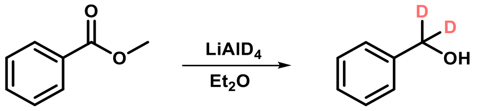

To a mixture of $LiAlD_4$ (12.0 mmol) in dry diethyl ether (40 mL), under argon atmosphere at 0 °C, was added dropwise a solution of methyl benzoate (20.0 mmol) in dry diethyl ether (10 mL). The mixture was stirred at room temperature for 3 h. The reaction was quenched at 0 °C by adding 0.5 mL of H_2O, 0.5 mL of 15% NaOH, and 1.5 mL of H_2O, followed by filtration. The mixture was washed with 5% $NaHCO_3$ and brine, dried over $MgSO_4$ and filtrated. Then the solvent was evaporated under under reduced pressure to afford the deuterated benzyl-α,α-D_2 alcohol, which was used directly without purification to the next step.

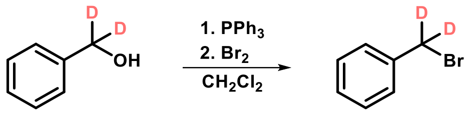

To a solution of triphenylphosphine (14.0 mmol) in dry CH_2Cl_2 (10 mL) under argon atmosphere at 0 °C was added dropwise Br_2 (14.0 mmol). The PPh_3Br_2 complex precipitated immediately. After 1 h of stirring at room temperature, the reaction mixture was cooled at 0 °C, and a solution of the corresponding benzyl-α,α-D_2 alcohol (14.0 mmol) in dry CH_2Cl_2 (5.0 mL) was added dropwise. Triphenylphosphane oxide was precipitated by the addition of pentane and removed by filtration. The filtrate was concentrated, and the crude product was purified by column chromatography using 15-30% AcOEt/hexanes solvent system to give the pure benzyl-α,α-D_2 bromide as a colourless oil (1.21 g, 50%).

^{1}H NMR (500 MHz, $CDCl_3$) δ 7.43-7.39 (m, 2H), 7.38-7.28 (m, 3H).

^{13}C NMR (126 MHz, $CDCl_3$) δ 137.7, 129.0, 128.8, 128.4, 33.1 (p, *J* = 23.3 Hz, -CD_2).

The spectroscopic data are in agreement with those reported.[11]

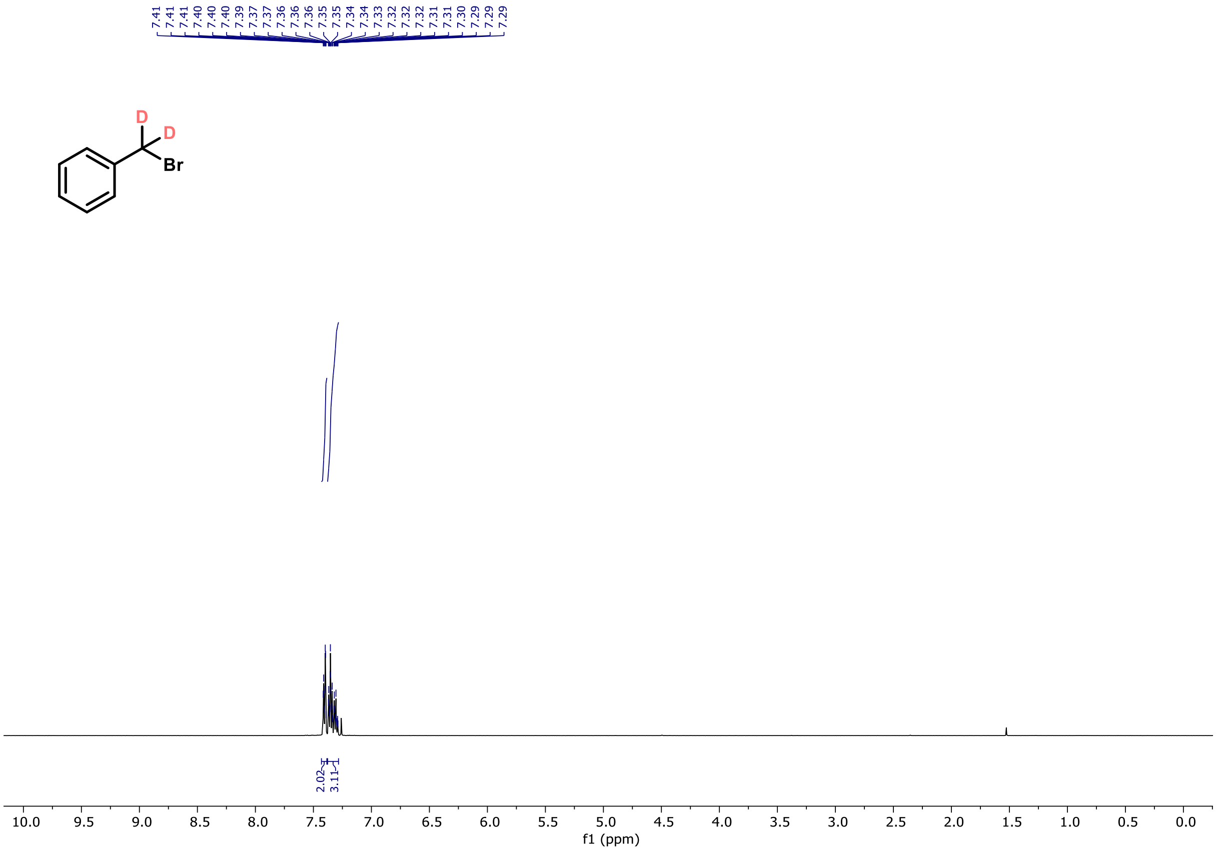

Figure 7. ^{1}H NMR spectrum of benzyl-α,α-D_2 bromide, $CDCl_3$, 500 MHz.

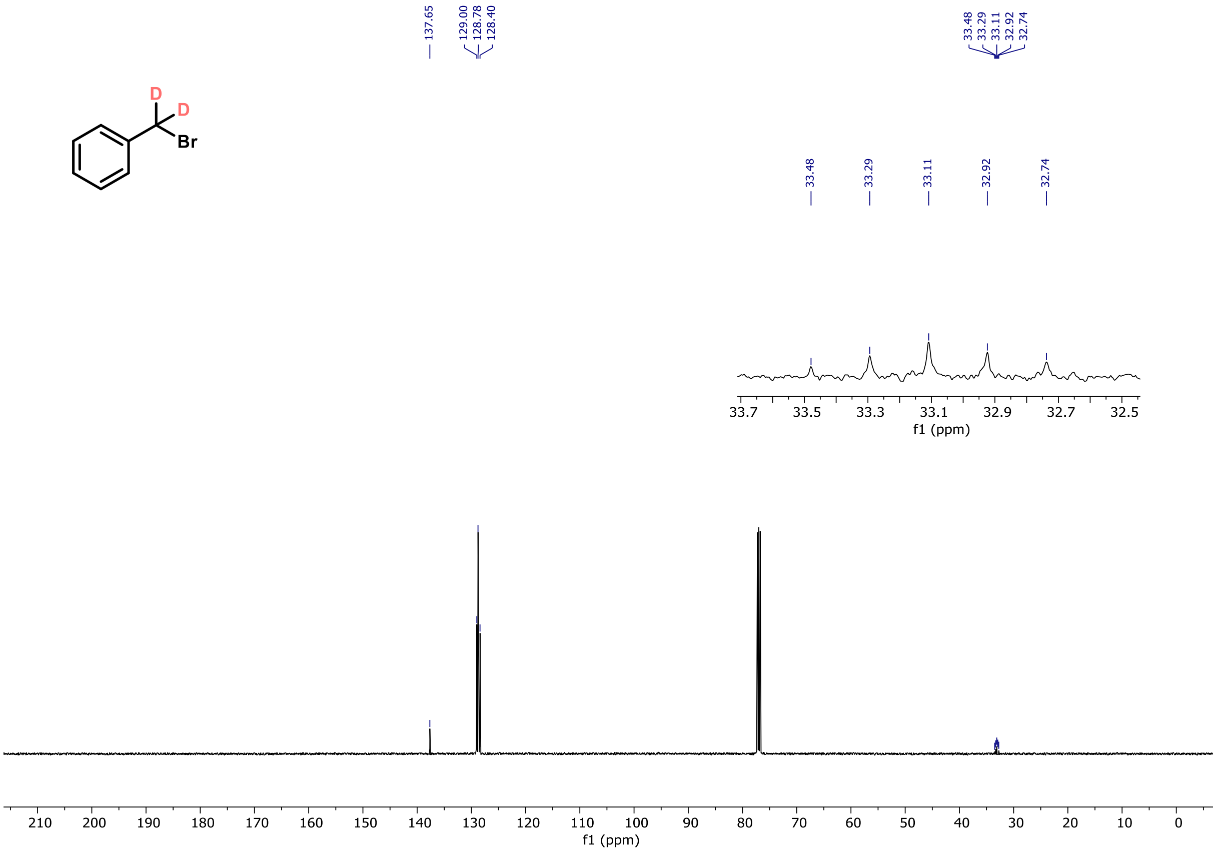

Figure 8. ^{13}C NMR spectrum of benzyl-*α*,*α*-D_2 bromide, $CDCl_3$, 126 MHz.

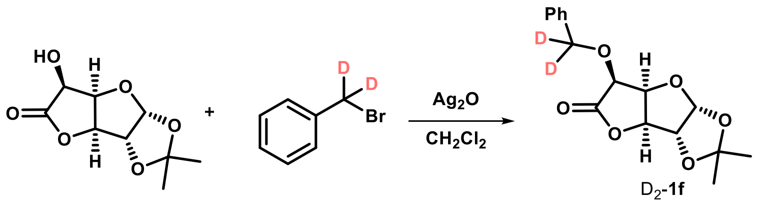

D_2-**1f**

To a solution of acetonide[5] (530.5 mg, 2.5 mmol) and benzyl-*α*,*α*-D_2 bromide (541.6 mg, 3.13 mmol) in dry dichloromethane (4.5 mL) was added silver oxide(I) (347.6 g, 1.5 mmol). After stirring for 10 h in the dark, under argon atmosphere, the reaction mixture was filtered through a pad of Celite and washed with CH_2Cl_2 (30 mL). The filtrate was concentrated in vacuo and the resulting residue was purified by column chromatography using 15-50% MTBE/hexanes solvent system to give the compound D_2-**1f** (692.6 mg, 90%) as a yellowish oil.

$[\alpha]^{25}_{D}$: +41.4 (c = 1.0, $CHCl_3$)

^{1}H NMR (600 MHz, $CDCl_3$) δ 7.45-7.40 (m, 2H), 7.39-7.30 (m, 3H), 6.03 (d, *J* = 3.7 Hz, 1H), 4.84 (dd, *J* = 4.3, 2.9 Hz, 1H), 4.77 (d, *J* = 3.7 Hz), 4.70 (d, *J* = 2.9 Hz, 1H), 4.24 (d, *J* = 4.3 Hz, 1H), 1.50 (s, 3H), 1.33 (s, 3H).

^{13}C NMR (150 MHz, $CDCl_3$) δ 171.9, 136.0, 128.6, 128.4 (2C), 113.1, 106.9, 82.4, 81.6, 77.4, 74.3, 71.8 (p, *J* = 22.0 Hz, -CD_2), 26.8, 26.4.

IR (film) $\tilde{\nu}$: 2989, 2939, 1801, 1449, 1376, 1231, 1163, 1144, 1109, 1035, 947 cm^{-1}.

HRMS (ESI-TOF) m/z calcd for $C_{16}H_{16}D_2O_6Na$ $[M+Na]^+$ 331.1127; found 331.1130.

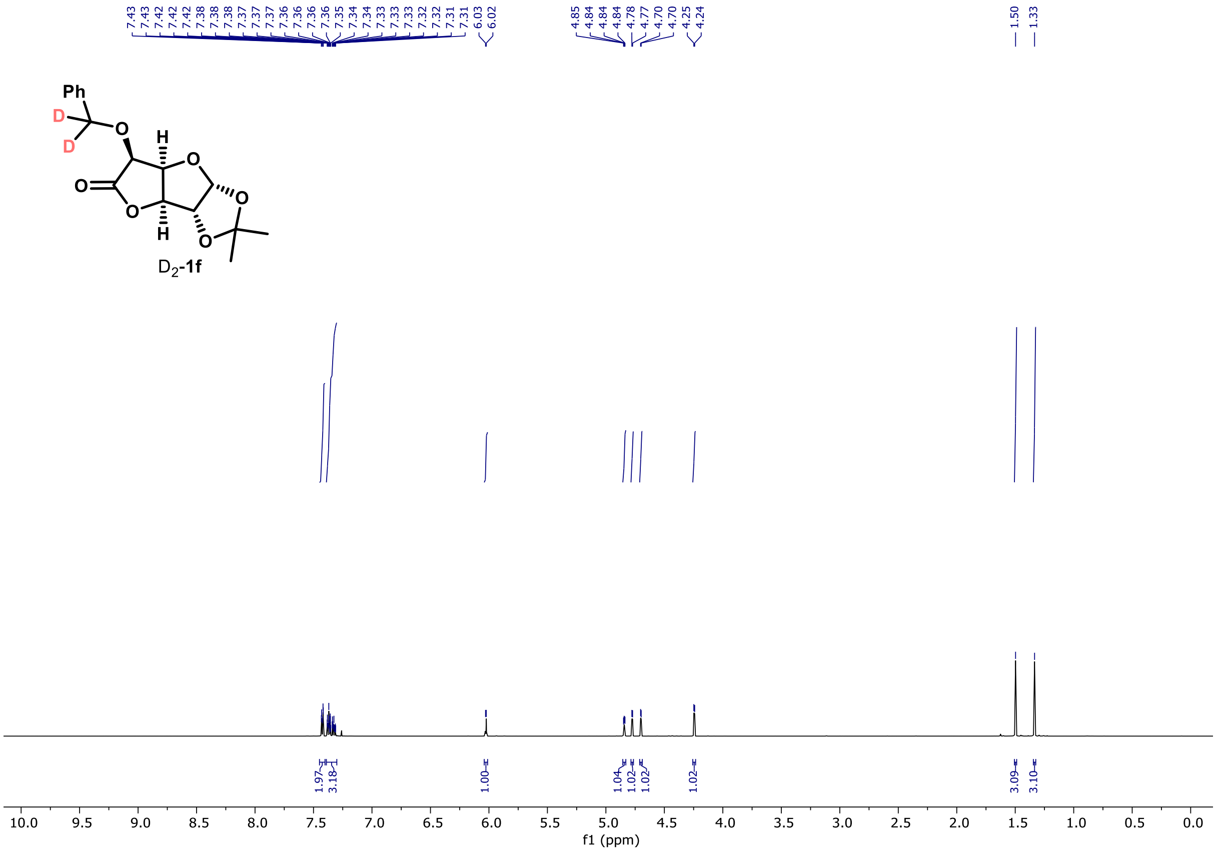

Figure 9. ^{1}H NMR spectrum of D_2-**1f**, $CDCl_3$, 600 MHz.

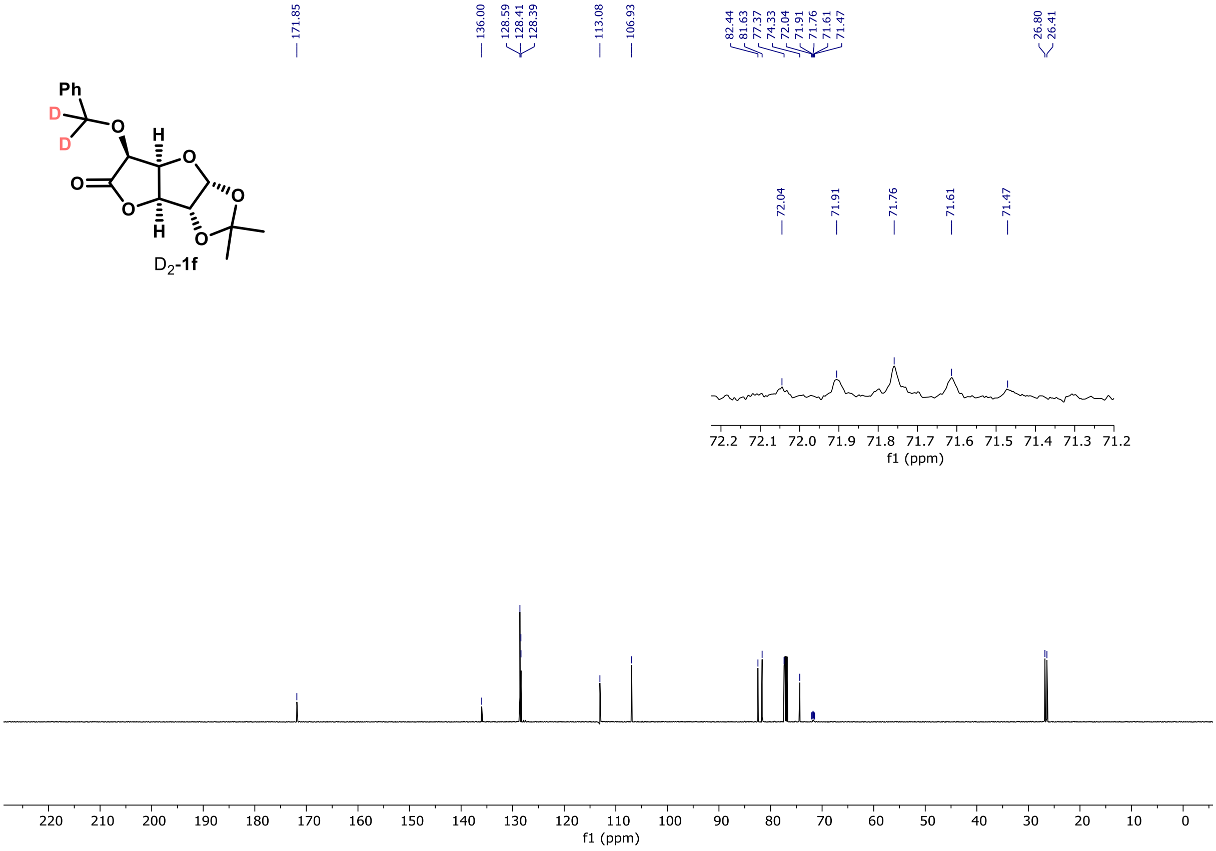

Figure 10. ^{13}C NMR spectrum of D_2-**1f**, $CDCl_3$, 150 MHz.

Photochemical dealkyloxylation of D_2-1f

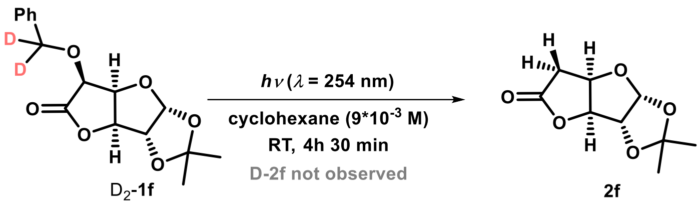

Compound D_2-**1f** was irradiated under standard conditions, and ^1H NMR spectrum showed that deuterium atom was not incorporated into the terminal product **2f**. This result indicated that the *alpha*-hydrogen atom of 2-deoxylactone product **2f** was not originated from the hydrogen of benzyl group in the substrate **1f**.

5.3.3 Reaction was performed with D_2O

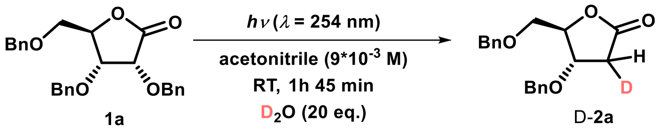

Compounds **1a** was irradiated under standard conditions (cyclohexane was replaced with dry acetonitrile) with 20 equivalents of D_2O and D-**2a** compound was observed (Figures 6 and 7).

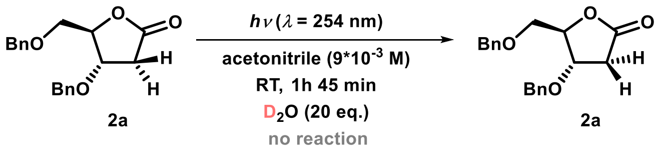

While treating **2a** with 20 equivalents of D_2O under standard conditions (cyclohexane was replaced with dry acetonitrile), no reaction took place and D-**2a** compound was not observed.

This suggests that hydrogen-deuterium exchange occurs, leading to the formation of –OD enol **8**, which subsequently undergoes tautomerisation to produce compound D-**2a**.

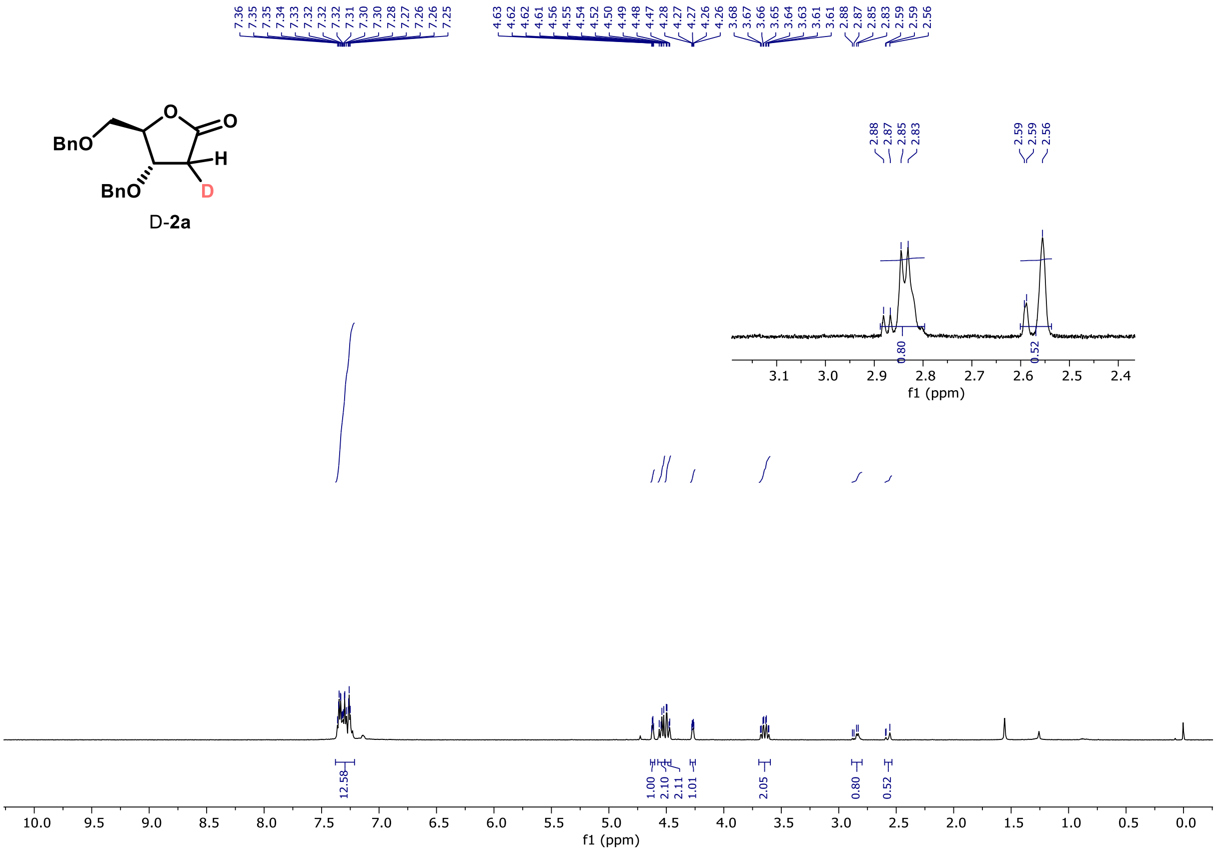

Figure 11. ^{1}H NMR spectrum of the D-**2a**, $CDCl_3$, 500 MHz.

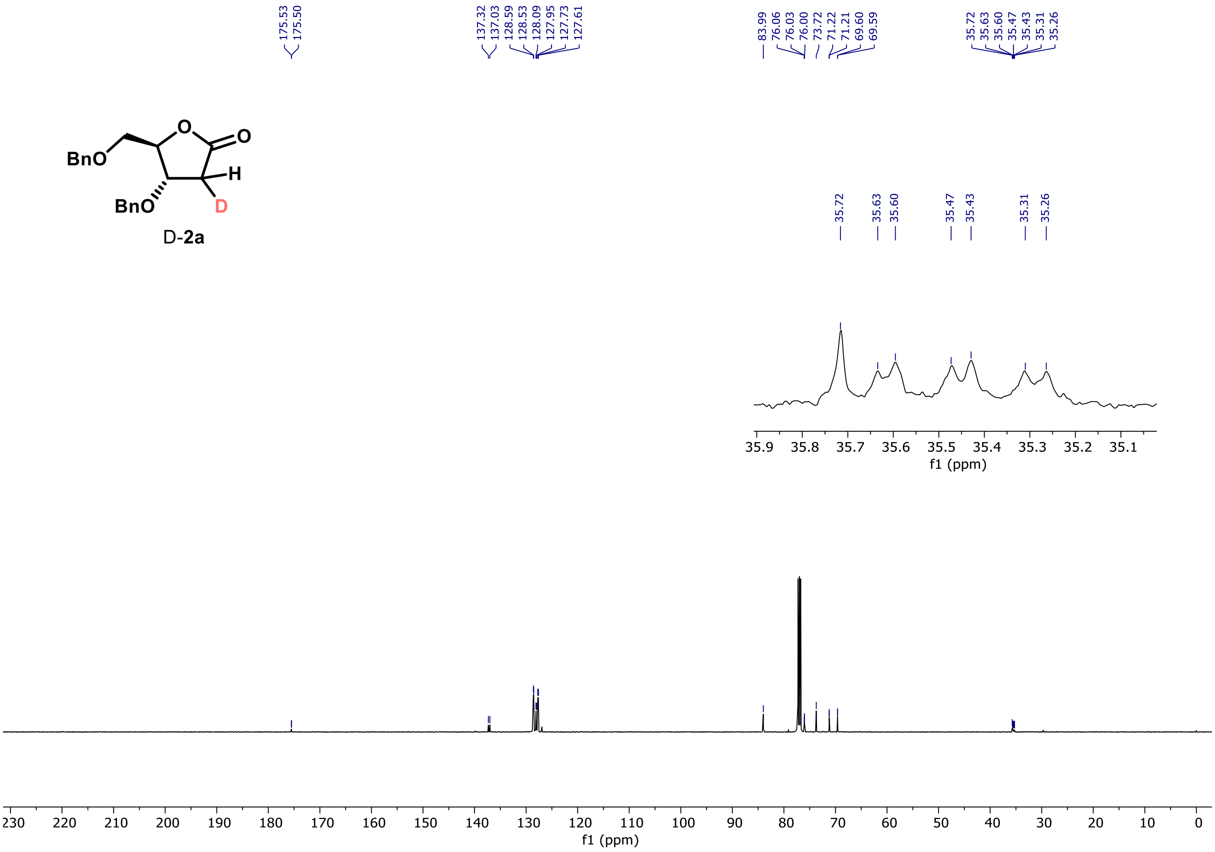

Figure 12. ^{13}C NMR spectrum of the D-**2a**, $CDCl_3$, 126 MHz.

Single Mass Analysis

Tolerance = 10.0 mDa / DBE: min = -1.5, max = 300.0

Element prediction: Off

Number of isotope peaks used for i-FIT = 3

Monoisotopic Mass, Odd and Even Electron Ions

101 formula(e) evaluated with 1 results within limits (up to 50 closest results for each mass)

Elements Used:

C: 0-120 2H: 1-1 O: 2-4 Na: 1-1 1H: 0-200

Mass	Calc. Mass	mDa	PPM	DBE	Formula	i-FIT	i-FIT Norm	Fit Conf %	C	2H	O	Na	1H
336.1320	336.1322	-0.2	-0.6	9.5	C19 2H O4 Na 1H19	1234.9	n/a	n/a	19	1	4	1	19

Figure 13. Single mass analysis (HRMS, ESI-TOF) of the D-**2a.**

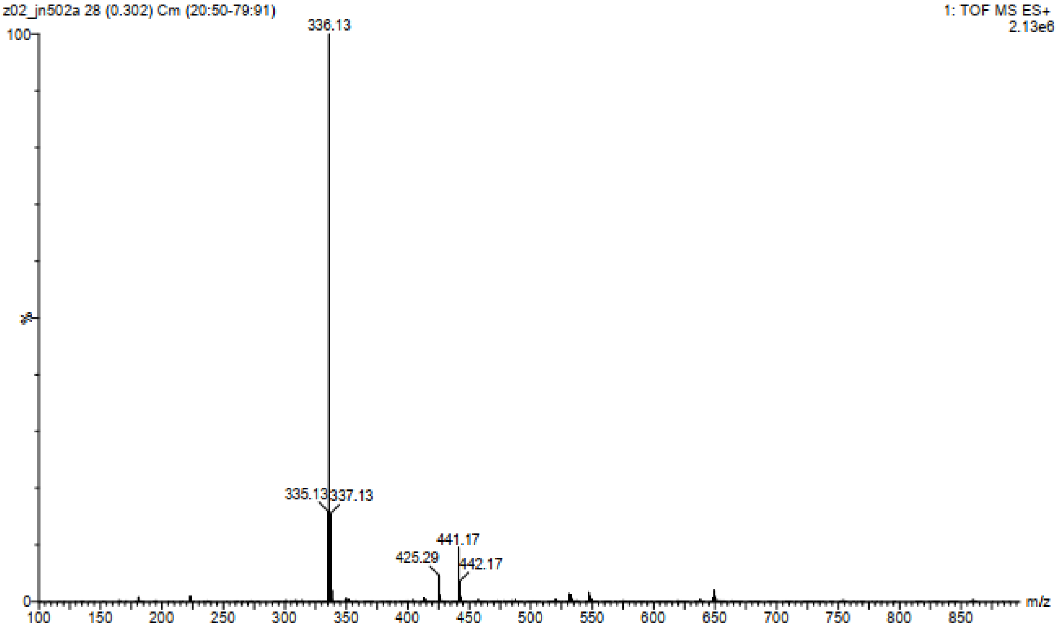

Figure 14. High-resolution mass spectra (HRMS, ESI-TOF) of the D-**2a**.

6. DFT

6.1 General

All the calculations were performed with Gaussian 16 package.[12] DFT calculations were used for calculations involving molecules in S0 and T1 states, and TD DFT formalism for S1 state. Structures of minima and transition states were optimized employing ωB97XD functional[13] and 6-31g(d) basis set. Frequency analysis was performed at the same level to provide correction to thermodynamic functions and confirm the nature of optimized structures (minima and transition states featured zero or one imaginary frequency, respectively). Single point energies were calculated employing M06 functional[14] and 6-311+g(d,p) basis set, and solvation (toluene or water) with the SMD model.[15] In all cases, wavefunctions were tested for stability; if necessary, the unrestricted wavefunctions were obtained using “stable = opt” keyword. Molecular structures were visualized in CYLview.[16]

6.2 Optimized geometries, energies and corrections to thermodynamic functions

I [S0]

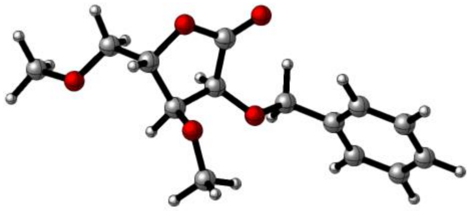

E (ωB97XD/6-31g(d)) = -920.120129622

E (M06/6-311+g(d,p)/SMD(cyclohexane)//ωB97XD/6-31g(d)) = -920.078547919

E (TD-M06/6-311+g(d,p)/SMD(cyclohexane)//ωB97XD/6-31g(d)) = -919.888684628

Zero-point correction=	0.311116 (Hartree/Particle)
Thermal correction to Energy=	0.329485
Thermal correction to Enthalpy=	0.330430
Thermal correction to Gibbs Free Energy=	0.261492

Charge = 0 Multiplicity = 1

C	-0.84315600	1.56899800	0.24825000
O	-2.09369100	1.68128200	0.74570400
C	-2.78633700	0.42124300	0.67653400
C	-1.70682600	-0.64174000	0.43343800
C	-0.67894400	0.17154700	-0.36327200
H	-3.25899500	0.24941700	1.64478200

H	-2.10691500	-1.49631700	-0.12664000
H	-1.01486100	0.23333200	-1.41342300
O	-0.03880000	2.45807100	0.29413300
O	0.61412600	-0.32588900	-0.27220100
O	-1.22220100	-1.03341200	1.68743800
C	-3.84274300	0.51020300	-0.41635600
H	-3.36904600	0.70576500	-1.39470600
H	-4.50964000	1.35970500	-0.20203300
O	-4.54310400	-0.70295000	-0.42941600
C	-5.57122100	-0.72498600	-1.38757900
H	-5.17587700	-0.58846200	-2.40591500
H	-6.05406600	-1.70156700	-1.31964400
H	-6.31762000	0.06020700	-1.19525500
C	-0.52000200	-2.26144200	1.69274400
H	0.40550000	-2.20035100	1.11429800
H	-0.28685300	-2.47525800	2.73755000
H	-1.14967900	-3.07157900	1.29489100
C	1.50985800	0.18378800	-1.25743400
H	1.37108900	-0.36756700	-2.19980000
H	1.29368500	1.24250500	-1.43801600
C	2.91480300	0.03086900	-0.74352200
C	3.29164600	0.71918000	0.41267500
C	3.84376600	-0.77918800	-1.39094300
C	4.58280900	0.59675700	0.90974600
H	2.56042400	1.34850700	0.91234500
C	5.14149600	-0.89849700	-0.89682000
H	3.55290400	-1.32263500	-2.28689600
C	5.51132900	-0.21194400	0.25447400
H	4.86923700	1.13529800	1.80829300
H	5.85985400	-1.53143700	-1.40959400
H	6.52136600	-0.30509900	0.64288200

II [S1]

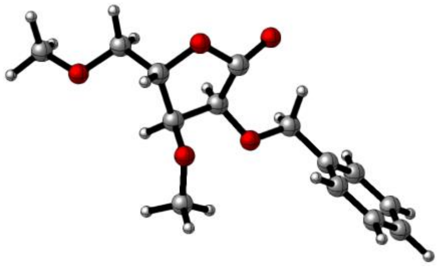

E (TD-ωB97XD/6-31g(d)) = -919.948534028

E (TD-M06/6-311+g(d,p)/SMD(cyclohexane)//TD-ωB97XD/6-31g(d)) = -919.915228513

E (T1) (M06/6-311+g(d,p)/SMD(cyclohexane)// TD-ωB97XD/6-31g(d)) = -919.925511182

Zero-point correction=	0.307053 (Hartree/Particle)
Thermal correction to Energy=	0.325652
Thermal correction to Enthalpy=	0.326596
Thermal correction to Gibbs Free Energy=	0.257111

Charge = 0 Multiplicity = 1

C	-0.81661700	-1.29521700	-0.97476900
O	-2.13431000	-1.20499800	-1.39975300
C	-2.75632100	-0.06089500	-0.80924800
C	-1.68619400	0.70369500	-0.02189400
C	-0.66267500	-0.41651000	0.27207500
H	-3.14741400	0.58037900	-1.60283500
H	-2.10269600	1.11805700	0.90630500
H	-0.93294200	-0.98546100	1.18300000
O	-0.26914300	-2.47088600	-0.92630100
O	0.63311000	0.06814000	0.37395800
O	-1.15909100	1.71568800	-0.82531900
C	-3.89359100	-0.56808300	0.06835000
H	-3.49003000	-1.20890900	0.87184400
H	-4.56776500	-1.19014500	-0.53988100

O	-4.56193300	0.54580100	0.59785600
C	-5.64553500	0.18699100	1.41656000
H	-5.31833400	-0.41516800	2.27835000
H	-6.09780300	1.11239000	1.77863700
H	-6.39997200	-0.38744600	0.85806400
C	-0.53631400	2.76559300	-0.11490400
H	0.35873300	2.42186200	0.41439800
H	-0.25313100	3.51439000	-0.85711600
H	-1.23313200	3.22127800	0.60419700
C	1.58020300	-0.93445300	0.64102900
H	1.44455600	-1.33902100	1.65573700
H	1.35752600	-1.78156600	-0.05805600
C	2.96369600	-0.40100100	0.41941300
C	3.21790700	0.43456000	-0.67086500
C	4.00957200	-0.75975500	1.26819000
C	4.50371400	0.90878500	-0.90121100
H	2.39848500	0.71072100	-1.32744500
C	5.29900500	-0.29141400	1.03187700
H	3.81584800	-1.40640900	2.12065700
C	5.54734600	0.54524000	-0.05199500
H	4.69424200	1.56047500	-1.74866200
H	6.10756600	-0.57500200	1.69890100
H	6.55202900	0.91440000	-0.23526000

TS1 [S1]

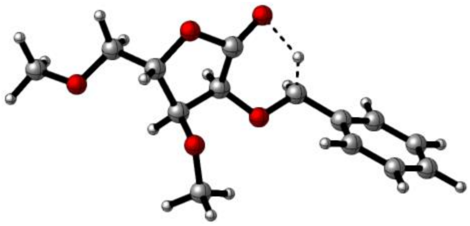

E (TD-ωB97XD/6-31g(d)) = -919.948389469

E (TD-M06/6-311+g(d,p)/SMD(cyclohexane)//TD-ωB97XD/6-31g(d)) = -919.917406064

Zero-point correction=	0.306307 (Hartree/Particle)
Thermal correction to Energy=	0.323942
Thermal correction to Enthalpy=	0.324886
Thermal correction to Gibbs Free Energy=	0.258396

Charge = 0 Multiplicity = 1

C	-0.72593800	-1.25787700	-0.90660500
O	-2.04883700	-1.27208500	-1.32344300
C	-2.71783000	-0.12044800	-0.80113800
C	-1.70853200	0.68159200	0.03725400
C	-0.65655000	-0.39710200	0.35039600
H	-3.06601300	0.50294300	-1.62871100
H	-2.18227400	1.07206900	0.94802900
H	-0.92843000	-0.98358700	1.24988800
O	-0.07085800	-2.38223200	-0.89783500
O	0.63497700	0.11095900	0.48816000
O	-1.19492400	1.72539200	-0.73862600
C	-3.90146900	-0.62473700	0.01264100
H	-3.54002800	-1.23438600	0.85935000
H	-4.52276900	-1.27695800	-0.61995700
O	-4.62374600	0.48968500	0.46483700
C	-5.74969500	0.13145900	1.22377700
H	-5.46653500	-0.43796400	2.12275400
H	-6.24294200	1.05679100	1.52807100
H	-6.45540000	-0.47572400	0.63676700
C	-0.64263300	2.78768900	0.00849400
H	0.24099800	2.47242800	0.57453600
H	-0.35376100	3.55535000	-0.71214900
H	-1.38538100	3.21062000	0.70137600
C	1.58661100	-0.89067100	0.67108100

H	1.47899400	-1.37605600	1.65361300
H	1.30953700	-1.71374300	-0.07743600
C	2.96291800	-0.37264800	0.41349100
C	3.17648300	0.54617200	-0.61777700
C	4.04594900	-0.83087600	1.16387900
C	4.45976500	1.00767000	-0.88329100
H	2.32841000	0.89239200	-1.20027700
C	5.33131800	-0.37355700	0.89150500
H	3.88299500	-1.54537300	1.96709000
C	5.53930500	0.54806300	-0.13091700
H	4.62045300	1.72395700	-1.68331300
H	6.16918400	-0.73239300	1.48144900
H	6.54187200	0.90752300	-0.34256800

III [T1]

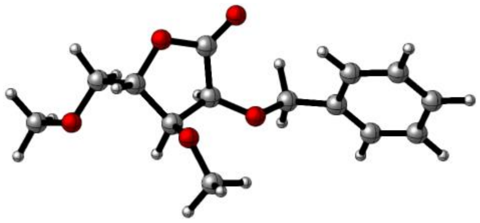

E (ωB97XD/6-31g(d)) = -919.981081839

E (M06/6-311+g(d,p)/SMD(cyclohexane)//ωB97XD/6-31g(d)) = -919.944248126

Zero-point correction=	0.303405 (Hartree/Particle)
Thermal correction to Energy=	0.323035
Thermal correction to Enthalpy=	0.323979
Thermal correction to Gibbs Free Energy=	0.252072

Charge = 0 Multiplicity = 3

C	-0.78163400	1.52802400	0.19799000
O	-2.02282700	1.70065100	0.70090600
C	-2.76397300	0.46705300	0.66774400

C	-1.72814200	-0.64267300	0.44422700
C	-0.67404600	0.11023300	-0.37730200
H	-3.23589600	0.33771800	1.64293000
H	-2.16508500	-1.49377500	-0.09312200
H	-1.01430600	0.16034500	-1.42664400
O	0.05457000	2.38852200	0.21212900
O	0.59922200	-0.43687500	-0.28403000
O	-1.25116300	-1.02374500	1.70450800
C	-3.82393000	0.57007500	-0.42046100
H	-3.34990000	0.72322000	-1.40617400
H	-4.45612700	1.44967200	-0.22308400
O	-4.57056200	-0.61505600	-0.39942300
C	-5.60572700	-0.62032500	-1.35023200
H	-5.21284500	-0.52326600	-2.37401900
H	-6.12508100	-1.57573800	-1.25584200
H	-6.32009600	0.19732500	-1.17202500
C	-0.60203200	-2.28006700	1.73461600
H	0.31642000	-2.27443200	1.14177300
H	-0.36242800	-2.47524000	2.78159100
H	-1.27139200	-3.07295800	1.36800200
C	1.51121700	-0.00352000	-1.30384700
H	1.37315000	-0.62065900	-2.20372000
H	1.28915200	1.04418700	-1.55552700
C	2.88964000	-0.10833500	-0.77433000
C	3.24409900	0.76004700	0.42385600
C	3.87708400	-0.92002500	-1.33477300
C	4.54740700	0.78308700	0.88576200
H	2.45562200	1.35476500	0.87004700
C	5.16375400	-0.91476400	-0.84778400
H	3.62070700	-1.56109000	-2.17599600
C	5.52940900	-0.00555500	0.30495700

H	4.80345700	1.42491300	1.72470100
H	5.92828200	-1.55285200	-1.27664100
H	6.55368300	0.01035100	0.65850300

TS2 [T1]

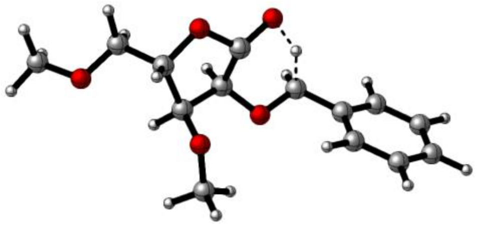

E (ωB97XD/6-31g(d)) = -919.961186269

E (M06/6-311+g(d,p)/SMD(cyclohexane)//ωB97XD/6-31g(d)) = -919.920278315

Zero-point correction=	0.303300 (Hartree/Particle)
Thermal correction to Energy=	0.321121
Thermal correction to Enthalpy=	0.322065
Thermal correction to Gibbs Free Energy=	0.254367

Charge = 0 Multiplicity = 3

C	-0.60826400	-1.12124300	-0.88037500
O	-1.92063900	-1.30493500	-1.26612900
C	-2.66950700	-0.17395600	-0.79554800
C	-1.74388900	0.68210000	0.10073400
C	-0.64794000	-0.33613900	0.42020500
H	-2.98960400	0.43543200	-1.64506100
H	-2.28278000	1.02900000	0.99266600
H	-0.95292700	-0.99238800	1.25895000
O	0.19659600	-2.17701900	-0.90147700
O	0.63074600	0.18480700	0.67269800
O	-1.27392000	1.77052900	-0.64630900
C	-3.88020500	-0.72424600	-0.05788200
H	-3.55010400	-1.30590800	0.82063300

H	-4.42524000	-1.41270700	-0.72190400
O	-4.68352500	0.35932200	0.32777900
C	-5.83573500	-0.04357900	1.02138800
H	-5.58237600	-0.58462100	1.94654300
H	-6.39223500	0.86019000	1.27778700
H	-6.47261400	-0.69430000	0.40293800
C	-0.78118000	2.83405800	0.13884300
H	0.10398000	2.54248700	0.71559900
H	-0.51171900	3.63307400	-0.55502700
H	-1.55359400	3.20732200	0.82827800
C	1.57957300	-0.81742100	0.74361900
H	1.48883400	-1.43169800	1.65241400
H	1.20341400	-1.63321100	-0.12857000
C	2.94291000	-0.34256400	0.43358500
C	3.12924100	0.69389900	-0.48904700
C	4.05589700	-0.96040400	1.01191000
C	4.41287600	1.11414800	-0.81066600
H	2.25886200	1.15578000	-0.94408900
C	5.33923900	-0.54108500	0.68226500
H	3.91399900	-1.76807800	1.72575100
C	5.51956900	0.49848800	-0.22730900
H	4.55322100	1.92040900	-1.52417000
H	6.19914800	-1.02194600	1.13834000
H	6.52217200	0.82719400	-0.48389500

TS3 [T1]

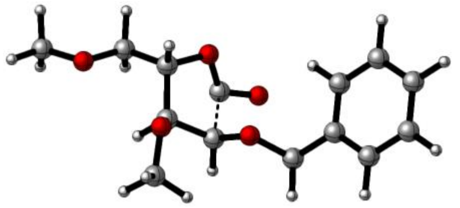

E (ωB97XD/6-31g(d)) = -919.965481393

E (M06/6-311+g(d,p)/SMD(cyclohexane)//ωB97XD/6-31g(d)) = -919.923574312

Zero-point correction=	0.306594 (Hartree/Particle)
Thermal correction to Energy=	0.325028
Thermal correction to Enthalpy=	0.325972
Thermal correction to Gibbs Free Energy=	0.256052

Charge = 0 Multiplicity = 3

C	-0.82452300	-1.31394000	1.23745700
O	-1.10152200	-1.53886200	-0.10170100
C	-2.04054900	-0.57112200	-0.55835000
C	-1.77190200	0.74958700	0.19656700
C	-0.53949600	0.50752600	1.07769900
H	-1.84773500	-0.41682200	-1.62136200
H	-2.61960800	0.98068400	0.86042800
H	-0.56098100	0.86485000	2.11124100
O	0.16856400	-1.80816400	1.79305800
O	0.60932600	0.72939100	0.41772200
O	-1.60106500	1.76830100	-0.74314700
C	-3.45232200	-1.09839000	-0.33928800
H	-3.58822100	-1.32359800	0.73268200
H	-3.58365200	-2.03836100	-0.89783900
O	-4.35467500	-0.11613600	-0.77094700
C	-5.69364900	-0.49696400	-0.58855300
H	-5.91949300	-0.68425100	0.47284500
H	-6.31586300	0.32574600	-0.94663100
H	-5.93658100	-1.40589500	-1.15992800
C	-1.51272400	3.05352000	-0.17899900
H	-0.59774400	3.17381600	0.41776700
H	-1.48649300	3.76069200	-1.00978200
H	-2.38535000	3.27414700	0.45441400

C	1.80507200	0.69970700	1.20745100
H	1.98364400	1.70596900	1.60724100
H	1.65123900	0.00411500	2.03981600
C	2.94202300	0.24007400	0.33918000
C	2.84196100	-0.98749400	-0.32065400
C	4.09587500	1.00609900	0.19632100
C	3.88943600	-1.43769800	-1.11398800
H	1.93709900	-1.57735900	-0.20664700
C	5.14993000	0.55011200	-0.59246500
H	4.17364500	1.96577600	0.70198200
C	5.04643000	-0.67116100	-1.24954300
H	3.80581000	-2.39133000	-1.62660600
H	6.04702200	1.15298400	-0.69775500
H	5.86485800	-1.02653200	-1.86876400

IV [T1]

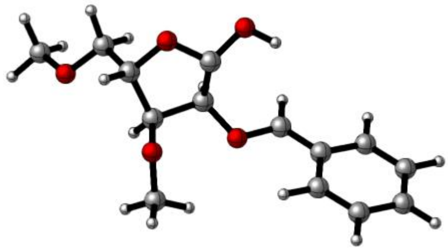

E (ωB97XD/6-31g(d)) = -920.000347248

E (M06/6-311+g(d,p)/SMD(cyclohexane)//ωB97XD/6-31g(d)) = -919.963994272

E (S1) (M06/6-311+g(d,p)/SMD(cyclohexane)//ωB97XD/6-31g(d)) = -919.963856060 (unrestricted)

Zero-point correction=	0.306720 (Hartree/Particle)
Thermal correction to Energy=	0.325842
Thermal correction to Enthalpy=	0.326786
Thermal correction to Gibbs Free Energy=	0.255688

Charge = 0 Multiplicity = 3

C	-0.76542800	-1.15384300	-1.02164400
O	-2.05725800	-1.13186100	-1.45700600
C	-2.76477700	-0.06475400	-0.81384700
C	-1.73466600	0.74819500	-0.01502200
C	-0.64636100	-0.30930900	0.22260600
H	-3.21685700	0.57166600	-1.57757200
H	-2.17333300	1.12243500	0.91874900
H	-0.88365400	-0.90991100	1.12068700
O	-0.24435100	-2.41385300	-1.05951700
O	0.63295400	0.26967100	0.38920400
O	-1.28739500	1.80784700	-0.81633800
C	-3.84660800	-0.69279800	0.05511000
H	-3.38535000	-1.33005000	0.83009300
H	-4.47714500	-1.34298300	-0.57053600
O	-4.59324400	0.34399100	0.63338500
C	-5.63069400	-0.12954500	1.45327700
H	-5.24197000	-0.73231300	2.28876000
H	-6.15018300	0.74305500	1.85416500
H	-6.34540400	-0.74394200	0.88502700
C	-0.75646000	2.89820400	-0.09425600
H	0.14412400	2.62103400	0.46390700
H	-0.50402900	3.66328600	-0.83135800
H	-1.50192200	3.30870600	0.60354500
C	1.60937800	-0.58759100	0.76393800
H	1.31224000	-1.45534600	1.35113400
H	0.71616800	-2.32264500	-1.11611800
C	2.95606700	-0.27003400	0.49646800
C	3.31481800	0.86057800	-0.28104700
C	3.99429200	-1.09573100	0.99822700
C	4.64661200	1.14083200	-0.53383600
H	2.52954300	1.49307700	-0.68135600

C	5.31964800	-0.80161600	0.73338300
H	3.73854100	-1.96534800	1.59893900
C	5.65876400	0.31807900	-0.03220700
H	4.90486400	2.00955600	-1.13286200
H	6.10084600	-1.44591500	1.12650300
H	6.70036700	0.54527800	-0.23642900

TS4 [T1]

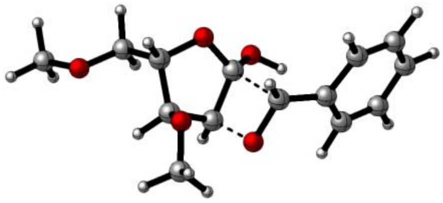

E (ωB97XD/6-31g(d)) = -919.939148351

E (M06/6-311+g(d,p)/SMD(cyclohexane)//ωB97XD/6-31g(d)) = -919.906000043

Zero-point correction=	0.303873 (Hartree/Particle)
Thermal correction to Energy=	0.322294
Thermal correction to Enthalpy=	0.323238
Thermal correction to Gibbs Free Energy=	0.254994

Charge = 0 Multiplicity = 3

C	-0.01081100	-0.63650500	0.04679000
O	0.86123800	-0.93317400	1.04899000
C	2.11989400	-0.28898400	0.81357500
C	1.85463500	0.88252100	-0.13844800
C	0.57852400	0.41260700	-0.89115600
H	2.50065500	0.08036200	1.76738300
H	2.70802900	1.02897800	-0.81390900
H	0.77116300	0.08915300	-1.91958900
O	-0.62326900	-1.76359400	-0.39941400
O	-0.50295800	1.33118300	-0.84292500

O	1.63531400	2.02869700	0.63746500
C	3.06604000	-1.33062200	0.22795100
H	2.66213800	-1.70387200	-0.73026000
H	3.12448000	-2.18916100	0.91407600
O	4.31952400	-0.72896900	0.04819500
C	5.26950700	-1.61127500	-0.49295100
H	4.95926100	-1.98049100	-1.48271600
H	6.20434200	-1.05670900	-0.59539000
H	5.43548100	-2.47792400	0.16462900
C	1.69206100	3.23057300	-0.10179800
H	0.90687900	3.27236000	-0.86385300
H	1.54466700	4.04266500	0.61284300
H	2.67663500	3.34836200	-0.57926600
C	-1.18104700	0.79592500	0.28497000
H	-0.83524300	1.19614600	1.24298100
H	-1.50451300	-1.52936000	-0.73050100
C	-2.53879200	0.50671900	0.14277700
C	-3.11110100	0.17307700	-1.18644100
C	-3.34483300	0.14441300	1.33095300
C	-4.23672100	-0.57287400	-1.27620900
H	-2.59959600	0.54074900	-2.06999300
C	-4.47544400	-0.58306200	1.20141400
H	-2.99373700	0.46503100	2.30818700
C	-4.93906900	-1.00817300	-0.09569800
H	-4.64523200	-0.82467800	-2.25151000
H	-5.05522300	-0.85094300	2.08001900
H	-5.83602600	-1.60986400	-0.18598600

TS5 [T1]

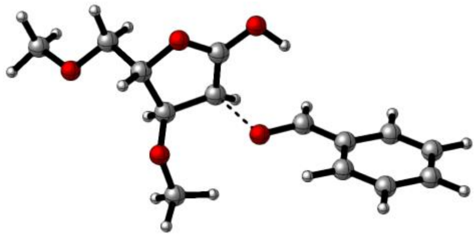

E (ωB97XD/6-31g(d)) = -919.979033885

E (M06/6-311+g(d,p)/SMD(cyclohexane)//ωB97XD/6-31g(d)) = -919.949031824

Zero-point correction=	0.305234 (Hartree/Particle)
Thermal correction to Energy=	0.323817
Thermal correction to Enthalpy=	0.324761
Thermal correction to Gibbs Free Energy=	0.255318

Charge = 0 Multiplicity = 3

C	0.91548300	-1.38215100	0.15177600
O	1.88906300	-1.35931600	1.05179900
C	2.73213900	-0.21904200	0.76126000
C	1.96749700	0.65320400	-0.25410900
C	0.83174100	-0.26568400	-0.67232800
H	2.87175200	0.33051500	1.69229900
H	2.61237400	0.89317300	-1.11377200
H	0.47185800	-0.31972000	-1.69099600
O	0.10537500	-2.41538100	0.23678600
O	-0.87454500	0.44332500	-0.04359500
O	1.58528200	1.83331000	0.39378600
C	4.05149800	-0.76184900	0.23609300
H	3.86624800	-1.33569000	-0.68966700
H	4.48656400	-1.45295200	0.97453700
O	4.88954600	0.33228500	-0.00351500
C	6.14289100	-0.04664400	-0.51532800
H	6.04241500	-0.57436500	-1.47613000

H	6.71747700	0.86860400	-0.66800500
H	6.68379000	-0.69893800	0.18647500
C	1.01407400	2.79074300	-0.47121900
H	0.06260600	2.43560800	-0.88116100
H	0.83499600	3.68356800	0.13032700
H	1.70532200	3.04331800	-1.29070200
C	-1.83119500	-0.21819500	-0.67986600
H	-1.58927800	-0.72709800	-1.62191400
H	-0.76817400	-2.13714200	-0.10756300
C	-3.20166000	-0.07300600	-0.32261800
C	-3.59353300	0.67866300	0.81148700
C	-4.21515000	-0.70195200	-1.08604500
C	-4.93127300	0.78909900	1.15372400
H	-2.82625900	1.16419500	1.40559200
C	-5.54732600	-0.58712700	-0.72847100
H	-3.93711000	-1.28052000	-1.96485200
C	-5.91957800	0.15933900	0.39322100
H	-5.21233500	1.37191800	2.02694000
H	-6.30804700	-1.07887000	-1.32885600
H	-6.96562300	0.24822600	0.66990800

V [T1]

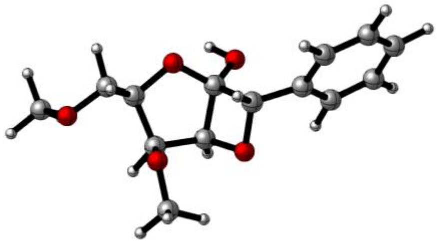

E (ωB97XD/6-31g(d)) = -919.945348772

E (M06/6-311+g(d,p)/SMD(cyclohexane)//ωB97XD/6-31g(d)) = -919.951192054

Zero-point correction=	0.303681 (Hartree/Particle)
Thermal correction to Energy=	0.322745

Thermal correction to Enthalpy= 0.323689

Thermal correction to Gibbs Free Energy= 0.254109

Charge = 0 Multiplicity = 3

C	-0.85654900	-1.88491800	-0.51077200
O	-1.52424400	-1.11602500	-1.36042200
C	-2.47208900	-0.17940600	-0.81577200
C	-1.79618300	1.11428700	-0.33849900
C	-0.78364200	0.90713400	0.73982400
H	-3.10392000	0.07489700	-1.66879100
H	-2.60553300	1.72582700	0.09321000
H	-0.82131400	1.44747900	1.68496600
O	-0.02071600	-2.68579100	-0.78632300
O	0.42011800	0.44809300	0.33469700
O	-1.28868700	1.72470600	-1.50815400
C	-3.32805300	-0.83146900	0.26375300
H	-2.72298600	-1.01599500	1.16671500
H	-3.69722000	-1.80284900	-0.10281300
O	-4.38913200	0.04463500	0.53810200
C	-5.23353500	-0.43447100	1.55407400
H	-4.68879900	-0.56802200	2.50112200
H	-6.02109000	0.30816000	1.69539300
H	-5.69130800	-1.39605500	1.27699700
C	-0.68471900	2.97445300	-1.28230900
H	0.23840600	2.88071900	-0.69595700
H	-0.44088800	3.38460900	-2.26434700
H	-1.36841400	3.66669300	-0.76547500
C	1.32941100	0.09636500	1.37734500
H	1.30675000	0.87027800	2.15723400
H	0.99142300	-0.84886000	1.82033600
C	2.71135300	-0.03341100	0.79933700

C	2.98564900	-1.03531600	-0.13491300
C	3.72631700	0.84208100	1.18083700
C	4.25954400	-1.15301200	-0.67799100
H	2.19588100	-1.71646500	-0.43876800
C	5.00534700	0.71927100	0.64344500
H	3.51747800	1.62712800	1.90399900
C	5.27238200	-0.27825200	-0.28829400
H	4.46389300	-1.93232200	-1.40604400
H	5.78960800	1.40503100	0.95014100
H	6.26792500	-0.37521200	-0.71152000

PhCHO [S0]

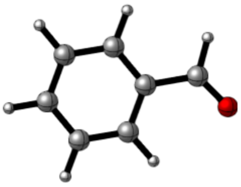

E (ωB97XD/6-31g(d)) = -345.452706265

E (M06/6-311+g(d,p)/SMD(cyclohexane)//ωB97XD/6-31g(d)) = -345.421099585

Zero-point correction=	0.111434 (Hartree/Particle)
Thermal correction to Energy=	0.117683
Thermal correction to Enthalpy=	0.118627
Thermal correction to Gibbs Free Energy=	0.080924

Charge = 0 Multiplicity = 1

O	2.83738500	-0.39597600	-0.00006000
C	1.99006700	0.46827500	0.00012300
H	2.27542300	1.54277400	-0.00049400
C	0.53031000	0.21387900	0.00004700
C	0.04592000	-1.09741600	0.00002900
C	-0.35985200	1.28852400	0.00001700
C	-1.32261600	-1.32738600	-0.00001900

H	0.75849600	-1.91640500	0.00006100
C	-1.73154800	1.05762200	-0.00000200
H	0.02446500	2.30630900	0.00002300
C	-2.21024900	-0.25019200	-0.00002900
H	-1.70340200	-2.34411700	-0.00004400
H	-2.42540900	1.89248200	-0.00000700
H	-3.28084800	-0.43307600	-0.00006100

VI [S0]

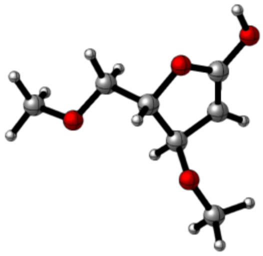

E (ωB97XD/6-31g(d)) = -574.622231136

E (M06/6-311+g(d,p)/SMD(cyclohexane)//ωB97XD/6-31g(d)) = -574.628521789

Zero-point correction=	0.194555 (Hartree/Particle)
Thermal correction to Energy=	0.206214
Thermal correction to Enthalpy=	0.207159
Thermal correction to Gibbs Free Energy=	0.156346

Charge = 0 Multiplicity = 1

C	1.52625100	-1.21871000	0.18389900
O	0.60896500	-1.37394600	-0.80284800
C	-0.28735800	-0.24671400	-0.69575800
C	0.41233800	0.77901800	0.21325900
C	1.46317100	-0.07263500	0.86838000
H	-0.43418600	0.16771800	-1.69414700
H	-0.30415700	1.22130000	0.92192600
H	2.10098800	0.20043400	1.69449900
O	2.35767100	-2.25285500	0.32859600

O	0.91359100	1.81271400	-0.62166900
C	-1.59686200	-0.76894000	-0.12518600
H	-1.41654100	-1.17129900	0.88652400
H	-1.96908900	-1.59320200	-0.75427000
O	-2.50818300	0.29543800	-0.09202300
C	-3.74257100	-0.06903900	0.46769800
H	-3.62911100	-0.41170700	1.50800800
H	-4.37954400	0.81760700	0.45152900
H	-4.22826700	-0.87046400	-0.11044200
C	1.55973300	2.82973700	0.09535400
H	2.47009700	2.46750800	0.59535400
H	1.83804800	3.60365300	-0.62357400
H	0.89694700	3.27309600	0.85652000
H	2.07024700	-2.95174900	-0.27425200

7. Copies of ^{1}H and ^{13}C NMR spectra of substrates and products

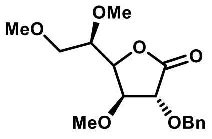

1e

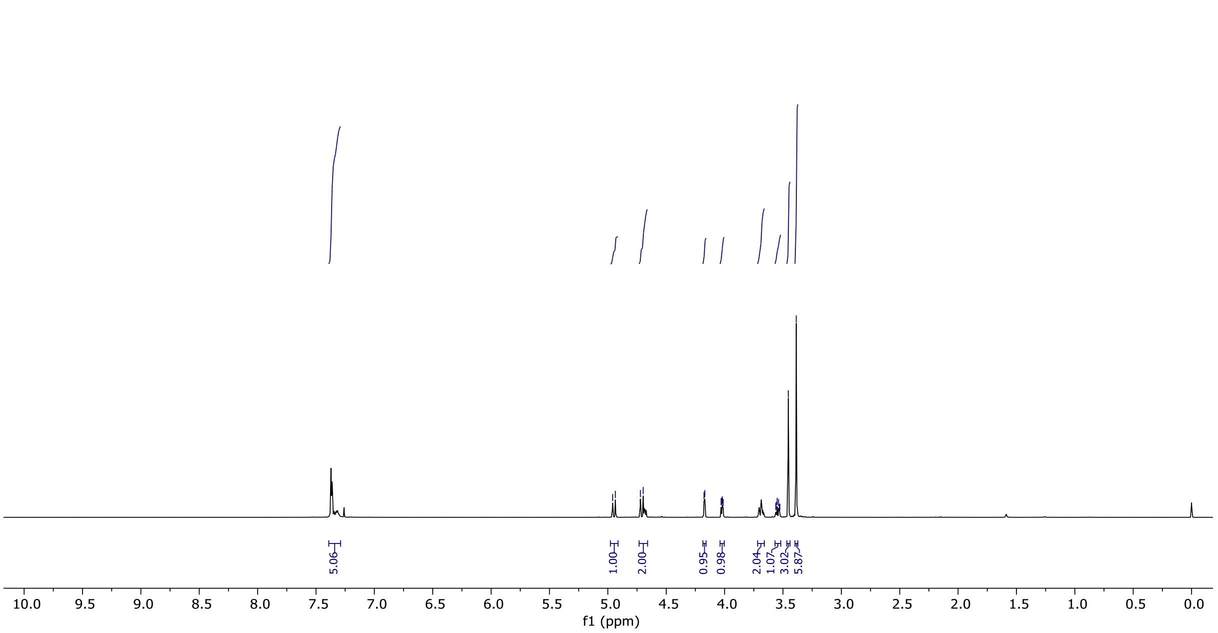

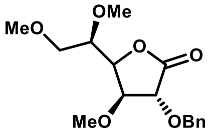

1e

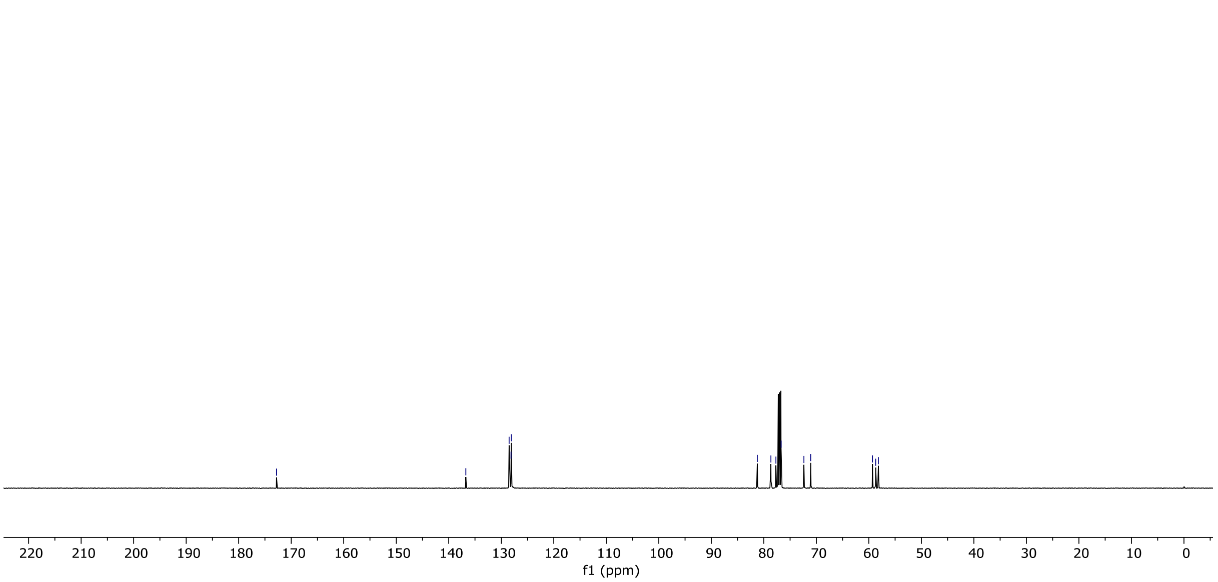

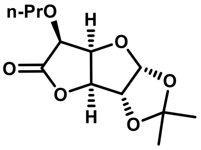

n-PrO
H
O
O
O
O
H
O

1h

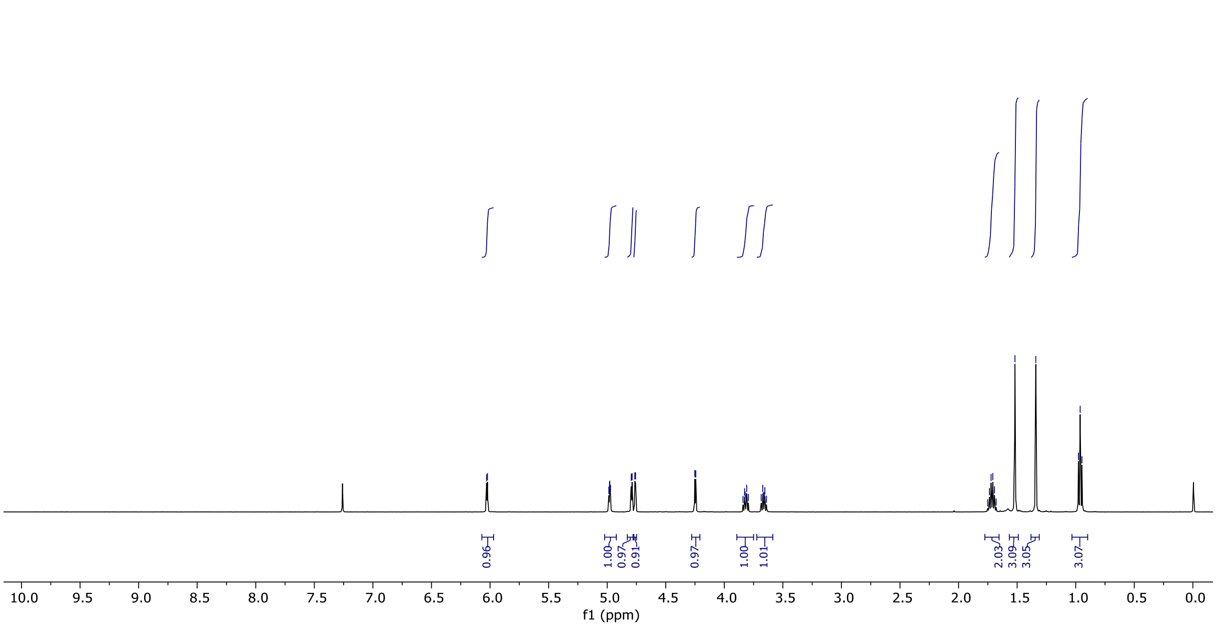

6.03
6.02
4.99
4.98
4.98
4.97
4.79
4.79
4.76
4.76
4.25
4.24
3.84
3.83
3.82
3.81
3.81
3.80
3.69
3.67
3.67
3.66
3.65
3.64
1.75
1.74
1.72
1.71
1.69
1.68
1.52
1.34
0.98
0.96
0.95
0.96
1.00
0.97
0.91
0.97
1.00
1.01
2.03
3.09
3.05
3.07
10.0 9.5 9.0 8.5 8.0 7.5 7.0 6.5 6.0 5.5 5.0 4.5 4.0 3.5 3.0 2.5 2.0 1.5 1.0 0.5 0.0
f1 (ppm)

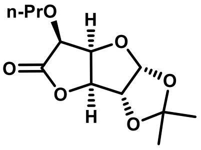

n-PrO
H
O
O
O
O
H
O

1h

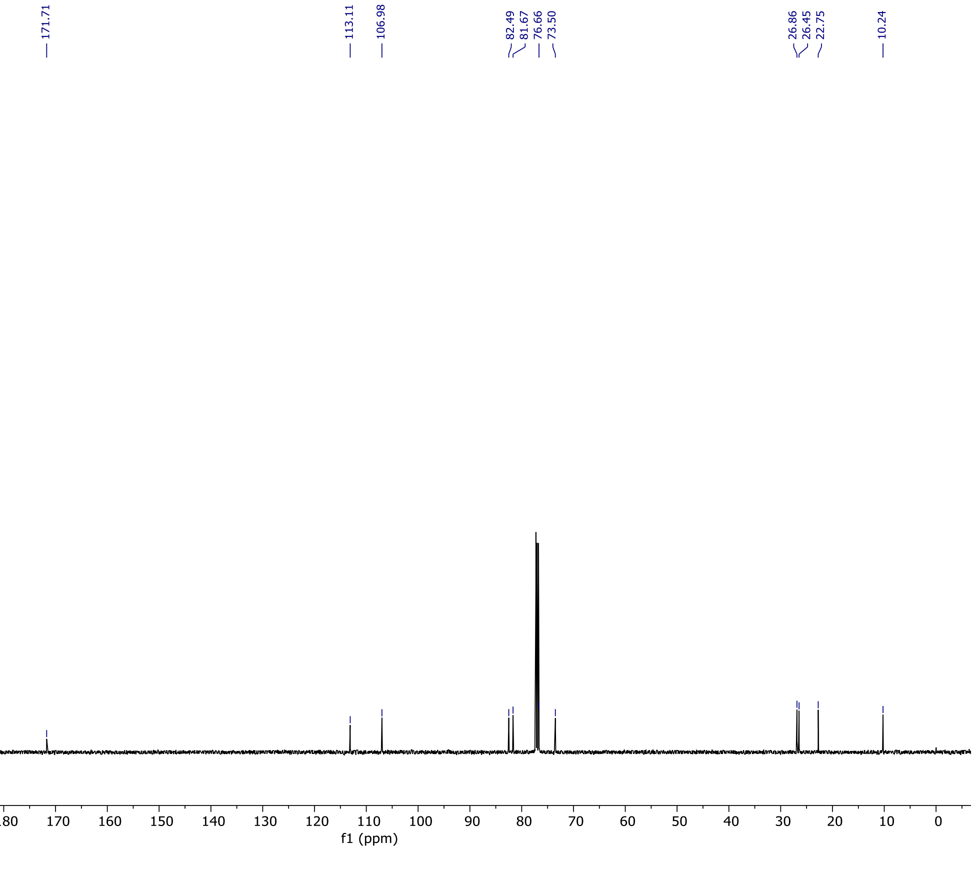

171.71
113.11
106.98
82.49
81.67
76.66
73.50
26.86
26.45
22.75
10.24
220 210 200 190 180 170 160 150 140 130 120 110 100 90 80 70 60 50 40 30 20 10 0
f1 (ppm)

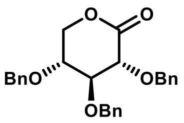
O
O
BnO
OBn
OBn

1i

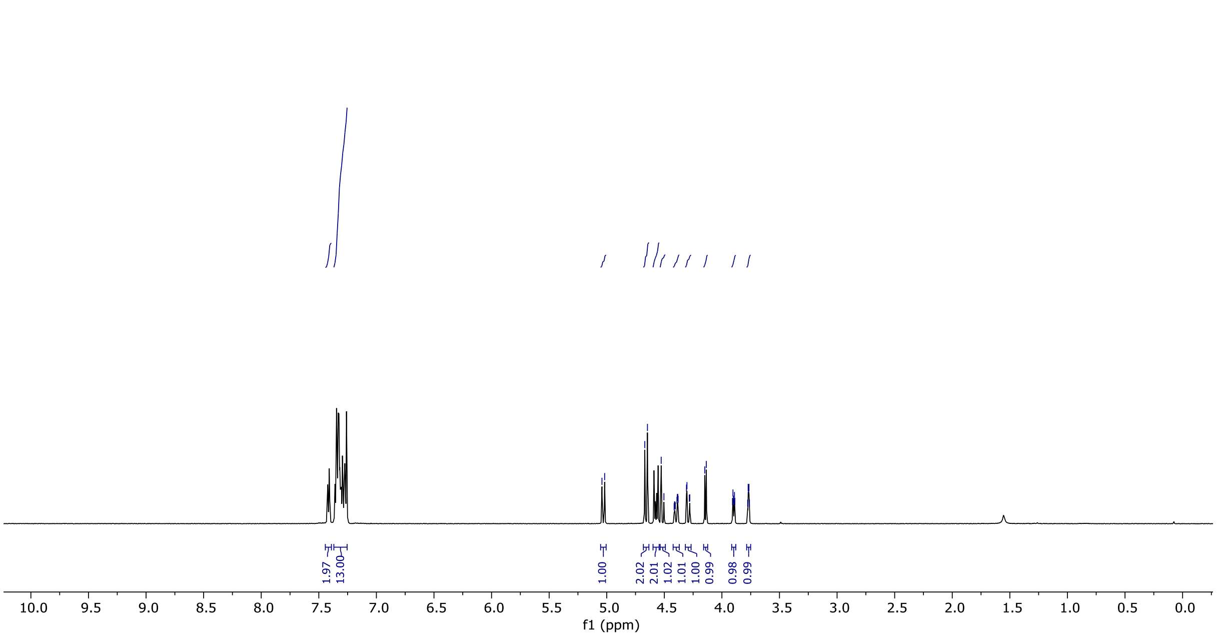
5.04
5.02
4.67
4.65
4.53
4.50
4.41
4.41
4.41
4.40
4.39
4.39
4.38
4.38
4.31
4.30
4.28
4.28
4.15
4.14
3.91
3.90
3.90
3.89
3.89
3.89
3.78
3.77
3.77
3.77
3.76
3.76
1.97
13.00
1.00
2.02
2.01
1.02
1.01
1.00
0.99
0.98
0.99
f1 (ppm)

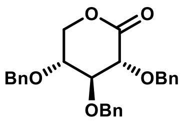
O
O
BnO
OBn
OBn

1i

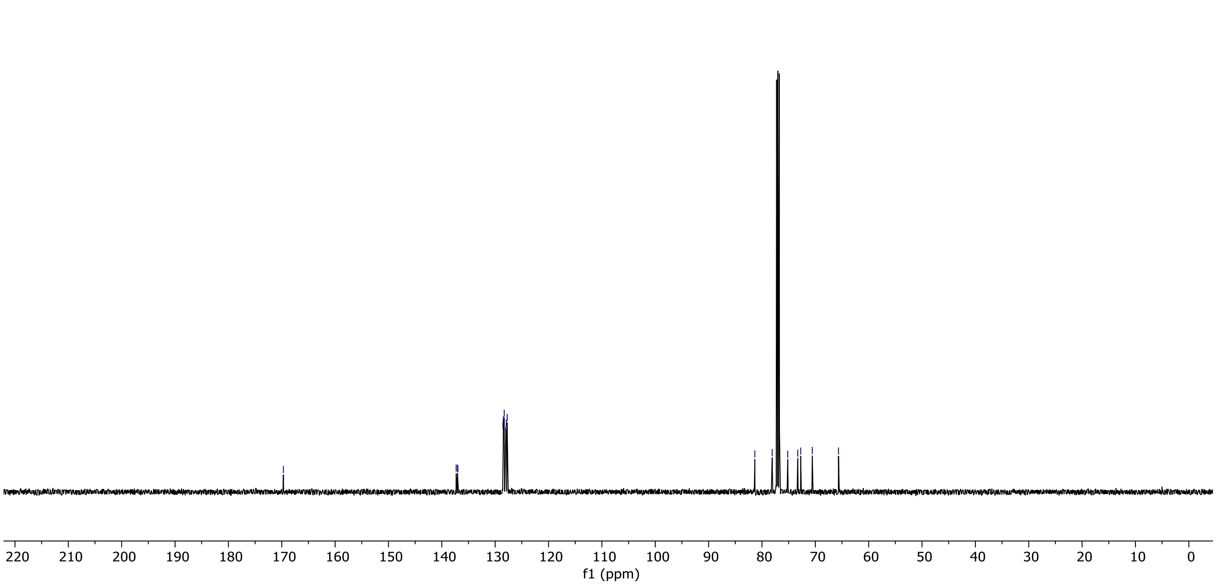
169.69
137.32
137.06
136.99
128.54
128.48
128.45
128.31
128.06
128.03
127.92
127.75
81.33
78.09
75.17
73.29
72.74
70.57
65.65
f1 (ppm)

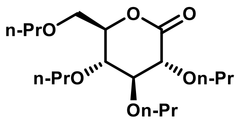
n-PrO
O
O
n-PrO
On-Pr
On-Pr

1m

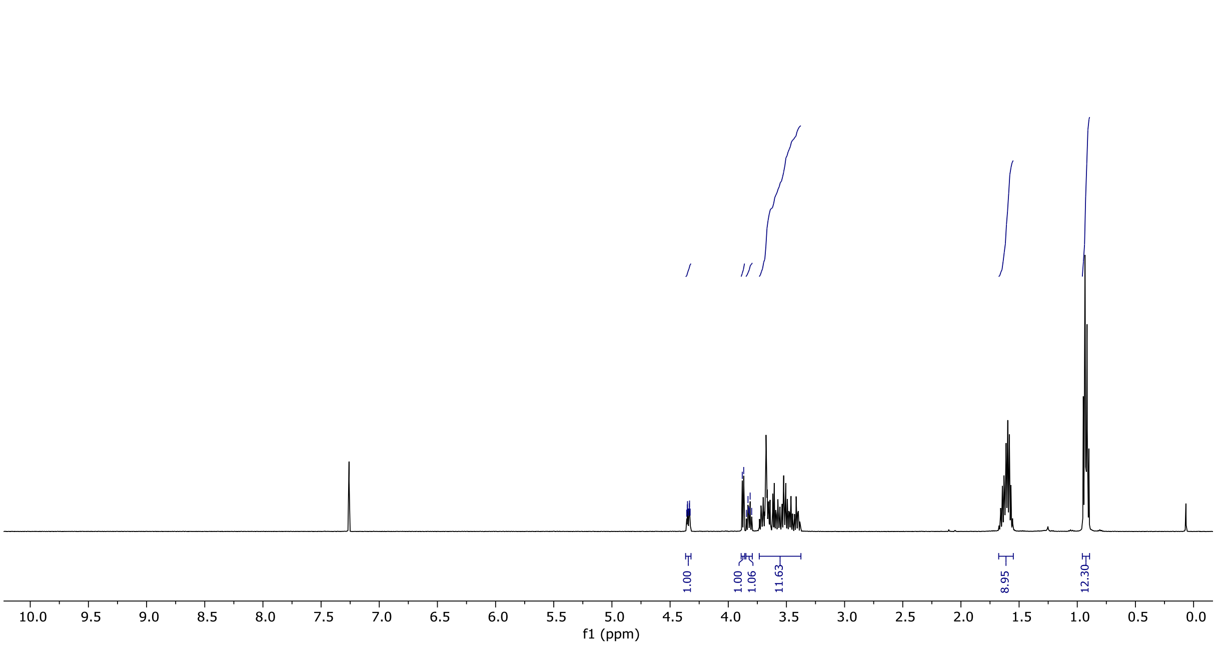
4.36
4.35
4.35
4.35
4.34
4.33
4.33
4.33
3.88
3.87
3.84
3.83
3.83
3.82
3.81
3.80
1.00
1.00
1.06
11.63
8.95
12.30
10.0 9.5 9.0 8.5 8.0 7.5 7.0 6.5 6.0 5.5 5.0 4.5 4.0 3.5 3.0 2.5 2.0 1.5 1.0 0.5 0.0
f1 (ppm)

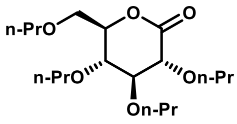
n-PrO
O
O
n-PrO
On-Pr
On-Pr

1m

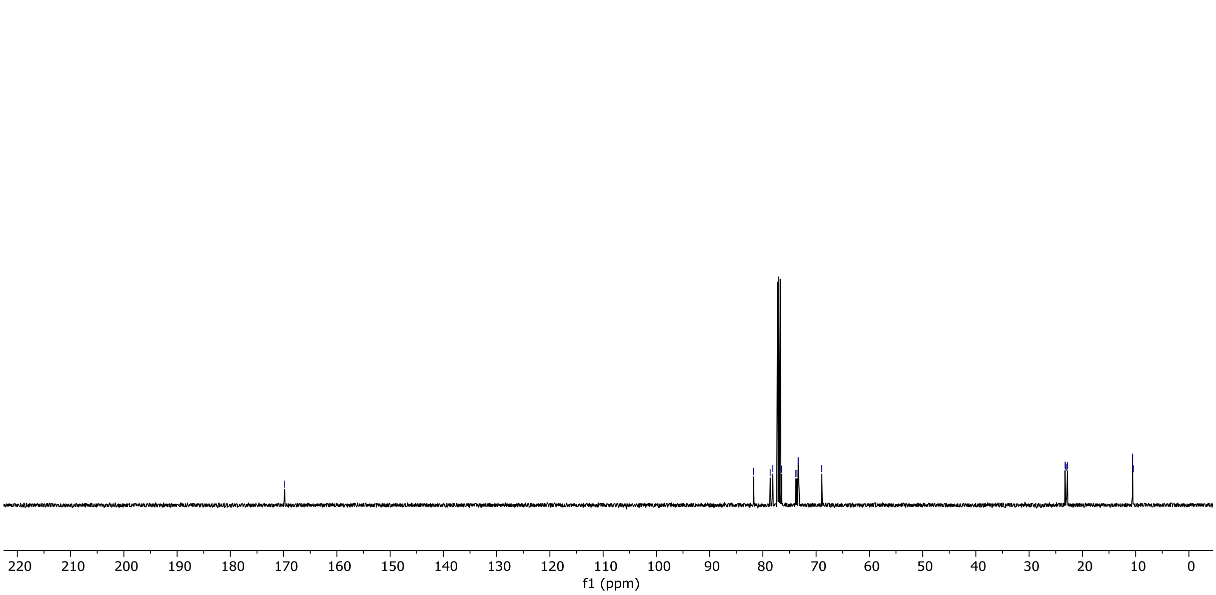
169.79
81.77
78.63
78.13
76.47
73.81
73.59
73.37
73.37
68.94
23.24
23.20
22.92
22.82
10.60
10.58
10.57
10.43
220 210 200 190 180 170 160 150 140 130 120 110 100 90 80 70 60 50 40 30 20 10 0
f1 (ppm)

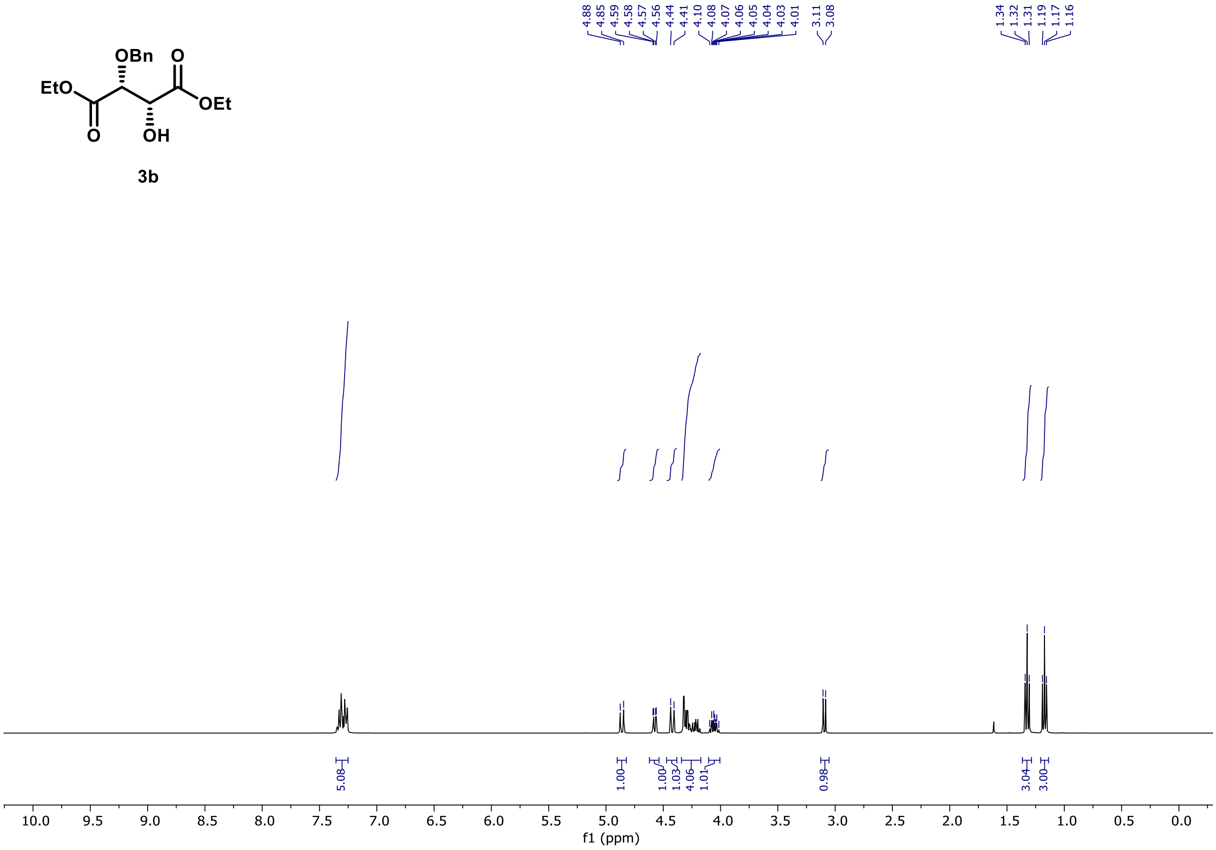
OBn
O
EtO
OEt
O
OH
3b
4.88
4.85
4.59
4.58
4.57
4.56
4.44
4.41
4.10
4.08
4.07
4.06
4.05
4.04
4.03
4.01
3.11
3.08
1.34
1.32
1.31
1.19
1.17
1.16
5.08
1.00
1.00
1.03
4.06
1.01
0.98
3.04
3.00
10.0 9.5 9.0 8.5 8.0 7.5 7.0 6.5 6.0 5.5 5.0 4.5 4.0 3.5 3.0 2.5 2.0 1.5 1.0 0.5 0.0
f1 (ppm)

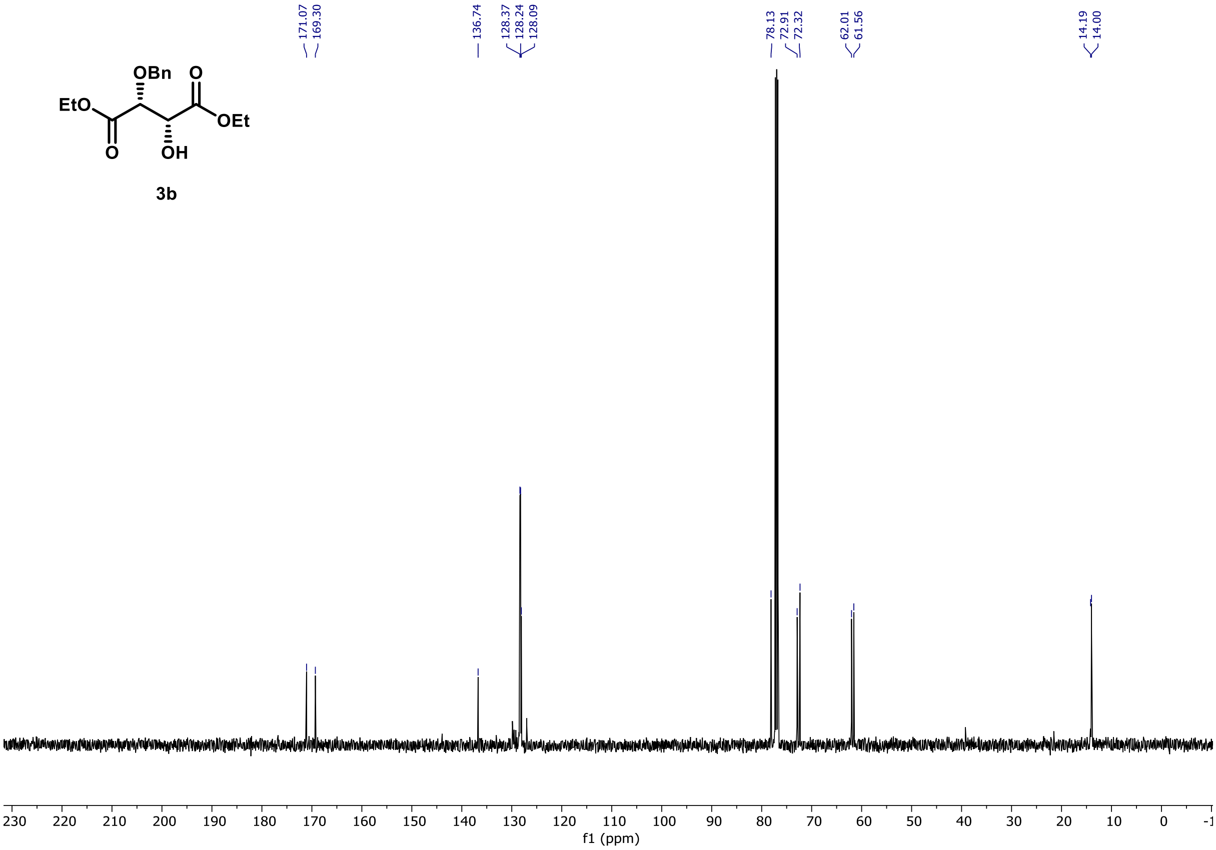
OBn
O
EtO
OEt
O
OH
3b
171.07
169.30
136.74
128.37
128.24
128.09
78.13
72.91
72.32
62.01
61.56
14.19
14.00
230 220 210 200 190 180 170 160 150 140 130 120 110 100 90 80 70 60 50 40 30 20 10 0 -1
f1 (ppm)

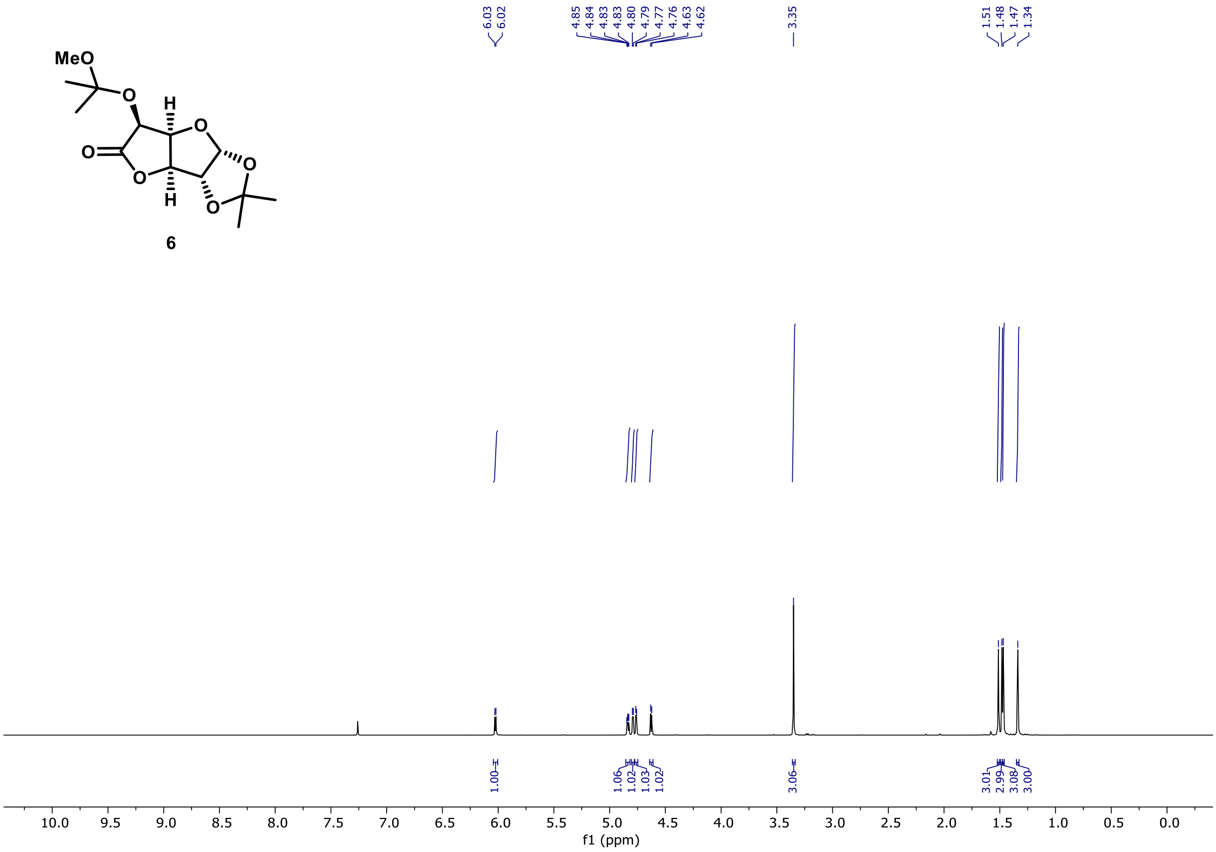
MeO
H
O
O
O
O
H
O
O
6.03
6.02
4.85
4.84
4.83
4.83
4.80
4.79
4.77
4.76
4.63
4.62
3.35
1.51
1.48
1.47
1.34
1.00
1.06
1.02
1.03
1.02
3.06
3.01
2.99
3.08
3.00
10.0 9.5 9.0 8.5 8.0 7.5 7.0 6.5 6.0 5.5 5.0 4.5 4.0 3.5 3.0 2.5 2.0 1.5 1.0 0.5 0.0
f1 (ppm)

6

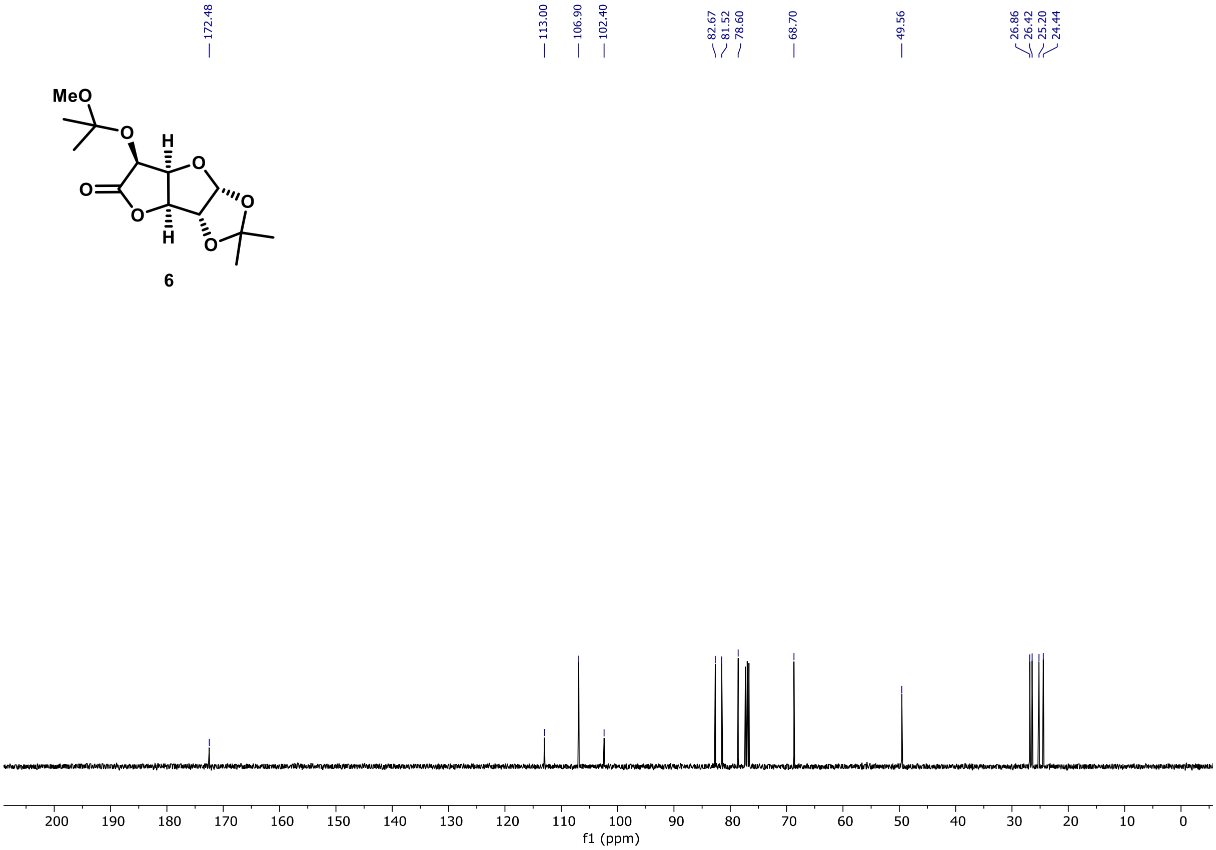
MeO
H
O
O
O
O
H
O
O
172.48
113.00
106.90
102.40
82.67
81.52
78.60
68.70
49.56
26.86
26.42
25.20
24.44
200 190 180 170 160 150 140 130 120 110 100 90 80 70 60 50 40 30 20 10 0
f1 (ppm)

6

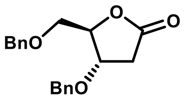

2a

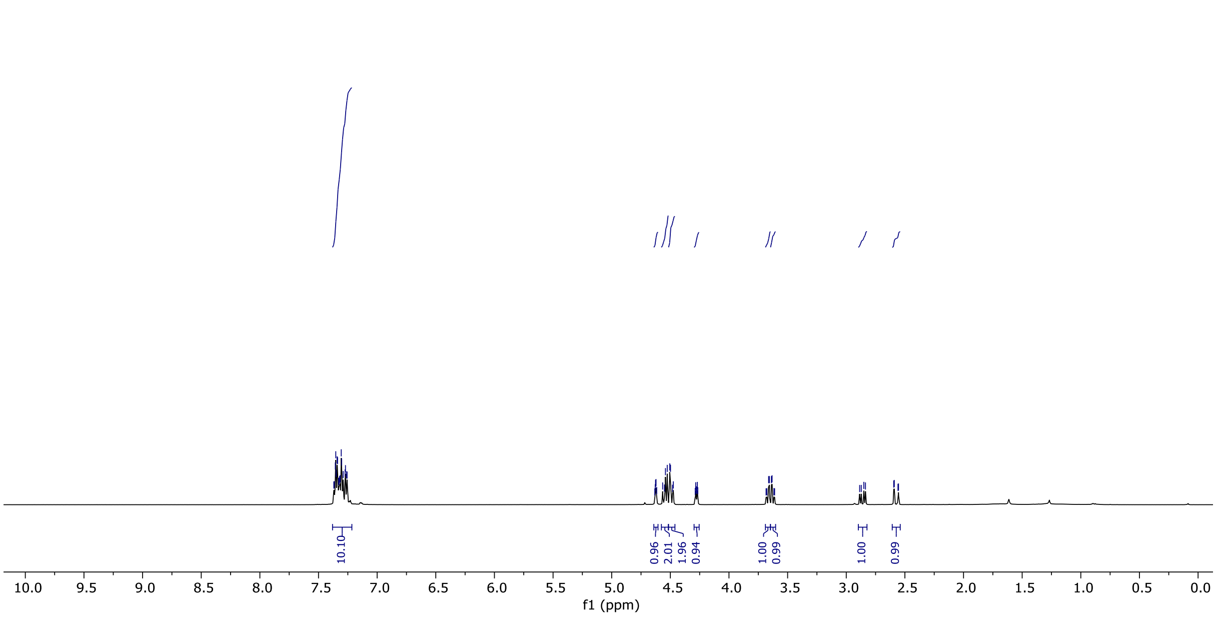
7.37 7.37 7.36 7.35 7.35 7.34 7.34 7.33 7.32 7.32 7.32 7.31 7.30 7.29 7.27 7.27 7.26 4.63 4.63 4.62 4.62 4.57 4.55 4.54 4.53 4.50 4.50 4.48 4.47 4.29 4.28 4.28 4.27 4.27 3.68 3.68 3.66 3.64 3.63 3.62 3.61 2.89 2.87 2.85 2.84 2.60 2.59 2.56 2.55
10.10
0.96 2.01 1.96 0.94
1.00 0.99
1.00
0.99
10.0 9.5 9.0 8.5 8.0 7.5 7.0 6.5 6.0 5.5 5.0 4.5 4.0 3.5 3.0 2.5 2.0 1.5 1.0 0.5 0.0
f1 (ppm)

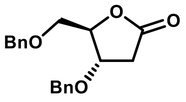

2a

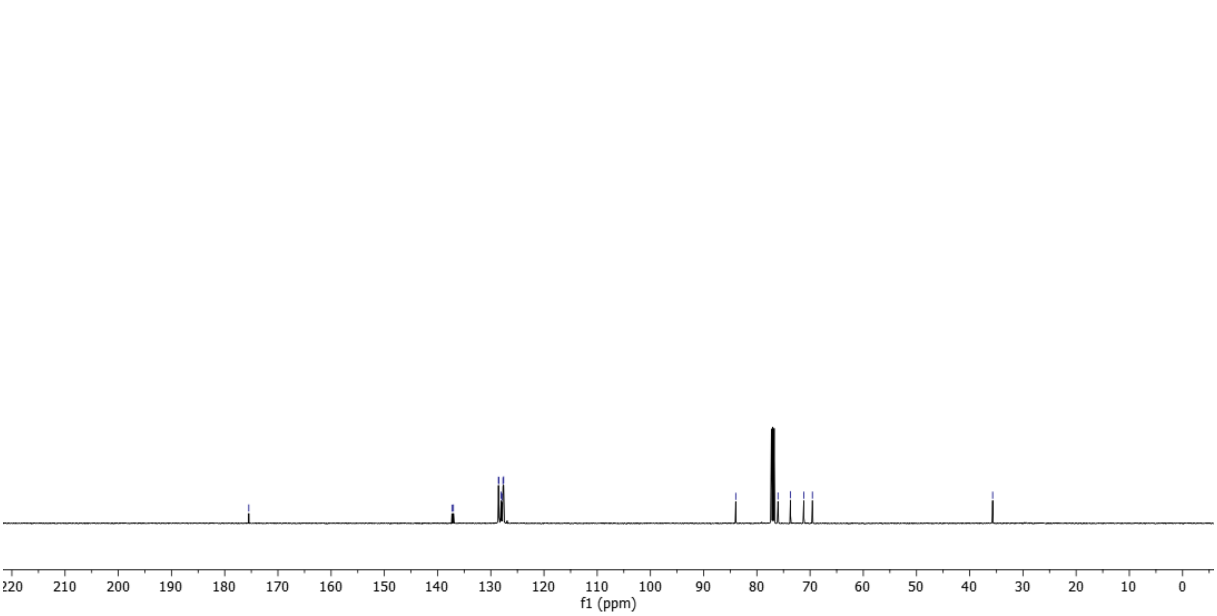
175.48
137.30 137.01 128.56 128.50 128.05 127.92 127.71 127.58
83.97
76.03 73.68 71.18 69.56
35.69
220 210 200 190 180 170 160 150 140 130 120 110 100 90 80 70 60 50 40 30 20 10 0
f1 (ppm)

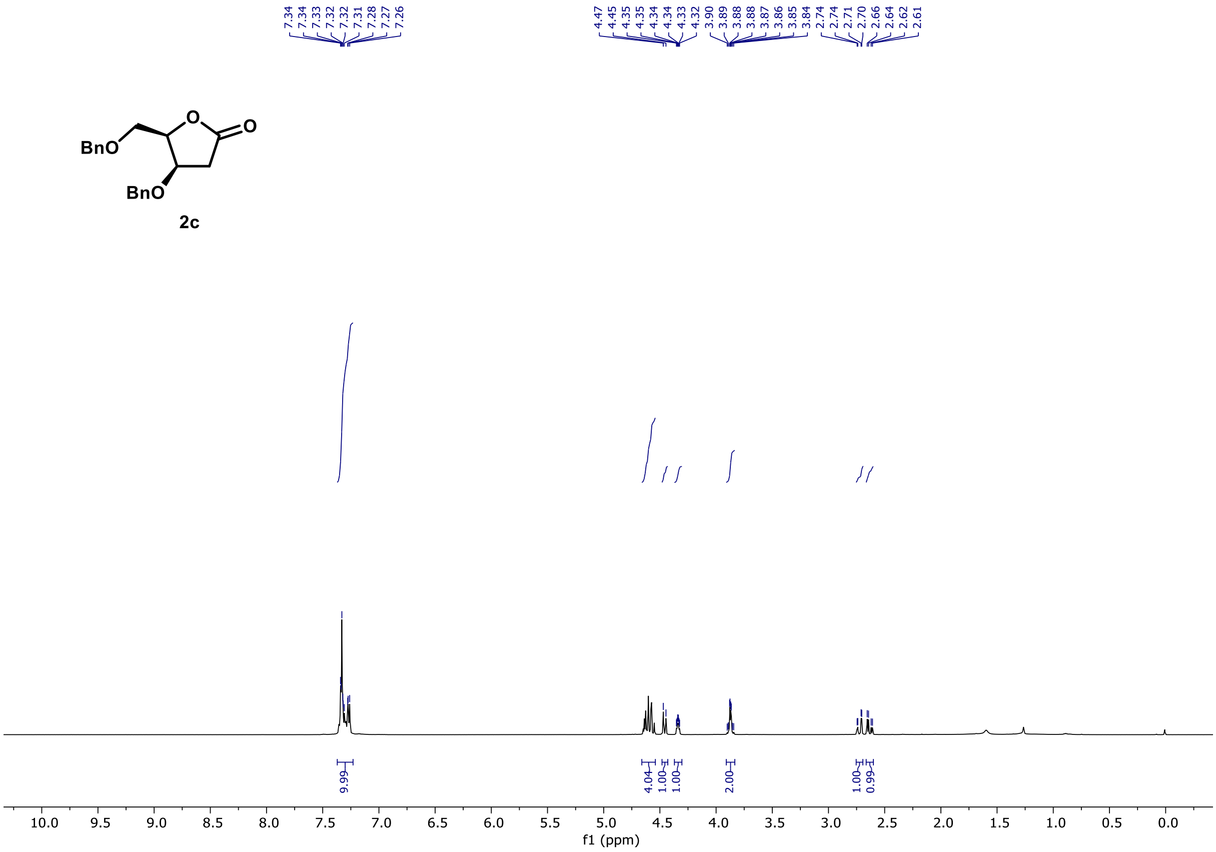
7.34 7.34 7.33 7.32 7.32 7.31 7.28 7.27 7.26
4.47 4.45 4.35 4.35 4.34 4.34 4.33 4.32 3.90 3.89 3.88 3.88 3.87 3.86 3.85 3.84 2.74 2.74 2.71 2.70 2.66 2.64 2.62 2.61
BnO
O
O
BnO
2c
9.99
4.04 1.00 1.00
2.00
1.00 0.99
10.0 9.5 9.0 8.5 8.0 7.5 7.0 6.5 6.0 5.5 5.0 4.5 4.0 3.5 3.0 2.5 2.0 1.5 1.0 0.5 0.0
f1 (ppm)

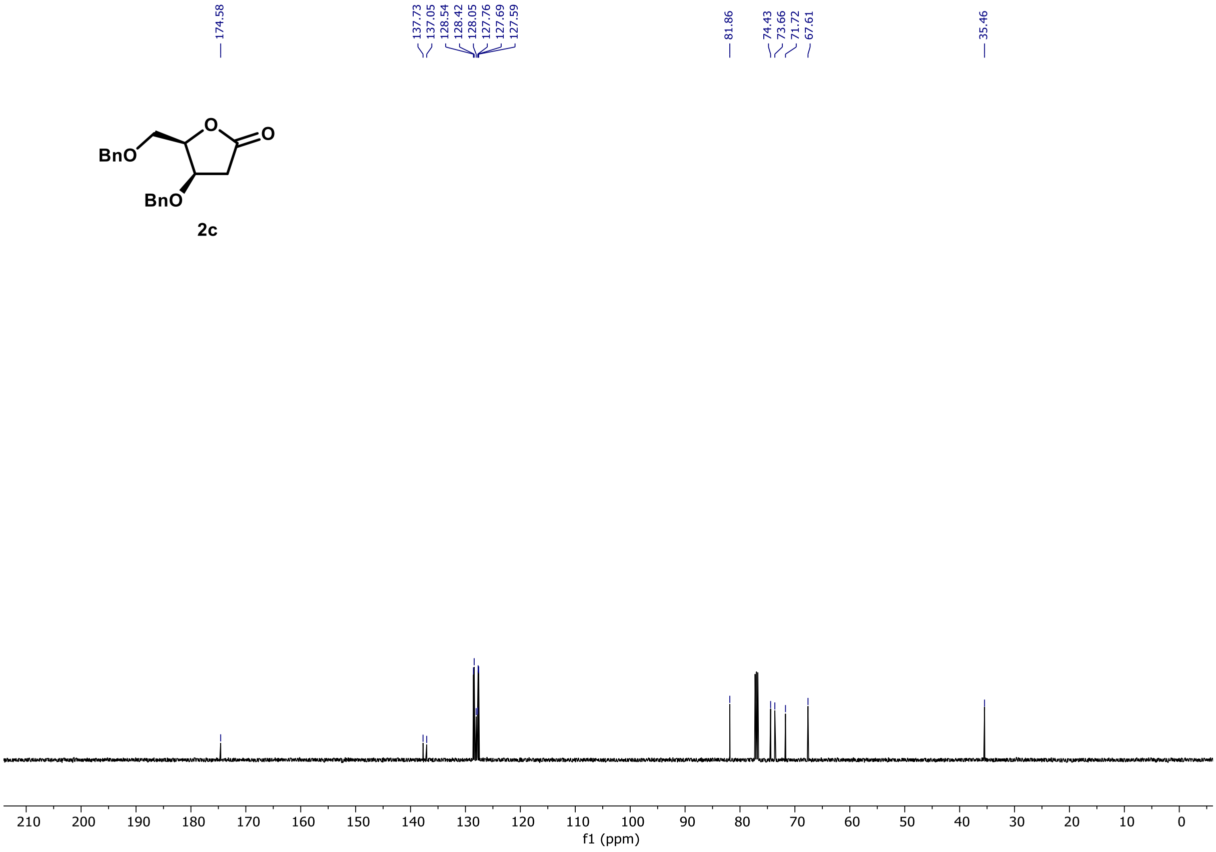
174.58
137.73 137.05 128.54 128.42 128.05 127.76 127.69 127.59
81.86
74.43 73.66 71.72 67.61
35.46
BnO
O
O
BnO
2c
210 200 190 180 170 160 150 140 130 120 110 100 90 80 70 60 50 40 30 20 10 0
f1 (ppm)

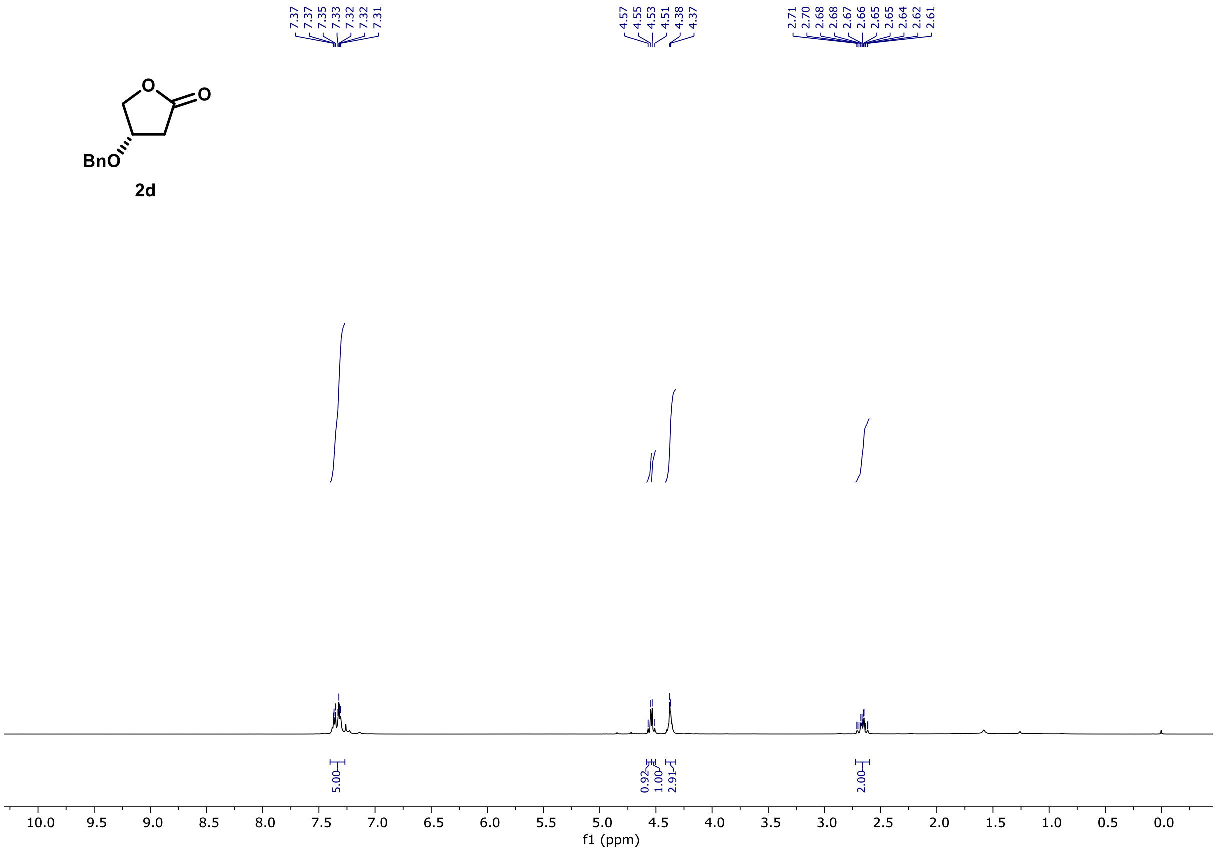
7.37
7.37
7.35
7.33
7.32
7.32
7.31
4.57
4.55
4.53
4.51
4.38
4.37
2.71
2.70
2.68
2.68
2.67
2.66
2.65
2.65
2.64
2.62
2.61
BnO
O
O
2d
5.00
0.92
1.00
2.91
2.00
10.0 9.5 9.0 8.5 8.0 7.5 7.0 6.5 6.0 5.5 5.0 4.5 4.0 3.5 3.0 2.5 2.0 1.5 1.0 0.5 0.0
f1 (ppm)

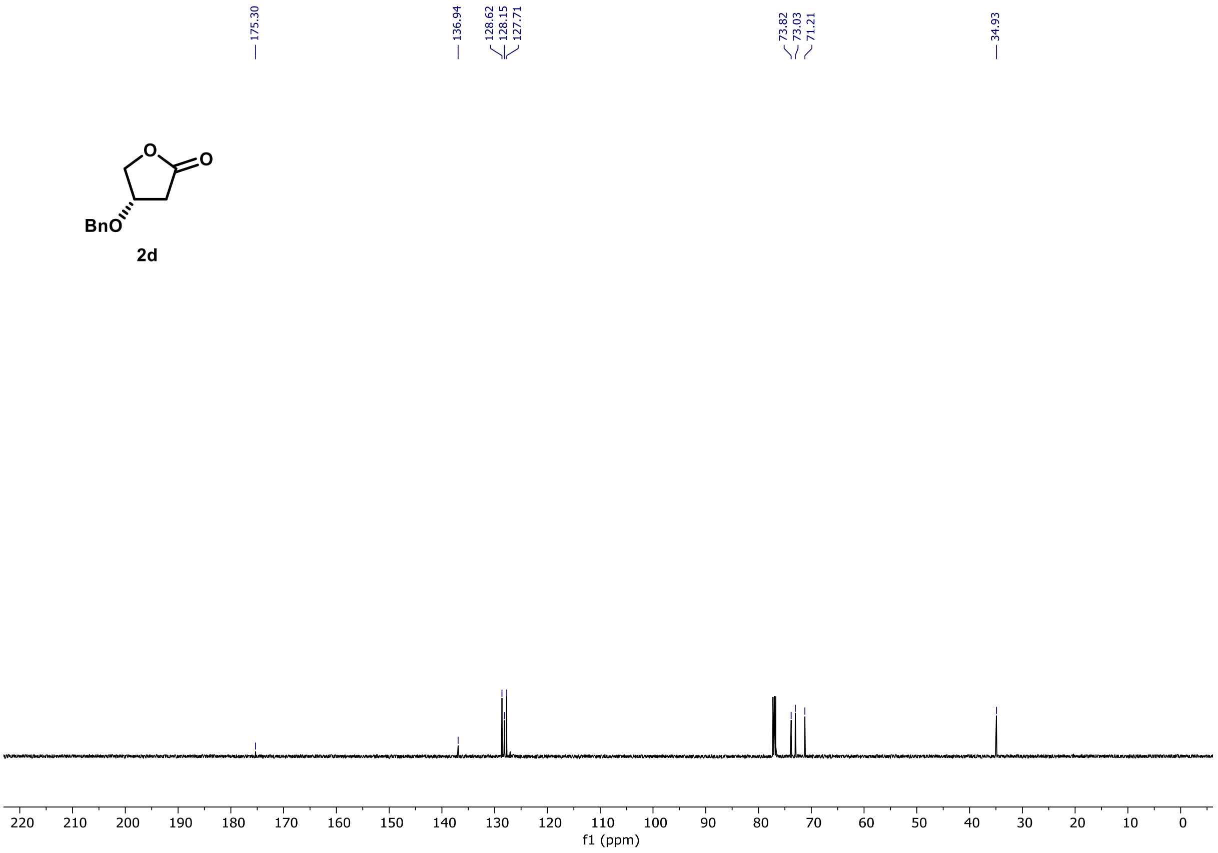
175.30
136.94
128.62
128.15
127.71
73.82
73.03
71.21
34.93
BnO
O
O
2d
220 210 200 190 180 170 160 150 140 130 120 110 100 90 80 70 60 50 40 30 20 10 0
f1 (ppm)

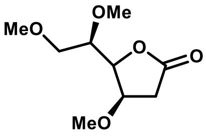
MeO
OMe
O
O
MeO

2e

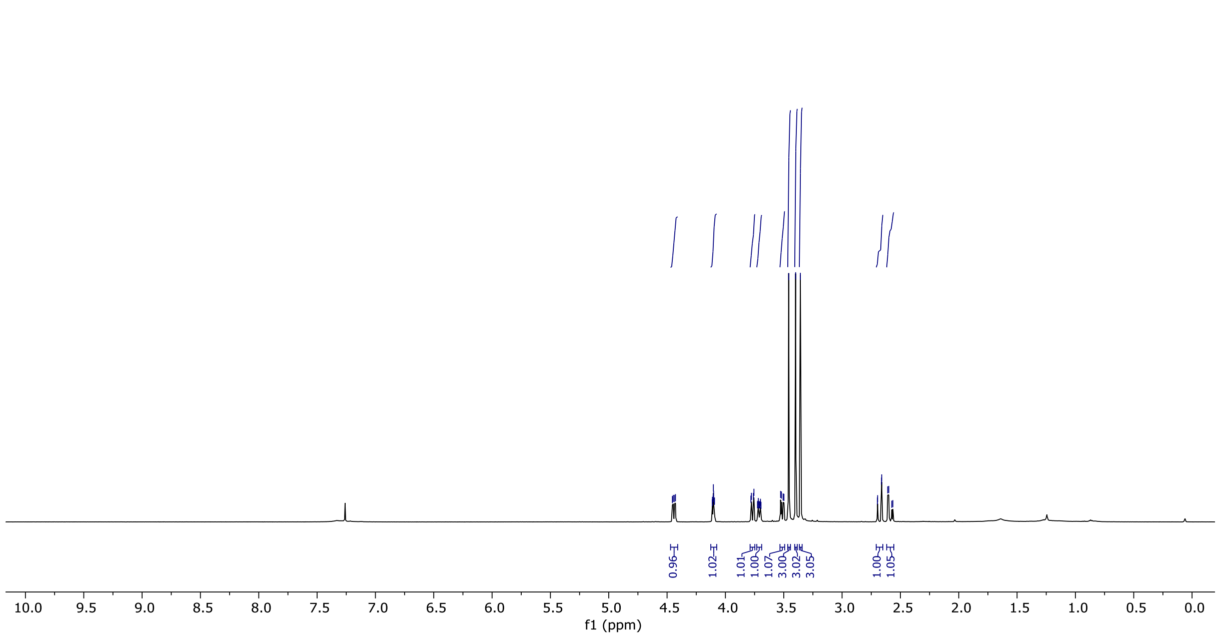
4.45
4.45
4.44
4.43
4.11
4.11
4.11
4.10
4.10
4.10
4.09
3.78
3.78
3.76
3.76
3.73
3.72
3.72
3.71
3.71
3.70
3.70
3.70
3.53
3.52
3.51
3.50
3.46
3.40
3.36
2.70
2.70
2.66
2.66
2.61
2.60
2.57
2.56
0.96
1.02
1.01
1.00
1.07
3.00
3.02
3.05
1.00
1.05
10.0 9.5 9.0 8.5 8.0 7.5 7.0 6.5 6.0 5.5 5.0 4.5 4.0 3.5 3.0 2.5 2.0 1.5 1.0 0.5 0.0
f1 (ppm)

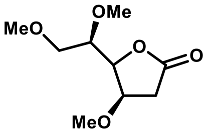
MeO
OMe
O
O
MeO

2e

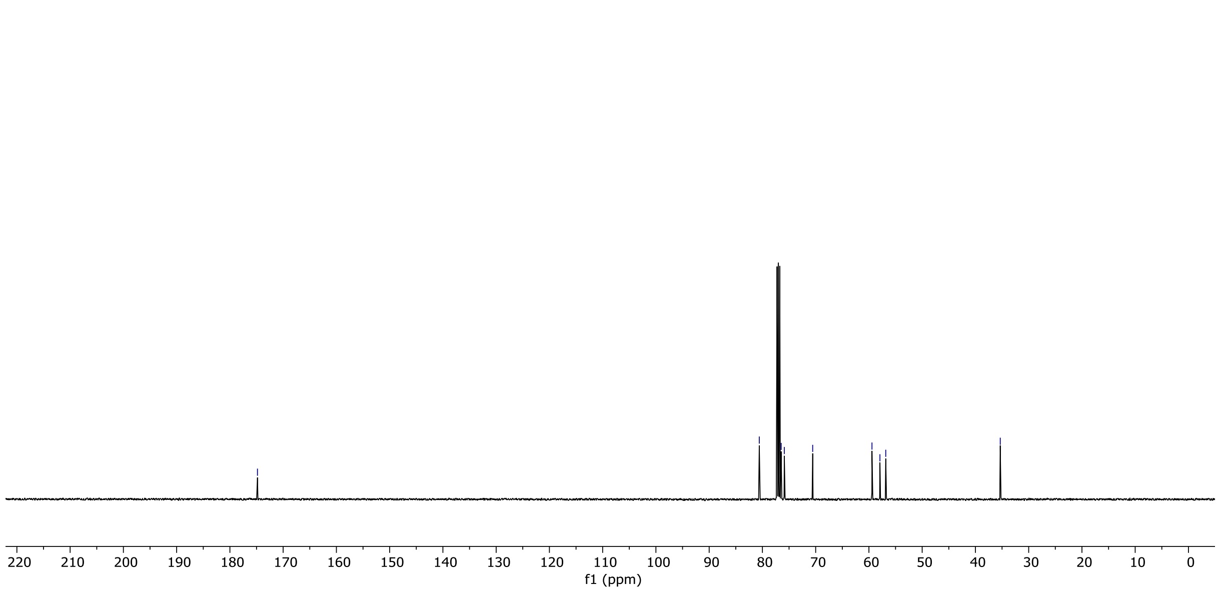
174.82
80.59
76.48
75.89
70.56
59.43
57.93
56.84
35.34
220 210 200 190 180 170 160 150 140 130 120 110 100 90 80 70 60 50 40 30 20 10 0
f1 (ppm)

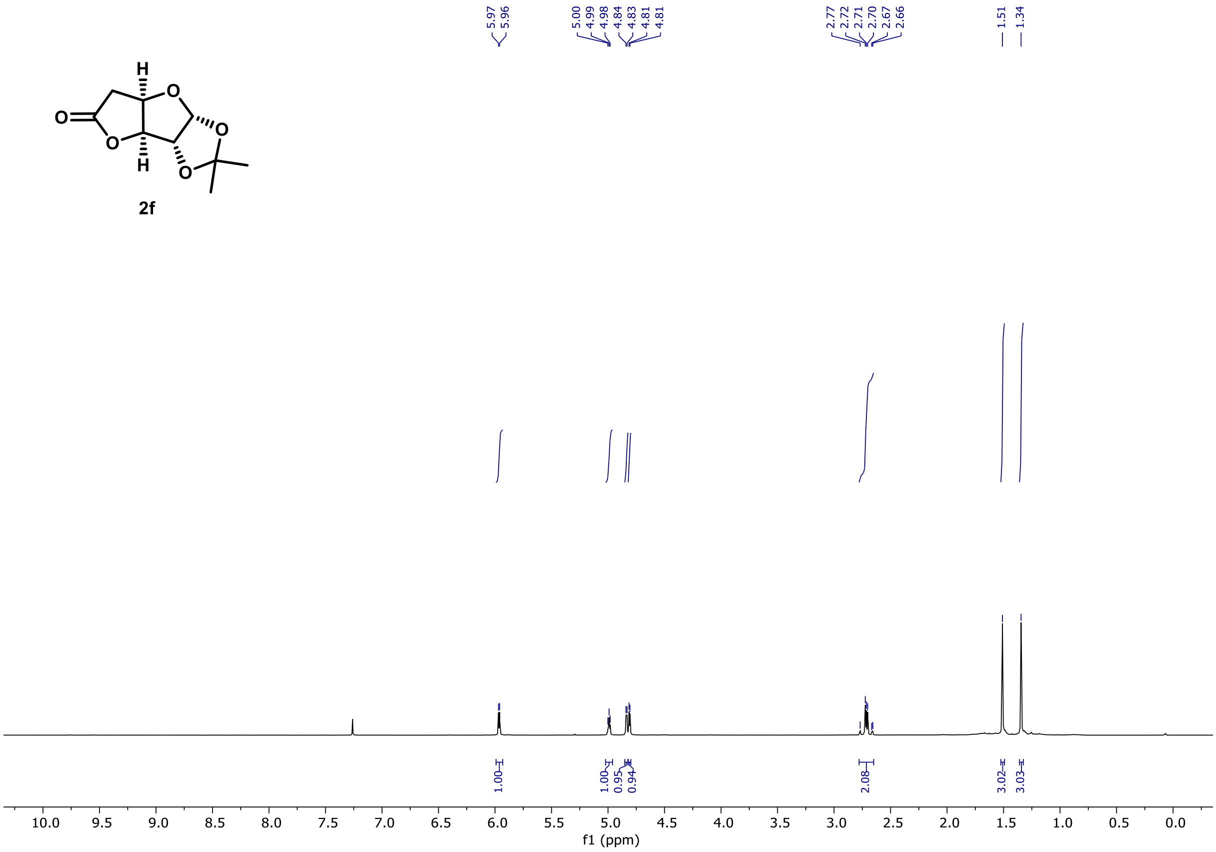
2f
5.97
5.96
5.00
4.99
4.98
4.84
4.83
4.81
4.81
2.77
2.72
2.71
2.70
2.67
2.66
1.51
1.34
1.00
1.00
0.95
0.94
2.08
3.02
3.03
10.0 9.5 9.0 8.5 8.0 7.5 7.0 6.5 6.0 5.5 5.0 4.5 4.0 3.5 3.0 2.5 2.0 1.5 1.0 0.5 0.0
f1 (ppm)

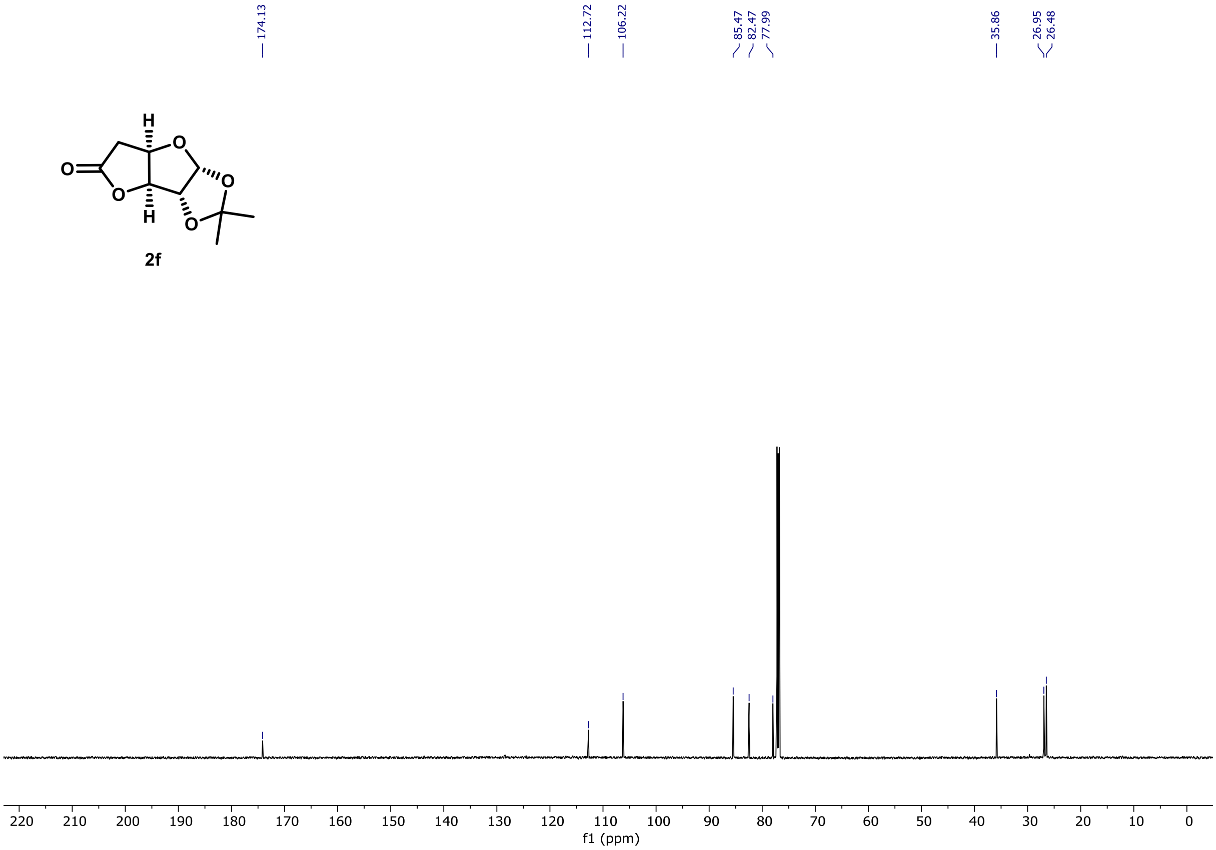
2f
174.13
112.72
106.22
85.47
82.47
77.99
35.86
26.95
26.48
220 210 200 190 180 170 160 150 140 130 120 110 100 90 80 70 60 50 40 30 20 10 0
f1 (ppm)

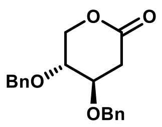

2i

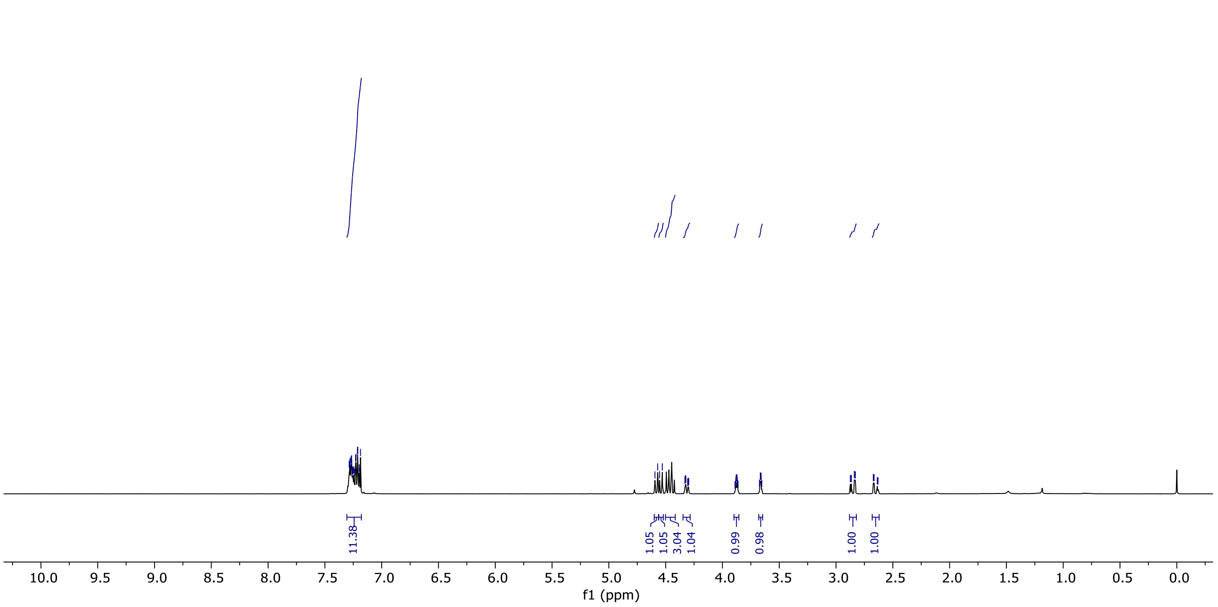

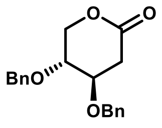

2i

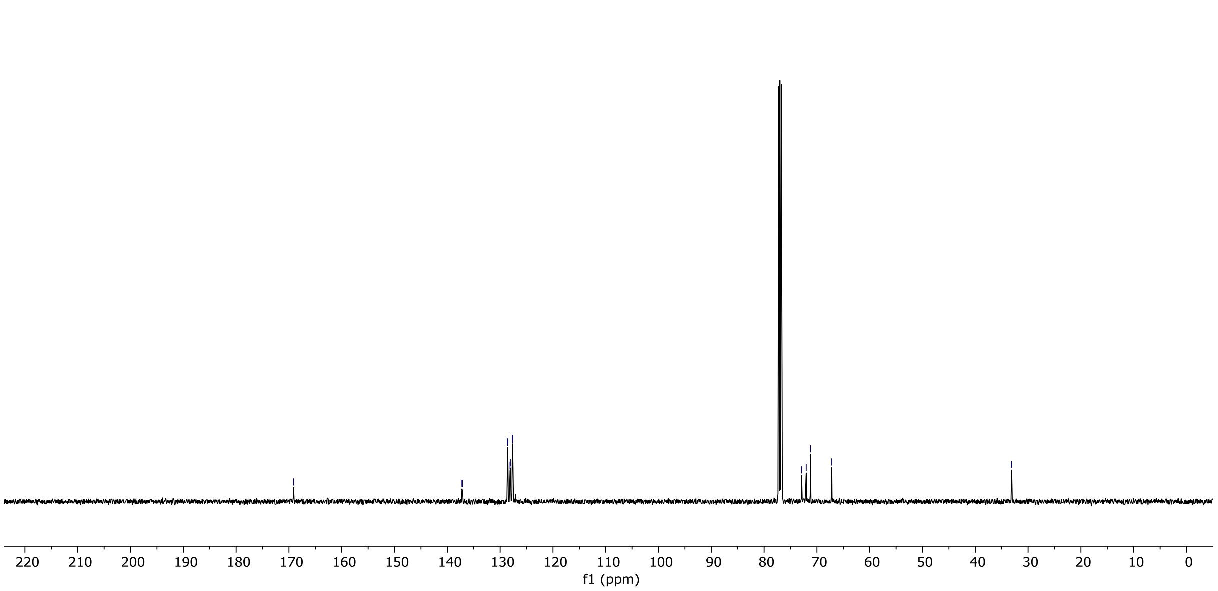

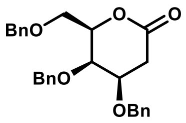
BnO
BnO
OBn
O
O

2j

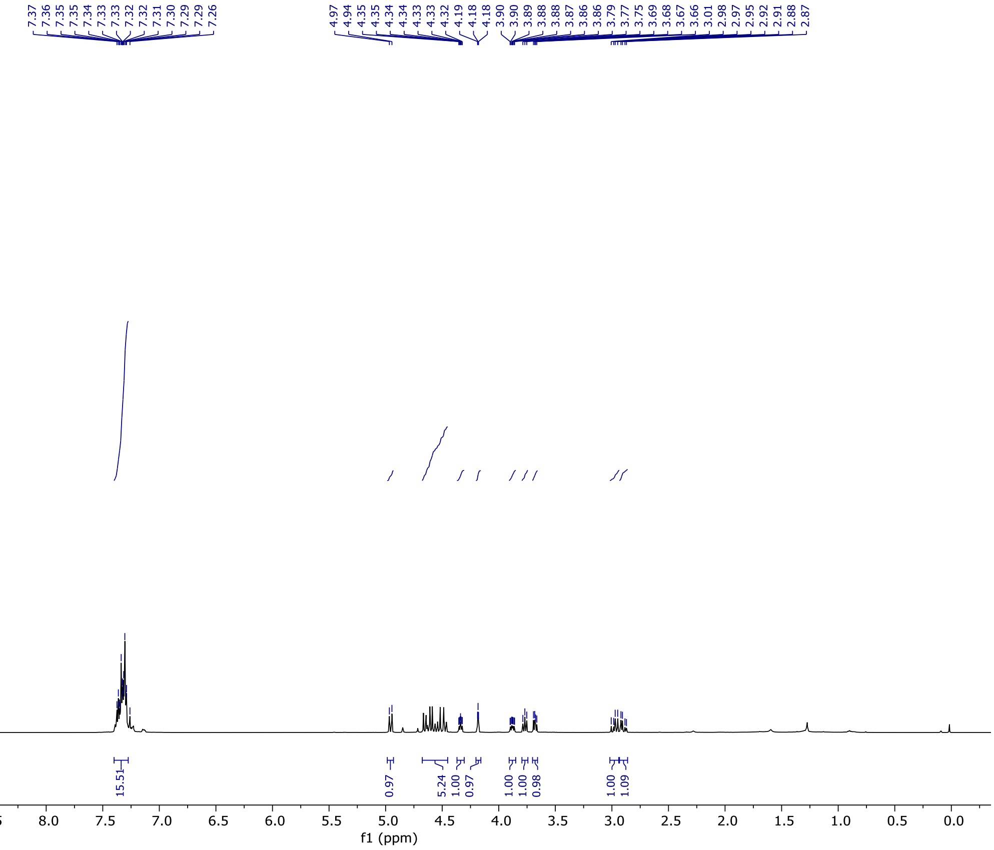
7.37 7.36 7.35 7.35 7.34 7.33 7.33 7.32 7.32 7.31 7.30 7.29 7.29 7.26
4.97 4.94 4.35 4.35 4.34 4.34 4.33 4.33 4.32 4.19 4.18 4.18 3.90 3.90 3.89 3.88 3.88 3.87 3.86 3.86 3.79 3.77 3.75 3.69 3.68 3.67 3.66 3.01 2.98 2.97 2.95 2.92 2.91 2.88 2.87
15.51
0.97
5.24
1.00
0.97
1.00
1.00
0.98
1.00
1.09
10.0 9.5 9.0 8.5 8.0 7.5 7.0 6.5 6.0 5.5 5.0 4.5 4.0 3.5 3.0 2.5 2.0 1.5 1.0 0.5 0.0
f1 (ppm)

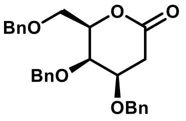
BnO
BnO
OBn
O
O

2j

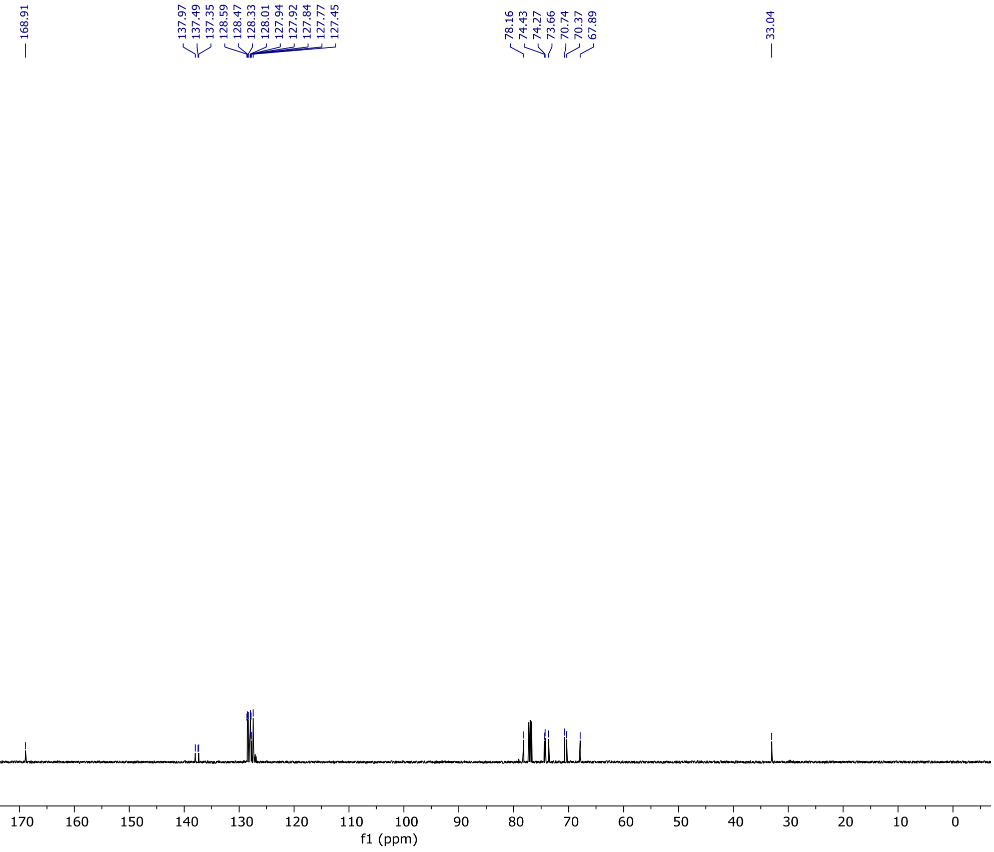
168.91
137.97 137.49 137.35 128.59 128.47 128.33 128.01 127.94 127.92 127.84 127.77 127.45
78.16 74.43 74.27 73.66 70.74 70.37 67.89
33.04
210 200 190 180 170 160 150 140 130 120 110 100 90 80 70 60 50 40 30 20 10 0
f1 (ppm)

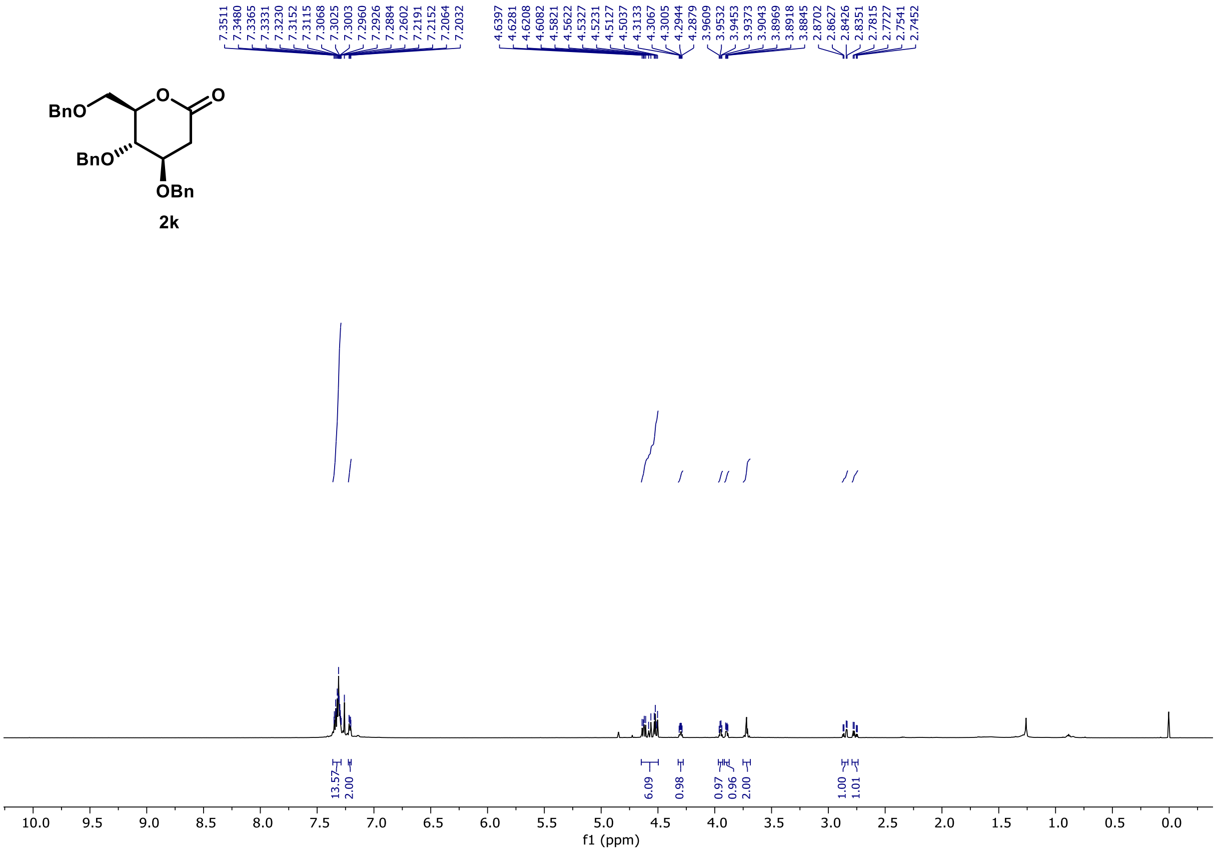
BnO
BnO
OBn
O
O
2k
7.3511 7.3480 7.3365 7.3331 7.3230 7.3152 7.3115 7.3068 7.3025 7.3003 7.2960 7.2926 7.2884 7.2602 7.2191 7.2152 7.2064 7.2032
4.6397 4.6281 4.6208 4.6082 4.5821 4.5622 4.5327 4.5231 4.5127 4.5037 4.3133 4.3067 4.3005 4.2944 4.2879 3.9609 3.9532 3.9453 3.9373 3.9043 3.8969 3.8918 3.8845 2.8702 2.8627 2.8426 2.8351 2.7815 2.7727 2.7541 2.7452
13.57 2.00 6.09 0.98 0.97 0.96 2.00 1.00 1.01
f1 (ppm)

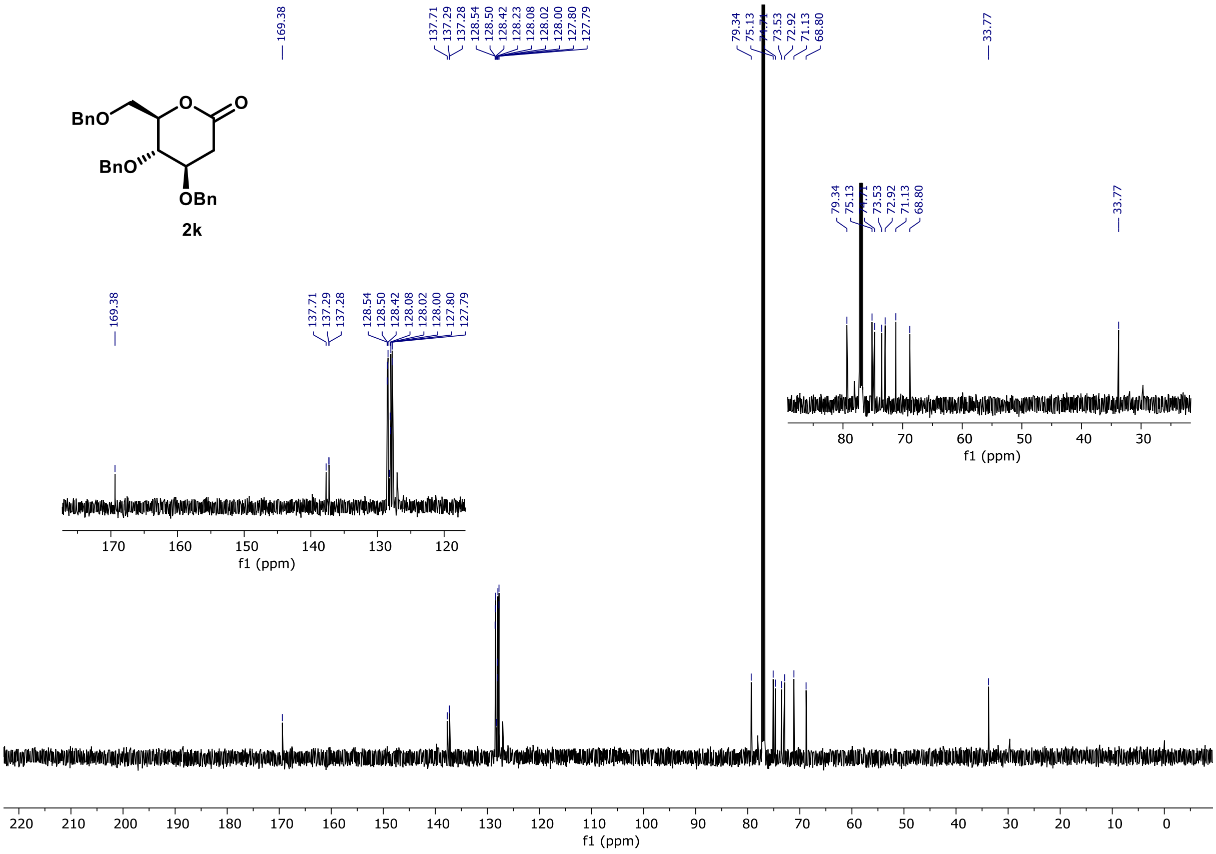
BnO
BnO
OBn
O
O
2k
169.38
137.71 137.29 137.28 128.54 128.50 128.42 128.23 128.08 128.02 128.00 127.80 127.79
79.34 75.13 74.71 73.53 72.92 71.13 68.80
33.77
f1 (ppm)

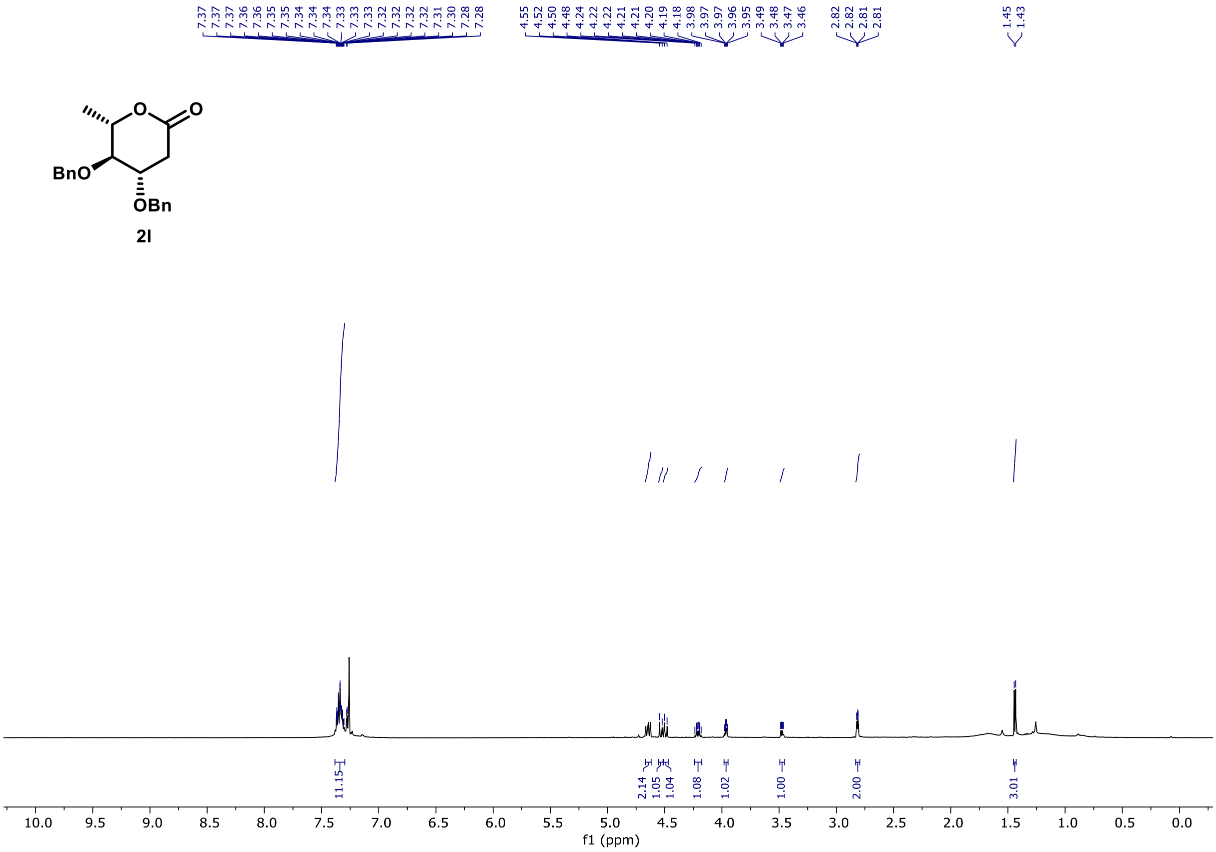

7.37 7.37 7.36 7.36 7.35 7.35 7.34 7.34 7.33 7.33 7.33 7.32 7.32 7.32 7.32 7.31 7.30 7.28 7.28
4.55 4.52 4.50 4.48 4.24 4.22 4.22 4.21 4.21 4.20 4.19 4.18 3.98 3.97 3.97 3.96 3.95 3.49 3.48 3.47 3.46
2.82 2.82 2.81 2.81
1.45 1.43
BnO
OBn
11.15 2.14 1.05 1.04 1.08 1.02 1.00 2.00 3.01
10.0 9.5 9.0 8.5 8.0 7.5 7.0 6.5 6.0 5.5 5.0 4.5 4.0 3.5 3.0 2.5 2.0 1.5 1.0 0.5 0.0
f1 (ppm)

2l

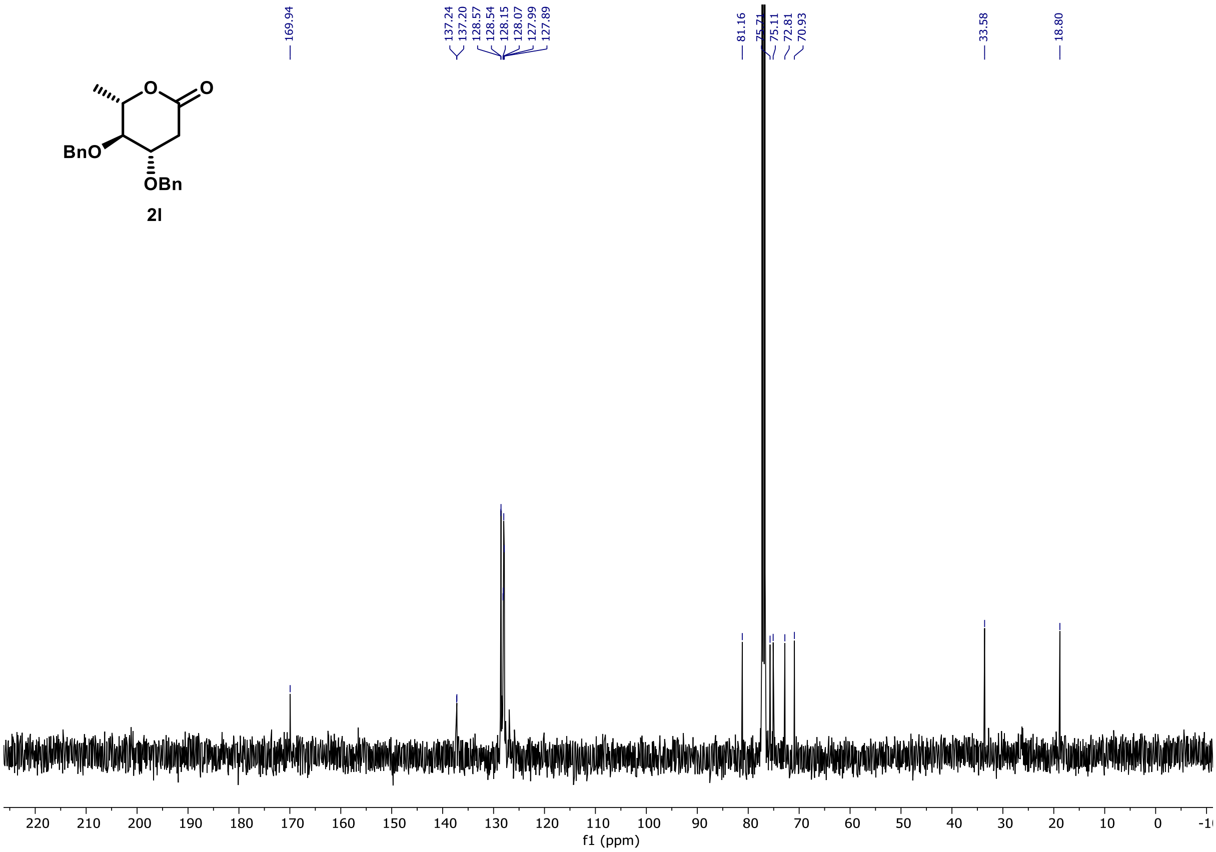

169.94
137.24 137.20 128.57 128.54 128.15 128.07 127.99 127.89
81.16 75.71 75.11 72.81 70.93
33.58
18.80
BnO
OBn
220 210 200 190 180 170 160 150 140 130 120 110 100 90 80 70 60 50 40 30 20 10 0
f1 (ppm)

2l

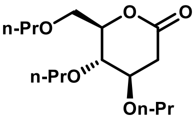
n-PrO
O
O
n-PrO
On-Pr

2m

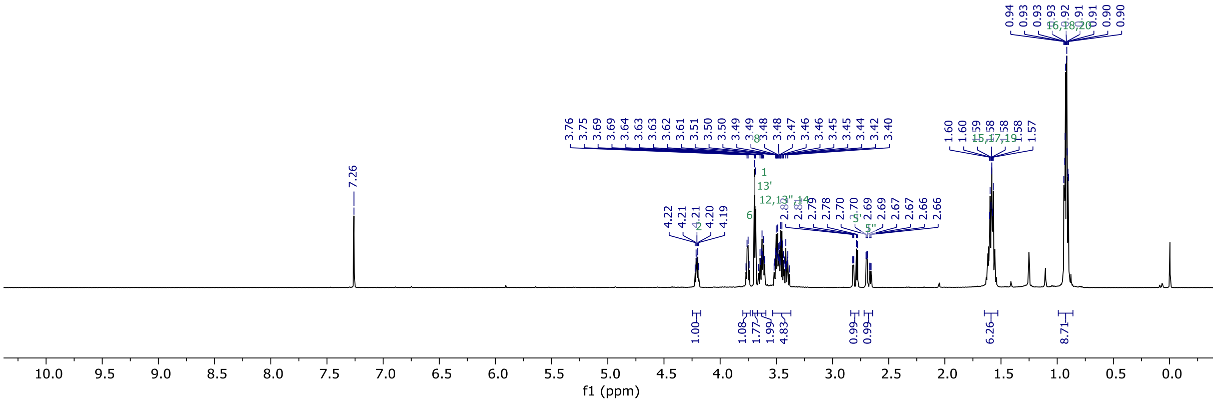
7.26
4.22
4.21
4.21
4.20
4.19
3.76
3.75
3.69
3.69
3.64
3.63
3.63
3.62
3.61
3.51
3.50
3.50
3.49
3.49
3.48
3.48
3.47
3.46
3.46
3.45
3.45
3.44
3.42
3.40
2.82
2.82
2.79
2.78
2.70
2.70
2.69
2.69
2.67
2.67
2.66
2.66
1.60
1.60
1.59
1.58
1.58
1.58
1.57
0.94
0.93
0.93
0.93
0.92
0.91
0.91
0.90
0.90
1.00
1.08
1.77
1.99
4.83
0.99
0.99
6.26
8.71
f1 (ppm)

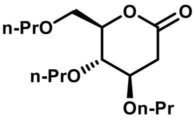
n-PrO
O
O
n-PrO
On-Pr

2m

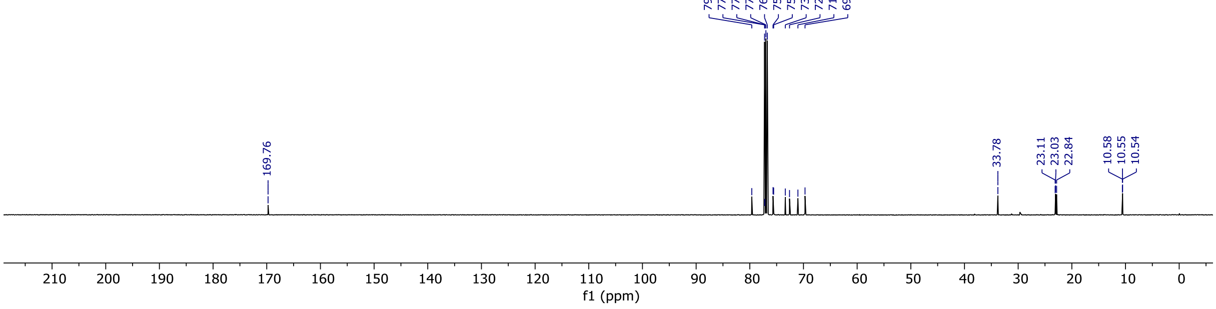
169.76
79.63
77.25
77.20
77.00
76.74
75.67
75.56
73.39
72.60
71.03
69.71
33.78
23.11
23.03
22.84
10.58
10.55
10.54
f1 (ppm)

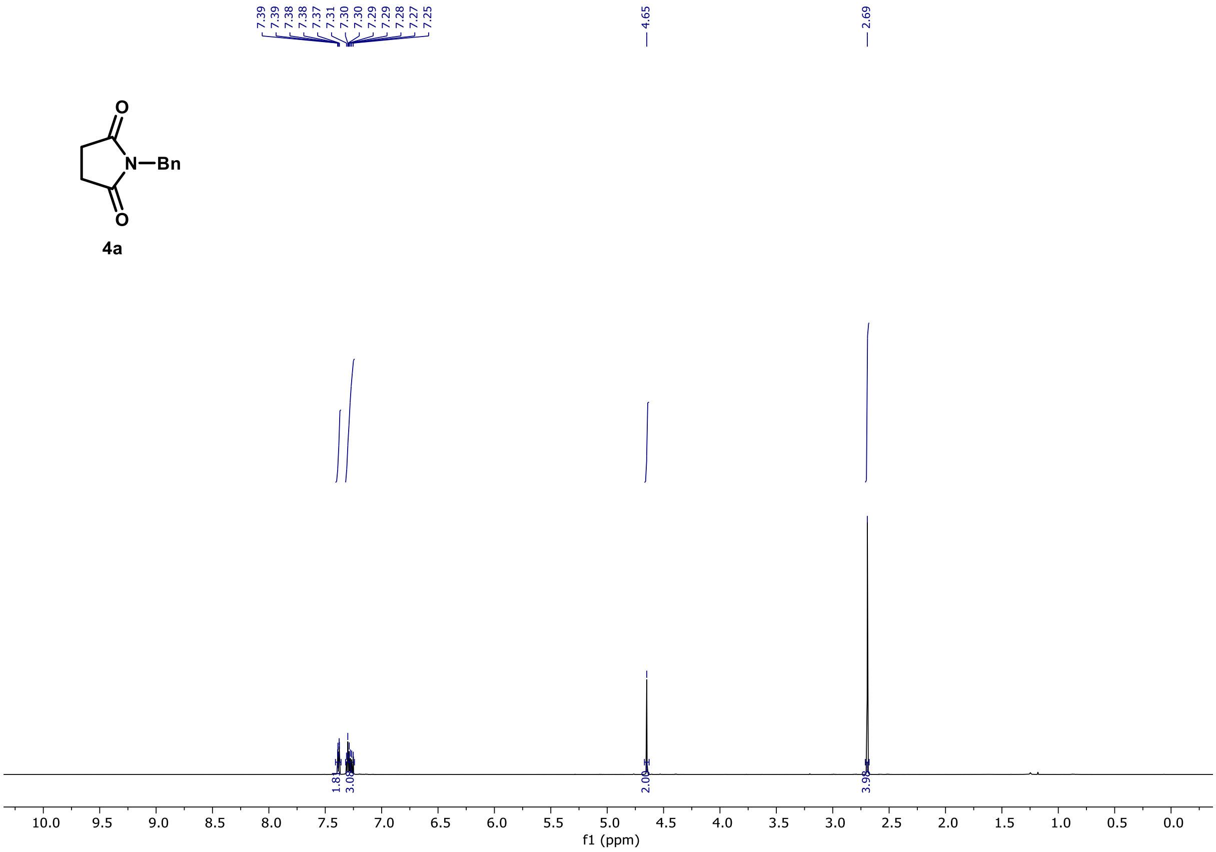
7.39
7.39
7.38
7.38
7.37
7.31
7.30
7.30
7.29
7.29
7.28
7.27
7.25
4.65
2.69
O
N−Bn
O
4a
1.81
3.09
2.00
3.98
10.0 9.5 9.0 8.5 8.0 7.5 7.0 6.5 6.0 5.5 5.0 4.5 4.0 3.5 3.0 2.5 2.0 1.5 1.0 0.5 0.0
f1 (ppm)

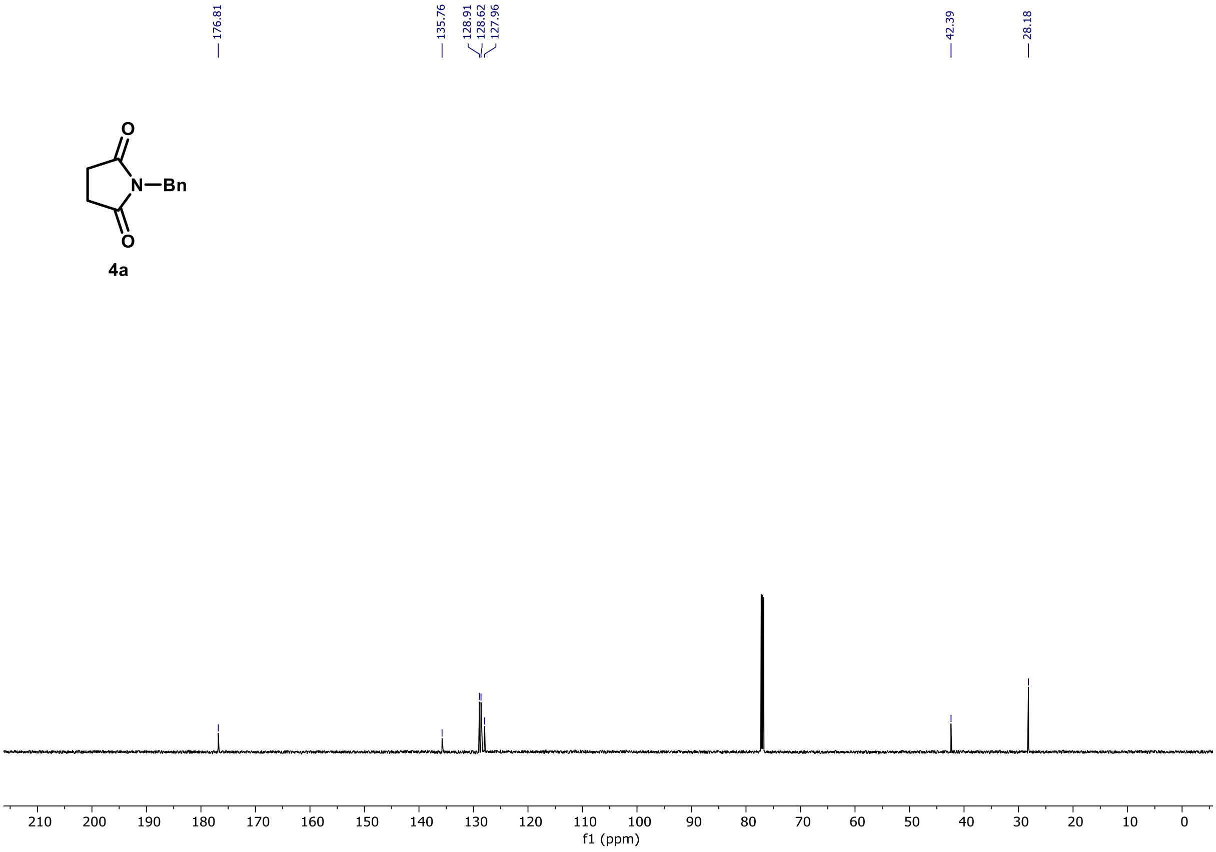
176.81
135.76
128.91
128.62
127.96
42.39
28.18
O
N−Bn
O
4a
210 200 190 180 170 160 150 140 130 120 110 100 90 80 70 60 50 40 30 20 10 0
f1 (ppm)

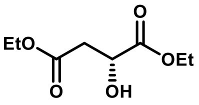
EtO
O
O
OEt
OH

4b

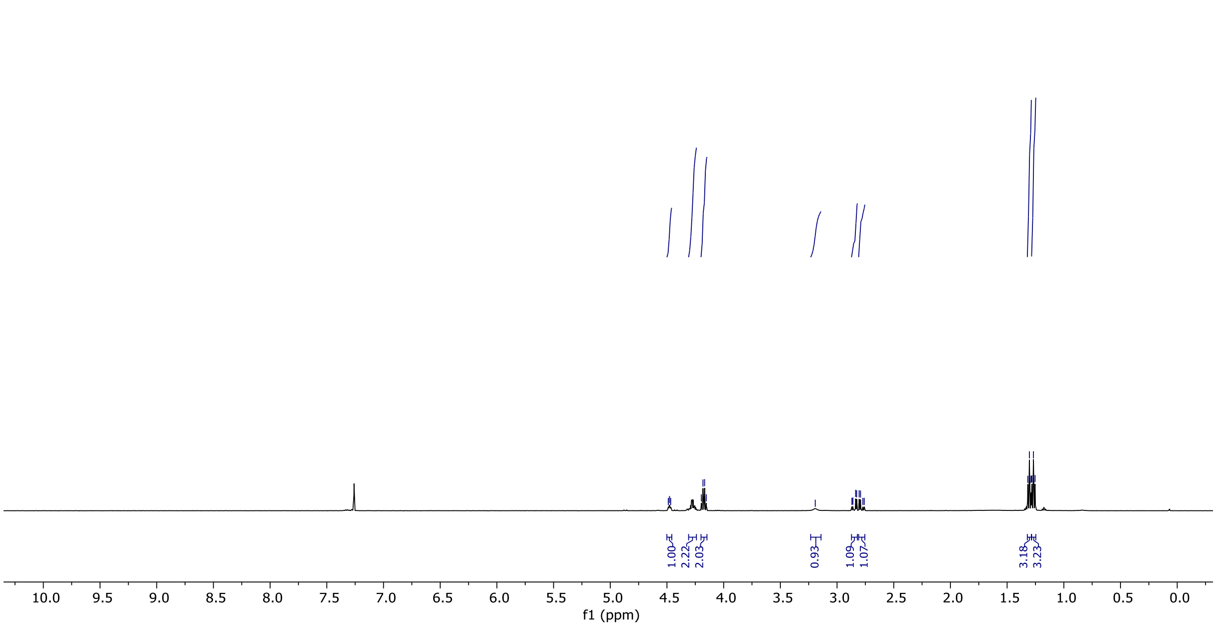
4.49
4.48
4.47
4.20
4.18
4.17
4.15
3.19
2.87
2.86
2.84
2.83
2.80
2.79
2.77
2.76
1.32
1.30
1.29
1.28
1.27
1.25
1.00
2.22
2.03
0.93
1.09
1.07
3.18
3.23
f1 (ppm)

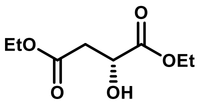
EtO
O
O
OEt
OH

4b

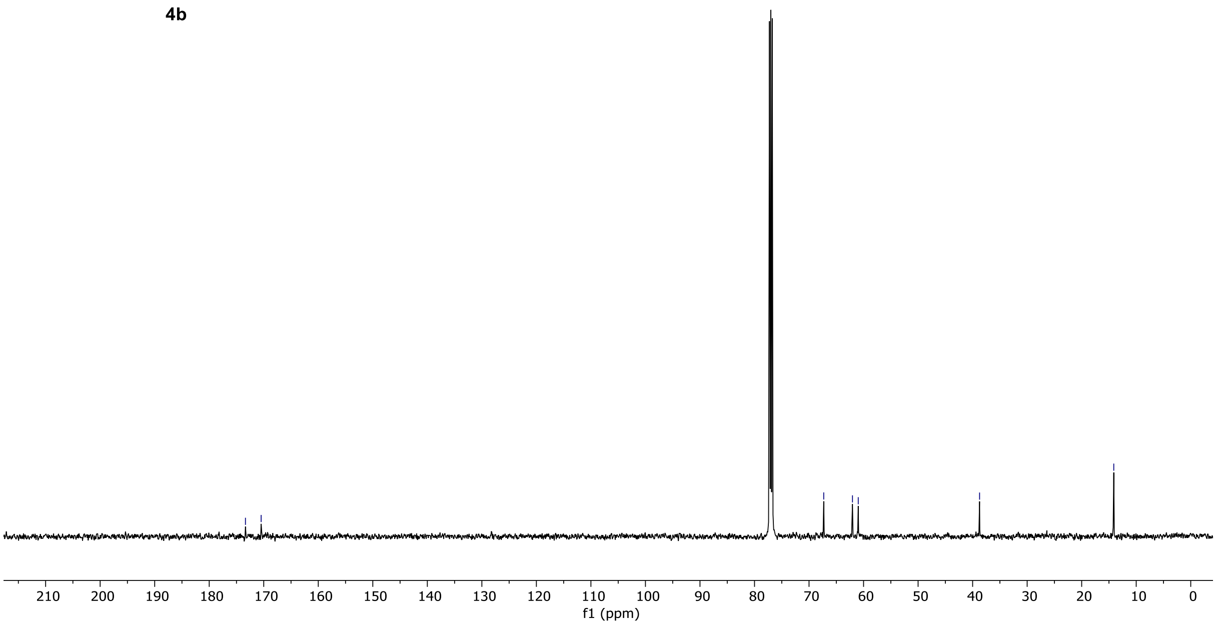
173.36
170.48
67.31
62.03
60.98
38.72
14.12
f1 (ppm)

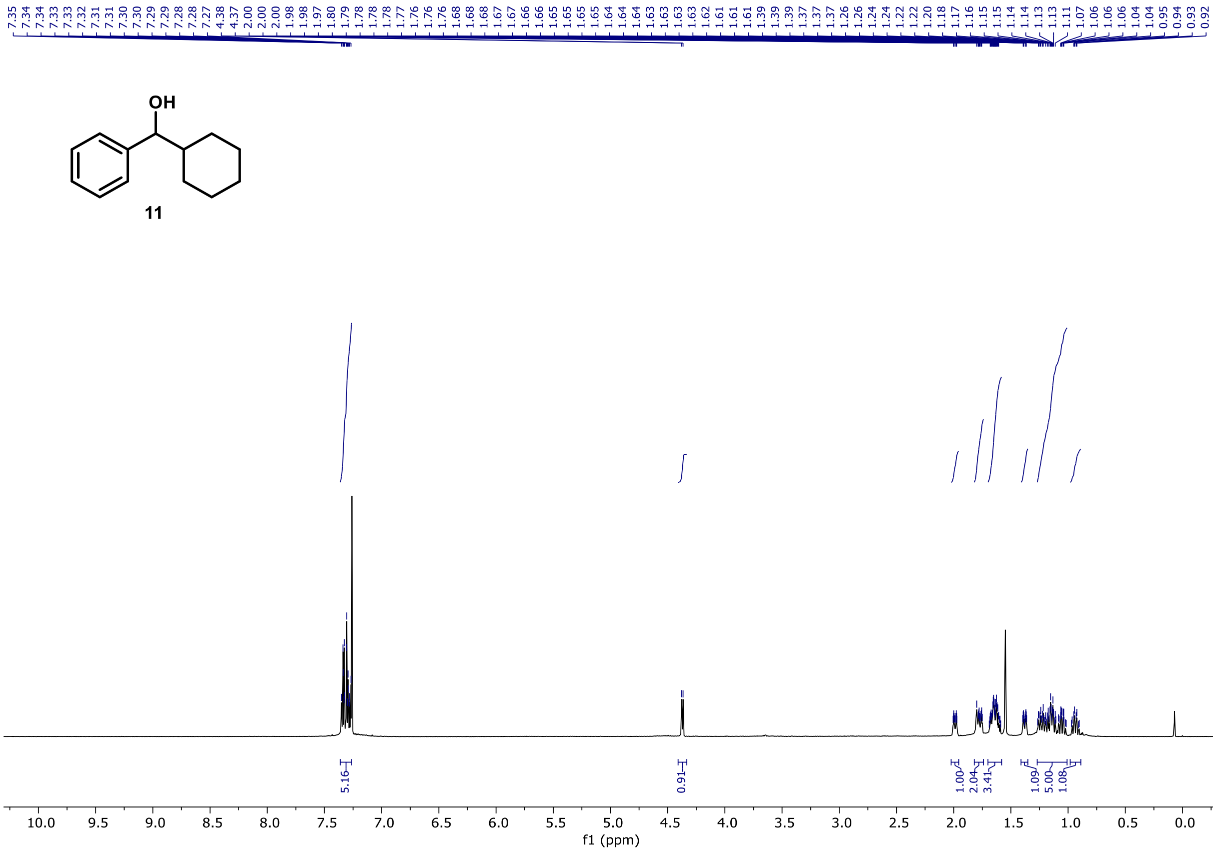
7.35 7.34 7.34 7.33 7.33 7.32 7.31 7.31 7.30 7.30 7.29 7.29 7.28 7.28 7.27 4.38 4.37 2.00 2.00 1.98 1.98 1.97 1.80 1.79 1.78 1.78 1.77 1.76 1.76 1.68 1.68 1.67 1.67 1.66 1.66 1.65 1.65 1.65 1.64 1.64 1.63 1.63 1.63 1.63 1.62 1.61 1.61 1.61 1.39 1.39 1.39 1.37 1.37 1.26 1.26 1.24 1.24 1.22 1.22 1.20 1.18 1.17 1.16 1.15 1.15 1.14 1.14 1.13 1.13 1.11 1.07 1.06 1.06 1.04 1.04 0.95 0.94 0.93 0.92
OH
11
5.16
0.91
1.00
2.04
3.41
1.09
5.00
1.08
10.0 9.5 9.0 8.5 8.0 7.5 7.0 6.5 6.0 5.5 5.0 4.5 4.0 3.5 3.0 2.5 2.0 1.5 1.0 0.5 0.0
f1 (ppm)

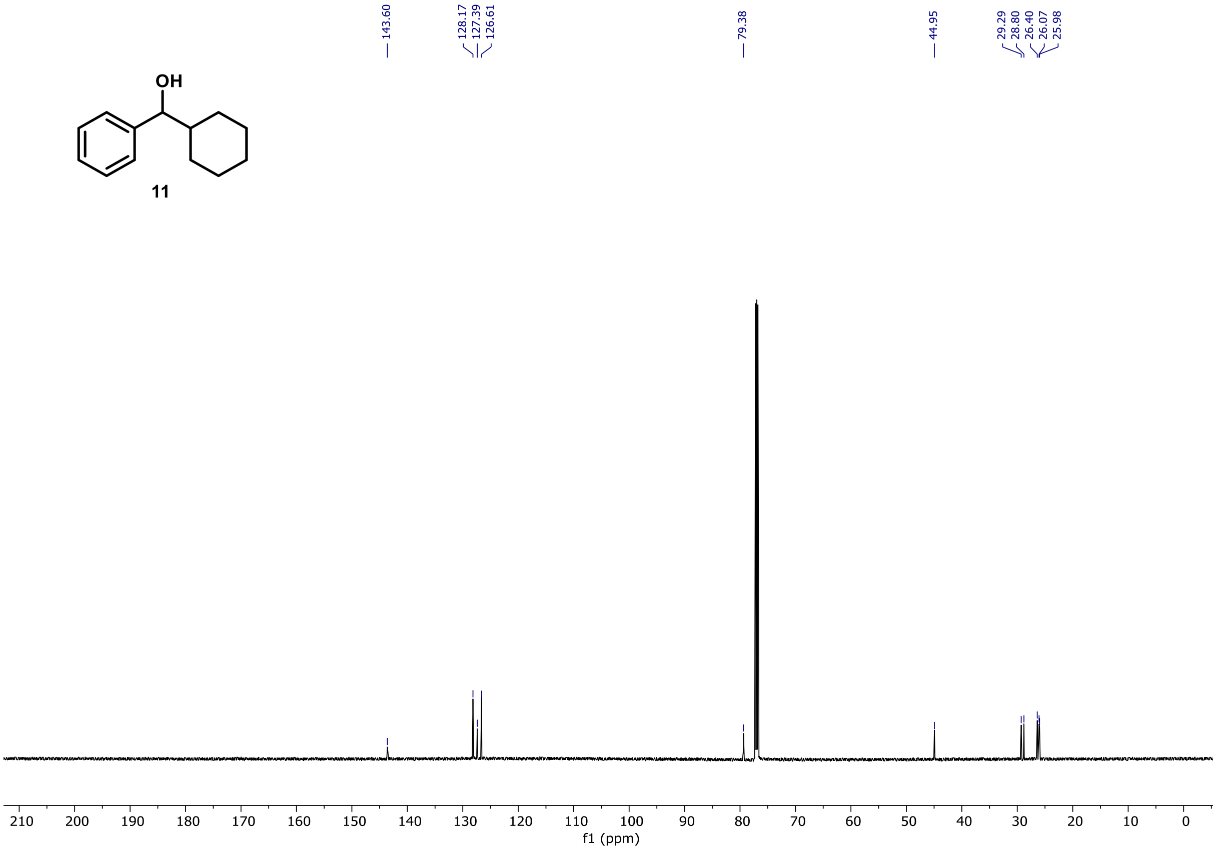
143.60
128.17 127.39 126.61
79.38
44.95
29.29 28.80 26.40 26.07 25.98
OH
11
210 200 190 180 170 160 150 140 130 120 110 100 90 80 70 60 50 40 30 20 10 0
f1 (ppm)

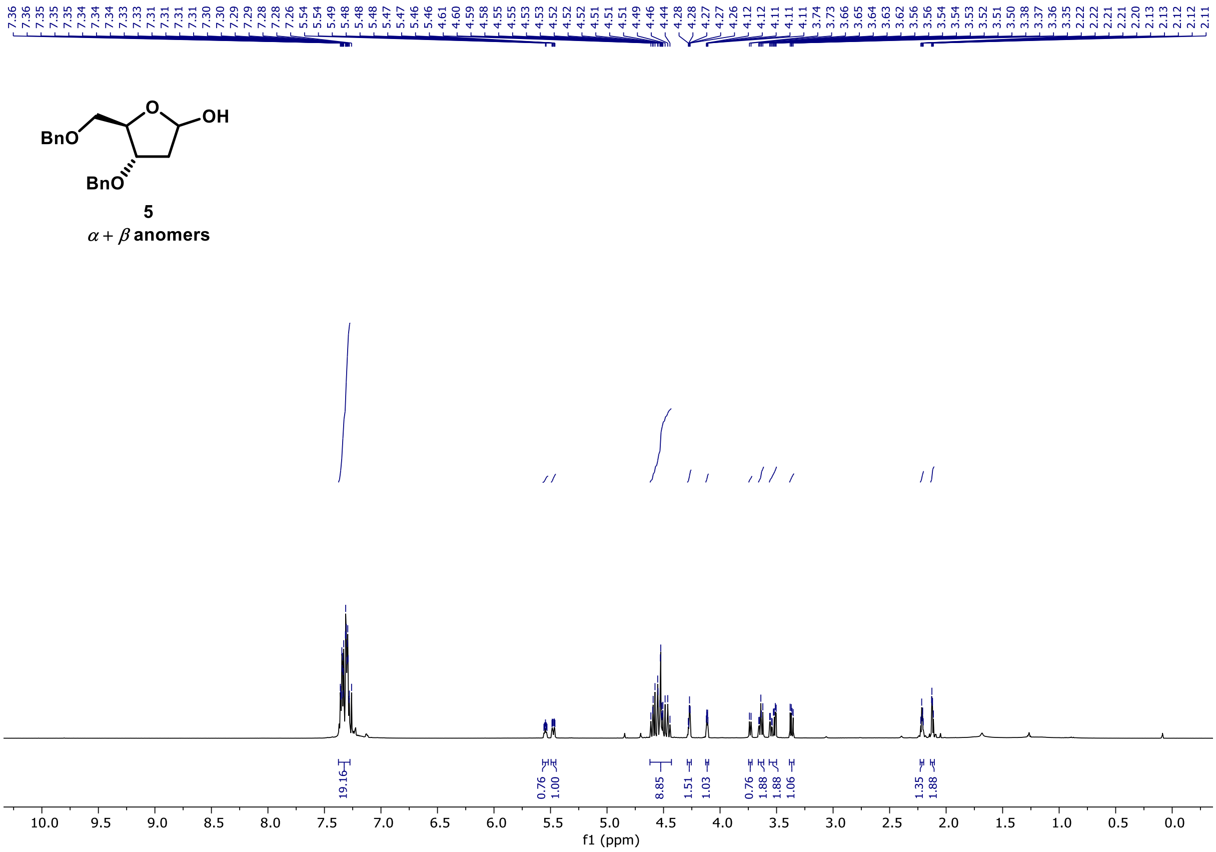

5

$\alpha + \beta$ anomers

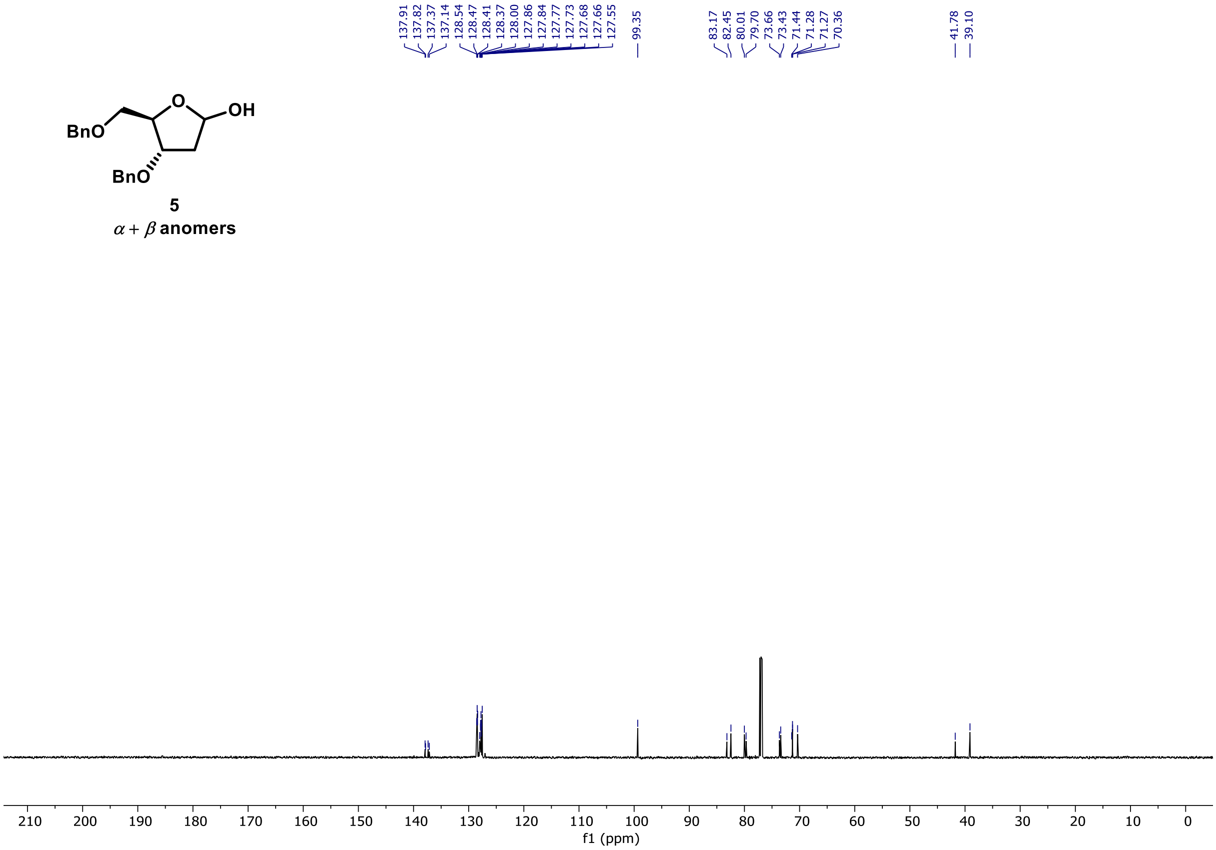

5

$\alpha + \beta$ anomers

8. References

1. Szcześniak, P.; Furman, B., Photo-Fries-type rearrangement of cyclic enamides. An efficient route to structurally diverse five-membered enaminones. *Chemical Communications* **2022,** *58*, 1898-1901.
2. van Rijssel, E. R.; van Delft, P.; van Marle, D. V.; Bijvoets, S. M.; Lodder, G.; Overkleeft, H. S.; van der Marel, G. A.; Filippov, D. V.; Codée, J. D. C., Stereoselectivity in the Lewis Acid Mediated Reduction of Ketofuranoses. *The Journal of Organic Chemistry* **2015,** *80*, 4553-4565.
3. Zhuang, J.-J.; Ye, J.-L.; Zhang, H.-K.; Huang, P.-Q., An unexpected high erythro-selection in the Grignard reaction with an N,O-acetal: a concise asymmetric synthesis of indolizidine alkaloid (−)-2-epi-lentiginosine. *Tetrahedron* **2012,** *68*, 1750-1755.
4. Stöver, M.; Lützen, A.; Köll, P., New glyco-oxazolidin-2-ones as chiral auxiliaries in boron-mediated asymmetric aldol reactions. *Tetrahedron: Asymmetry* **2000,** *11*, 371-374.
5. Matsuura, D.; Mitsui, T.; Sengoku, T.; Takahashi, M.; Yoda, H., Stereodivergent synthesis of new amino sugars, furanodictines A and B, starting from d-glucuronolactone. *Tetrahedron* **2008,** *64*, 11686-11696.
6. Heuer, M.; Hohgardt, K.; Heinemann, F.; Kühne, H.; Dietrich, W.; Grzelak, D.; Müller, D.; Welzel, P.; Markus, A.; Heijenoort, Y. v.; Heijenoort, J. v., Structural analogues of the antibiotic moenomycin a with a D-glucose-derived unit F. *Tetrahedron* **1994,** *50*, 2029-2046.
7. Kim, I. S.; Zee, O. P.; Jung, Y. H., Regioselective and Diastereoselective Amination of Polybenzyl Ethers Using Chlorosulfonyl Isocyanate: Total Syntheses of 1,4-Dideoxy-1,4-imino-d-arabinitol and (−)-Lentiginosine. *Organic Letters* **2006,** *8*, 4101-4104.
8. Liu, H.; Li, X., Synthesis of protected sugar-amino acid hybrid molecules as platform for further derivatization. *Tetrahedron Letters* **2012,** *53*, 6957-6960.
9. Ben Jamaa, A.; Grellepois, F., Diastereoselective Ritter-like Reaction on Cyclic Trifluoromethylated N,O-Acetals Derived from l-Tartaric Acid. *The Journal of Organic Chemistry* **2017,** *82*, 10360-10375.
10. Yahata, K.; Sakurai, S.; Hori, S.; Yoshioka, S.; Kaneko, Y.; Hasegawa, K.; Akai, S., Coupling Reaction between Aldehydes and Non-Activated Hydrocarbons via the Reductive Radical-Polar Crossover Pathway. *Organic Letters* **2020,** *22*, 1199-1203.
11. Wang, H.-H.; Shao, H.; Huang, G.; Fan, J.; To, W.-P.; Dang, L.; Liu, Y.; Che, C.-M., Chiral Iron Porphyrins Catalyze Enantioselective Intramolecular C(sp3)−H Bond Amination Upon Visible-Light Irradiation. *Angewandte Chemie International Edition* **2023,** *62*, e202218577.
12. Frisch, M. J.; Trucks, G. W.; Schlegel, H. B.; Scuseria, G. E.; Robb, M. A.; Cheeseman, J. R.; Scalmani, G.; Barone, V.; Petersson, G. A.; Nakatsuji, H.; Li, X.; Caricato, M.; Marenich, A. V.; Bloino, J.; Janesko, B. G.; Gomperts, R.; Mennucci, B.; Hratchian, H. P.; Ortiz, J. V.; Izmaylov, A. F.; Sonnenberg, J. L.; Williams; Ding, F.; Lipparini, F.; Egidi, F.; Goings, J.; Peng, B.; Petrone, A.; Henderson, T.; Ranasinghe, D.; Zakrzewski, V. G.; Gao, J.; Rega, N.; Zheng, G.; Liang, W.; Hada, M.; Ehara, M.; Toyota, K.; Fukuda, R.; Hasegawa, J.; Ishida, M.; Nakajima, T.; Honda, Y.; Kitao, O.; Nakai, H.; Vreven, T.; Throssell, K.; Montgomery Jr., J. A.; Peralta, J. E.; Ogliaro, F.; Bearpark, M. J.; Heyd, J. J.; Brothers, E. N.; Kudin, K. N.; Staroverov, V. N.; Keith, T. A.; Kobayashi, R.; Normand, J.; Raghavachari, K.; Rendell, A. P.; Burant, J. C.; Iyengar, S. S.; Tomasi, J.; Cossi, M.; Millam, J. M.; Klene, M.; Adamo, C.; Cammi, R.; Ochterski, J. W.; Martin, R. L.; Morokuma, K.; Farkas, O.; Foresman, J. B.; Fox, D. J. *Gaussian 16 Rev. C.01*, Wallingford, CT, 2016.
13. Chai, J.-D.; Head-Gordon, M., Long-range corrected hybrid density functionals with damped atom–atom dispersion corrections. *Physical Chemistry Chemical Physics* **2008,** *10*, 6615-6620.
14. Zhao, Y.; Truhlar, D. G., The M06 suite of density functionals for main group thermochemistry, thermochemical kinetics, noncovalent interactions, excited states, and transition elements: two new functionals and systematic testing of four M06-class functionals and 12 other functionals. *Theoretical Chemistry Accounts* **2008,** *120*, 215-241.
15. Marenich, A. V.; Cramer, C. J.; Truhlar, D. G., Universal Solvation Model Based on Solute Electron Density and on a Continuum Model of the Solvent Defined by the Bulk Dielectric Constant and Atomic Surface Tensions. *The Journal of Physical Chemistry B* **2009,** *113*, 6378-6396.
16. Legault, C. Y. CYLview20, Université de Sherbrooke, 2020. (http://www.cylview.org).
